# 3DeFDR: statistical methods for identifying cell type-specific looping interactions in 5C and Hi-C data

**DOI:** 10.1186/s13059-020-02061-9

**Published:** 2020-08-28

**Authors:** Lindsey R. Fernandez, Thomas G. Gilgenast, Jennifer E. Phillips-Cremins

**Affiliations:** 1 Department of Bioengineering, University of Pennsylvania, Philadelphia, PA 19104 USA; 2 Epigenetics Institute, Perelman School of Medicine, University of Pennsylvania, Philadelphia, PA 19104 USA; 3 Department of Genetics, Perelman School of Medicine, University of Pennsylvania, Philadelphia, PA 19104 USA

**Keywords:** 3D chromatin looping interactions, Higher-order chromatin architecture, Epigenetics, Chromosome-conformation-capture, Chromatin dynamics, False discovery rate

## Abstract

An important unanswered question in chromatin biology is the extent to which long-range looping interactions change across developmental models, genetic perturbations, drug treatments, and disease states. Computational tools for rigorous assessment of cell type-specific loops across multiple biological conditions are needed. We present 3DeFDR, a simple and effective statistical tool for classifying dynamic loops across biological conditions from Chromosome-Conformation-Capture-Carbon-Copy (5C) and Hi-C data. Our work provides a statistical framework and open-source coding libraries for sensitive detection of cell type-specific loops in high-resolution 5C and Hi-C data from multiple cellular conditions.

## Introduction

Chromosome-Conformation-Capture (3C)-based molecular techniques have recently been coupled with high-throughput sequencing to generate genome-wide maps of higher-order chromatin folding [1–3]. A number of massively parallel 3C-based technologies query genome folding in a protein-independent manner, including Hi-C, 4C, 5C, and Capture-C [4–10]. All four techniques rely on proximity ligation and high-throughput sequencing to convert physically connected chromatin fragments into counts of specific interaction events. Briefly, chromatin is fixed in its native architectural state across a population of cells and then digested with a restriction enzyme. Restriction fragments are ligated to form billions of hybrid ligation junctions between two distal genomic loci. The two fragments in a given ligation junction can then be identified using high-throughput sequencing, and their frequency of ligation is proportional to their spatial proximity across a population of cells. Hi-C detects all chromatin interactions genome-wide using high-throughput sequencing, whereas 5C and Capture-C use tiled probes to selectively sequence large, megabase-scale subsets of the genome. 4C queries all genome-wide contacts involving a single chosen restriction fragment. Thus, the protein-independent 3C technologies of Hi-C, 5C, and Capture-C can be used to create high-resolution spatial maps of genome folding on the scale of a few megabases to genome-wide coverage.

Recently published 3C-based sequencing studies have revealed that the mammalian genome is folded into a hierarchy of distinct architectural features, including A/B compartments, lamina-associated domains (LADs), topologically associating domains (TADs), subTADs, and long-range looping interactions [6, 8, 10–19]. Loops—groups of adjacent pixels which form a punctate focal increase in interaction frequency enriched above local TAD and subTAD structure—have been identified algorithmically in high-resolution Hi-C maps [11]. The highest resolution maps to date have enabled the detection of tens of thousands of looping interactions genome-wide [11, 20]. A subset of looping interactions occur at the corners of TADs/subTADs and are known as “corner dots.” A leading model for the mechanism of corner dot formation is that cohesin tracks along the chromatin fiber until it is blocked by the architectural protein CTCF, thus extruding out the intervening DNA [21–26]. Corner dot TADs/subTADs anchored by CTCF are thought to demarcate the search space of enhancers for their target promoters [27–31]. Moreover, enhancers can also connect directly to target genes via corner dots in a CTCF-dependent and CTCF-independent manner [32–35]. Initial studies have suggested that specific subsets of looping interactions can reconfigure in development, disease, and in response to genetic perturbations [20, 29, 32, 33, 36–42]. Generally, however, it remains unknown to what extent loops are dynamically altered genome-wide as cells switch fate, due in part to the relative paucity of computational methods to evaluate statistically significant changes in interaction frequency across multiple biological conditions.

As high-resolution Hi-C and 5C chromatin folding maps begin to accumulate in developmentally relevant cellular models, there is an increasing need for methods to (1) precisely detect loops and clearly distinguish them from other classes of architectural features such as local TAD/subTAD structure and compartments and (2) rigorously classify loops by their dynamic behavior across cell types. A number of computational methods report the ability to identify loops in individual libraries generated by Hi-C. Bicciato and colleagues performed a detailed comparison of Hi-C loop calling pipelines, including HiCCUPS [43], GOTHiC [44], HOMER (http://homer.ucsd.edu/homer/interactions/), diffHic [45], HIPPIE [46], and Fit-Hi-C [47]. The conclusion from this study was that loop calling methods in individual samples exhibit vastly different performance, with no clear gold standard emerging [48]. Importantly, most loop calling pipelines were developed on low-resolution maps (40 kb up to 1 Mb bins) generated with the first-generation dilution Hi-C experimental procedure. More recently, Hi-C maps have achieved 1–5-kb resolution through higher read depth and markedly reduced spatial noise due to second generation in situ ligation and digestion techniques [11, 20]. We also note that active, unsynchronized extrusion events could create long-range interactions within TADs/subTADs that do not manifest as punctate loops in a 5C/Hi-C heatmap (i.e., transient loops in the making) [24]. Thus, it is likely that first generation loop calling algorithms show a wide dynamic range of performance because they were developed on lower resolution first-generation Hi-C maps and did not explicitly distinguish loops from general non-specific, long-range interactions. The emerging model from second-generation Hi-C studies is that quantitative loop detection in individual libraries requires rigorous modeling of local chromatin domain structure. HiCCUPS explicitly models and accounts for locus-specific TAD/subTADs [11], and accounting for local chromatin domain structure has therefore emerged as a leading candidate for identifying bona fide loop structures (i.e., persistent loops) in individual Hi-C maps. Building upon advances in Hi-C, similar statistical methodologies have been applied in lib5C to find loops in individual 5C maps [49].

To our knowledge, computational tools are not yet available to statistically test loops for their differential signal across two or three conditions in 5C data. Three tools (diffHic [45], FIND [50], and HiBrowse [51]) have been published to identify generally differential interactions between conditions in Hi-C data. All three methods in their published, first-generation form were not designed or verified to distinguish loops from higher-order folding patterns such as A/B compartments, TADs, subTADs, or non-specific long-range interactions [50]. In the absence of accounting for these features, a large proportion of the differential interactions identified may be due to cell type-specific fluctuations related to technical biases, local chromatin domains, extrusion lines, or higher-order compartments. Noteworthy, the diffHic manuscript indicates that modeling local chromatin domain structure would be essential to evaluate cell type-specific loops, suggesting that second-generation tools which accomplish this might be available in the future [45]. Computational tools have also been published to call within- and across-condition loops from libraries generated by Hi-ChIP and ChiA-PET assays [52–57]. However, statistical frameworks built for protein-dependent 3C-methods cannot address the technical challenges unique to 5C and Hi-C data. Overall, a gold-standard statistical methodology for cell type differential loop detection in protein-independent proximity ligation data (both 5C and Hi-C) is an important unmet need.

Here, we present 3DeFDR, a new statistical method and software implementation for identifying cell type-specific looping interactions from genome-wide Hi-C (3DeFDR-HiC) and locus-specific 5C (3DeFDR-5C) data across two or three biological conditions. For locus-specific 5C matrices, 3DeFDR-5C computes an empirical false discovery rate (eFDR) by applying a thresholding scheme on the change in interaction score signal on real 5C libraries from multiple biological conditions and pseudo-replicates simulated from the same biological condition. We implement a controlling procedure in which we iterate thresholds to achieve an a priori determined eFDR under the assumption that all thresholded pseudo-replicate interactions simulated from the same condition are false positives. For genome-wide Hi-C matrices, 3DeFDR-HiC formulates a negative binomial likelihood ratio test parameterized with a Distance-Dispersion-Relationship (DDR) for every pixel engaged in persistent loops genome-wide. Cell type-specific loops called by 3DeFDR-5C have fewer false positives and are more strongly enriched for chromatin modifications characteristic of the cellular state in which the loops are present compared to (i) an established ANOVA test and (ii) our own newly formulated parametric likelihood ratio test (3DLRT). We also benchmarked 3DeFDR-HiC against the leading published Hi-C non-specific differential interaction calling method diffHic and demonstrate superior performance. 3DeFDR-5C, 3DeFDR-HiC, and the parametric benchmarking test 3DLRT are freely available as Python packages to support the next wave of discoveries in cell type-specific looping.

## Results

**Fig. 1:**
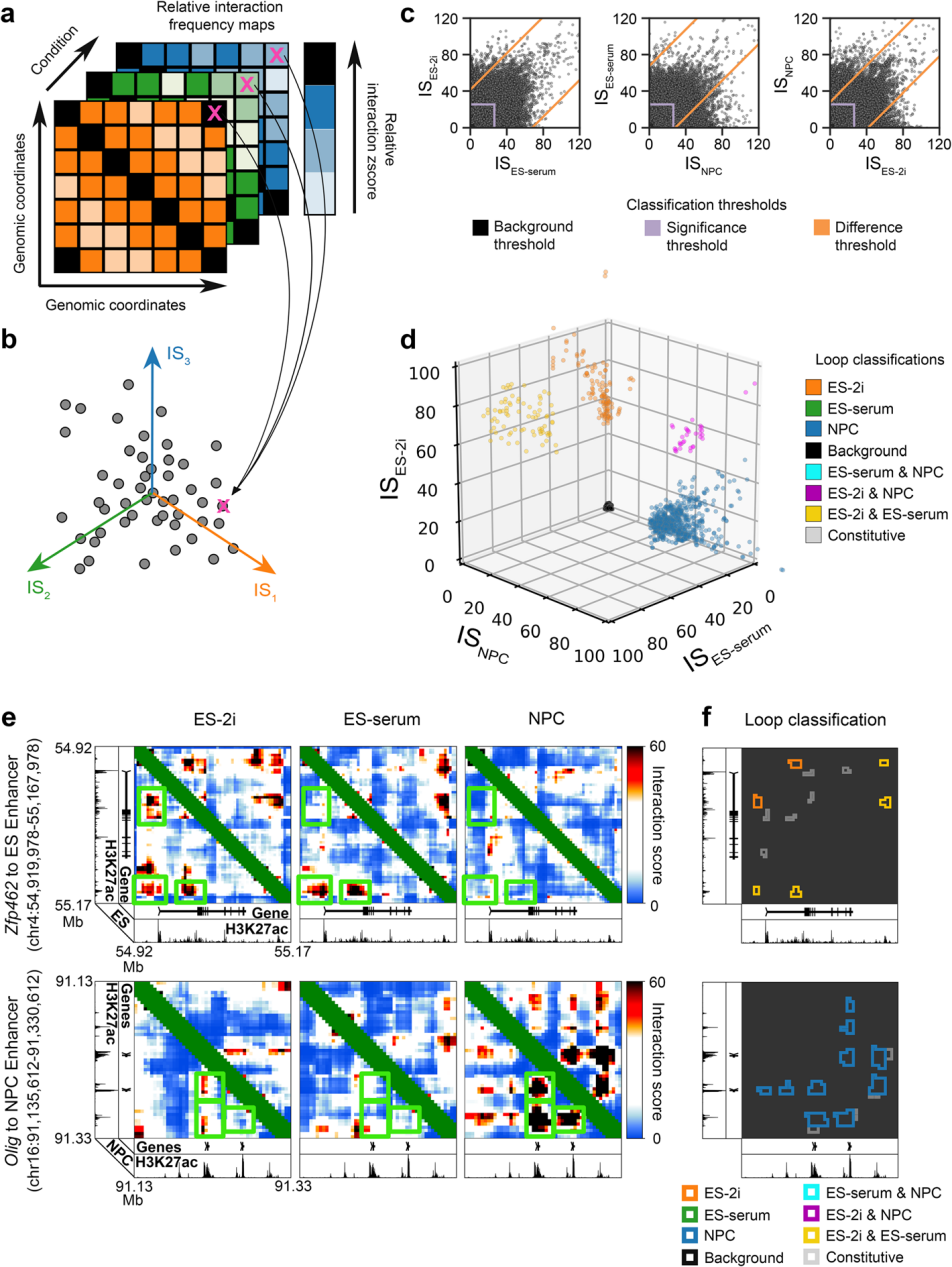
Overview of interaction score thresholding procedure for cell type-specific looping interaction classification. **a** A 5C dataset is input as a set of interaction frequency matrices, with each matrix capturing the same set of genomic contacts under a different cellular condition. **b** Raw 5C counts are converted to interaction scores (IS) which reflect bias-corrected, sequencing depth normalized, local expected background signal normalized, and statistically modeled interaction frequency values that are comparable within and between conditions under the assumptions of our model (detailed in the “Methods” section and Fig. 4). **c** Interaction scores are thresholded to allow detection and classification of looping interactions that are significantly differential across cellular conditions. **d** Seven looping interaction classes after a 3-way thresholding scheme on ES-2i, ES-serum, and NPC cellular states. **e** IS heatmaps at two selected genomic loci. Green boxes highlight regions of qualitatively apparent differences in looping signal. **f** Loop classification results after applying 3DeFDR-5C’s 3-way IS thresholding procedure

We set out to address a critical challenge in the analysis of looping interactions in 5C data: the paucity of methods for robustly classifying dynamic loops across multiple cellular conditions, a problem which becomes more challenging as the number of conditions increases. Our goal was to develop a statistical framework and software implementation to rigorously identify differential loops from 5C maps across two or three conditions using a target FDR to choose thresholds (Fig. 1a).

First, we developed, applied, and benchmarked 3DeFDR-5C using 5C data across three distinct cellular states: mouse embryonic stem (ES) cells cultured in 2i media representing a naive pluripotent state, mouse ES cells cultured in LIF/serum representing the primed pluripotency state, and primary neural progenitors isolated from neonatal mice representing a multipotent adult stem cell state in the neuroectoderm lineage (Additional file 1: Table S1) [32]. These particular 5C datasets represent large-scale, 4-kb-resolution maps capturing 8 Mb of genomic sequence around key developmentally regulated genes. 5C relies on a primer-based hybrid capture step to selectively detect ligation junctions across specific genomic regions, thus enabling the creation of high-resolution matrices with a strikingly lower number of reads (~ 30–40 million per sample) compared to Hi-C (~ 3–6 billion per sample). We have recently determined that loops are markedly reconfigured during the transition from naive pluripotency to multipotency, thus making this dataset ideal for the testing and development of our statistical framework. We tested and validated 3DeFDR-5C with a three cellular state experiment, but the statistical framework and code are also able to analyze a two cellular state experiment.

We first started by modeling and correcting biases, artifacts, and local chromatin domains in individual replicates. Despite their nuanced technical differences, data from protein-independent proximity ligation techniques share several common features, including: (1) distance-dependent background interaction signal in which non-specific interaction frequency is highest for the closest fragment-fragment pairs on the linear genome and decays as the distance separating the genomic fragments increases [6], (2) biases in ligation and amplification frequency caused by GC content and length of restriction fragments [58, 59], (3) library complexity and sequencing depth differences across independent experiments for the same biological sample leading to nonlinear batch effects [60], and (4) highly locus-specific structure due to higher-order folding of chromatin into TADs, subTADs, and compartments [11]. One must model and address these features to ensure a rigorous analysis of looping interactions.

We reasoned that a differential loop calling method would have the most utility across protein-independent proximity ligation data if it started with a modified interaction score (IS) in which background signal as well as per-replicate and per-pixel confounding factors had been corrected. We recently discovered that sequence-related biases are not constant across cell types and replicates. Therefore, as is routinely done with Hi-C data, we used matrix balancing to correct for fragment-specific biases caused by GC content and restriction fragment length for every replicate individually (detailed in the “Methods” section). We also used conditional quantile normalization to normalize all replicates for non-linear library complexity and sequencing depth differences (detailed in the “Methods” section). It is widely known that the distance-dependent background signal and local chromatin domain structure are widely variable across cell types and highly unique to each genomic region. Thus, we used the donut and lower left filters [11, 32] to model the distance-dependent and TAD/subTAD expected background signal for every interaction in the genome and every replicate individually (detailed in the “Methods” section). After bias correction, background normalization, and expected modeling, we assigned *p* values to every pixel in the 5C heatmap and computed an interaction score (IS) that allows for direct comparison of each bin-bin pair across replicates and conditions under the assumption that the replicates are similarly powered (Fig. 1). Moreover, the use of modeled IS as the random variable for differential testing allows 3DeFDR to have utility for matrices of any protein-independent 3C-based data that have been bias corrected, normalized, modeled, and transformed into *p* values using analysis techniques tailored to the specific method.

To identify differential looping interactions, we used a classification technique that relies on three-way thresholding on the difference in IS across cellular conditions (Fig. 1, Additional file 2: Fig. S1, Additional file 3: Table S2). For each biological replicate, we began with a framework in which IS is a square, symmetric matrix of interaction scores from a modeled and bias-corrected 5C experiment. The matrix IS has dimensions *n* by *n*, where *n* is the number of genomic bins in any particular genomic region, *r*. We use ${\mathrm{IS}}_{t_s,r,k,l}$ to refer to the interaction score between genomic bins *k* and *l* in region *r* as recorded for biological replicate *s* of condition *t* (detailed in the “Methods” section). We first identify potential looping interactions by parsing only bin-bin interactions with an ${\mathrm{IS}}_{t_s,r,k,l}$ greater than a specific significance threshold *g* for all replicates in at least one condition (purple lines, Fig. 1). We then apply a series of thresholds (orange lines, Fig. 1c) on the difference in ${\mathrm{IS}}_{t_s,r,k,l}$ across all three cellular conditions (Additional file 2: Fig. S1E-G, Additional file 3: Table 2S). To ensure the most conservative estimate of looping classes, we apply the thresholds on the minimum difference in IS across replicates of each condition. Thus, the end result is a preliminary set of seven classes of looping interactions: (1) ES-2i only, (2) ES-serum only, (3) NPC only, (4) ES-2i and ES-serum only, (5) ES-2i and NPC only, (6) ES-serum and NPC only, and (7) constitutive across all three cell types (Fig. 1). Examples of ES-2i only, ES-2i and ES-serum only, and NPC only interactions are illustrated in Fig. 1, f.

We next used estimation and control of an empirical false discovery rate (eFDR) to guide the final placement of the difference thresholds for each looping class (orange lines, Fig. 1c, detailed in the “Methods” section). The false discovery rate (FDR) is by definition FDR = E[*V*/*R*] where *V* is the number of false positives among tests declared significant and *R* is the total number of tests declared significant. Here, *R* is trivial to compute from our set of three conditions (*T* = {*A*, *B*, *C*} where *A* is ES-2i, *B* is ES-serum, and *C* is NPC) and six replicates (*S* = {*A*1, *A*2, *B*1, *B*2, *C*1, *C*2}) as the total number of significant bin-bin interactions in a given looping class (*H* = {{*A*}, {*B*}, {*C*}, {*A*, *B*}, {*A*, *C*}, {*B*, *C*}}). However, *V* is not known and requires a method for estimating the false-positive rate of our three-way thresholding procedure.

We hypothesized that *V* is approximately equal to the total number of interactions labeled as differential when applying 3DeFDR-5C to a set of biological samples with no true differential loops. We defined our null dataset as a set of samples that are replicates of a single cellular condition but are assigned a random set of labels matching conditions *T*. Our key assumption in formulating this approach is that that the false-positive rate (FPR) of calls on the null dataset (FPR_null_) is approximately equivalent to that of the experimental dataset (FPR_exp_), such that FPR_null_ ≈ FPR_exp_. We computed and controlled an empirical false discovery rate (eFDR) as in Eq. 1:

1 $$ \mathrm{eFDR}=\frac{n_{\mathrm{null}}}{n_{\mathrm{exp}}}\approx \frac{V}{R} $$

where *n*_exp_ is the total number of interactions classified as significantly differential for a particular looping class using the experimental conditions *T* and *n*_null_ is the total number of interactions classified as significantly differential in the null dataset, which approximates FPR_exp._

It is often cost prohibitive to generate six biological replicates of 5C data for each condition. Therefore, we generated 5C replicate simulations to populate the null sample set. We simulated 5C replicates of the same condition at the level of fragment-fragment ligation counts after conditional quantile normalization. Our rationale for this decision was that it would allow us to omit library complexity, batch effect, and sequencing depth terms in our count generating models. To construct our simulation generating model, we first computed the sample mean and sample variance for every interaction in every condition (Equations 2 and 3):

2 $$ {\mu}_{t,r,i,j}=\frac{\sum_{s=1}^{n_t}{C}_{t_s,r,i,j}^{\prime }}{n_t} $$

3 $$ {\sigma}_{t,r,i,j}^2=\frac{\sum_{s=1}^{n_t}{\left({C}_{t_s,r,i,j}^{\prime }-{\mu}_{t_s,r,i,j}\right)}^2}{n_t-1} $$

where *n*_*t*_ is the number of replicates of condition *t* and ${C}_{t_s,r,i,j}^{\prime }$ is the conditional quantile normalized 5C counts of interaction (*t*,*r*,*i*,*j*) in the *s*^th^ replicate of condition *t* for every *i*^th^ and *j*^th^ fragment ligation in genomic region *r*.

**Fig. 2:**
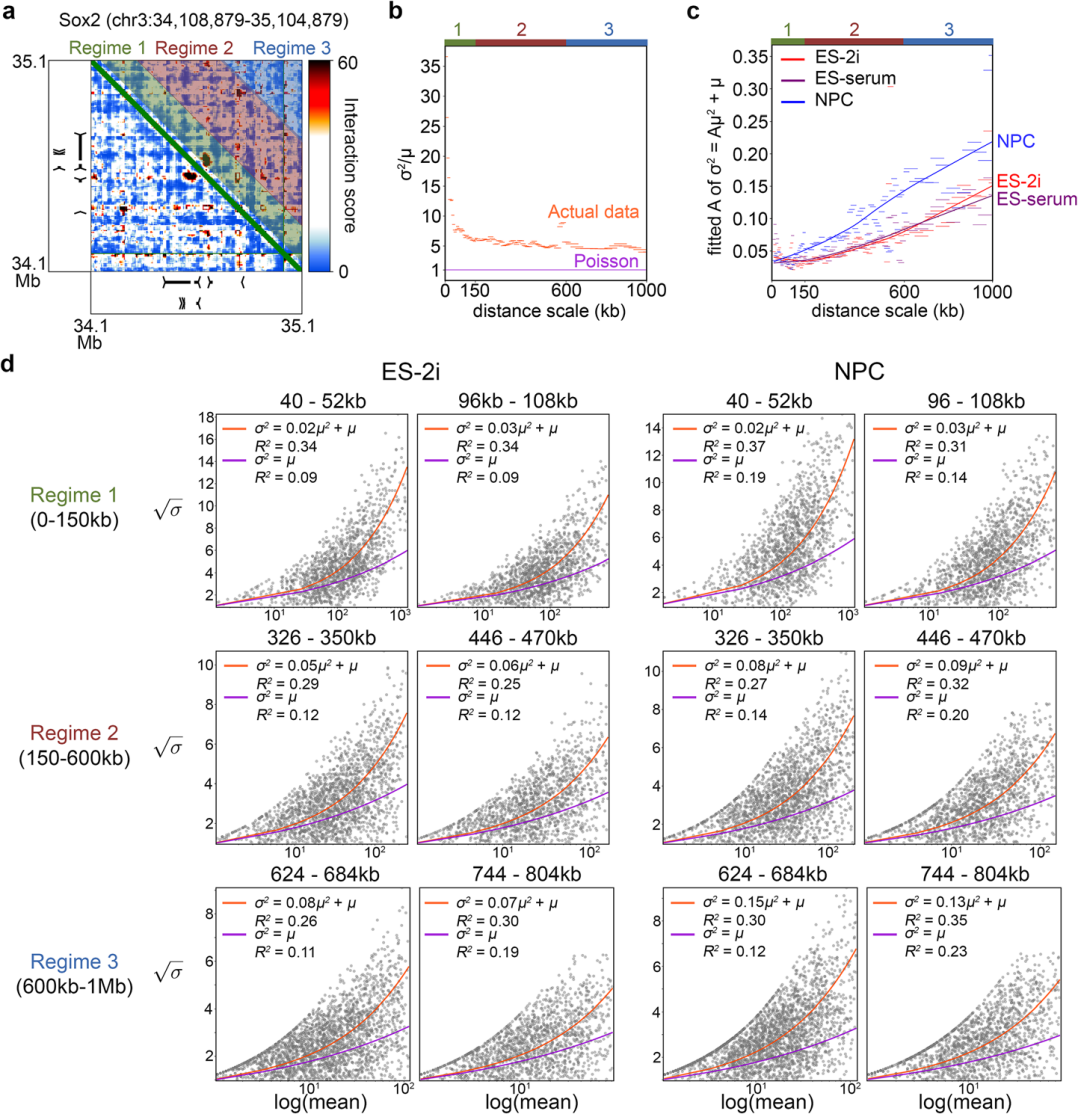
5C counts are overdispersed and their mean-variance relationship varies as a function of linear genomic distance and cellular condition. **a** Raw 5C contacts are stratified by genomic distance prior to characterization of their mean-variance relationship. In each of our three regimes, the width of the stratification windows is determined using a different binning scheme. **b** The coefficient of variation for raw 5C counts is plotted against the median genomic interaction distance for each sliding window. Each window captures counts from all genomic regions in the dataset in the ES-2i condition. **c** The dispersion parameter, *A*, for each distance scale window (short horizontal lines) is computed by fitting sample means and variances to the function σ^2^ = A*μ^2^ + μ. Dispersion versus distance scale trends (solid smooth lines) were generated by Loess smoothing. **d** Mean-variance models for representative genomic distance windows from all three distance regimes. Fits of the Poisson mean-variance relationship (*σ*^2^ = μ) and the negative binomial mean-variance relationship (*σ*^2^ = A*μ^2^ + μ) are shown with their corresponding *R*^2^ goodness of fit values

Most genomics experiments suffer from poor parameter estimation due to the low number of replicates that are financially and logistically feasible to generate for every biological condition. To improve parameter estimates, we modeled the mean-variance relationship (MVR) between *μ*_*t*,*r*,*i*,*j*_ and *σ*^2^_*t*,*r*,*i*,*j*_ by pooling all interactions at similar interaction distances (Fig. 2). We stratified quantile normalized counts ${C}_{t_s,r,i,j}^{\prime }$ for all regions by their linear genomic interaction distance using a dynamic size window (Fig. 2a). For distance regime 1 (0–150 kb), we stratified the interactions into fine-grained, 12-kb-sized sliding windows with a 4-kb step. For distance regime 2 (151–600 kb), we stratified the interactions into 24-kb-sized sliding windows with an 8-kb step. For distance regime 3 (601–1000 kb), we stratified the interactions into coarse-grained, 60-kb-sized sliding windows with a 24-kb step. We found that the variance was greater than the mean across all genomic distance scales, indicating that 5C counts data are overdispersed (Fig. 2). For each window in each distance regime, we modeled the MVR by fitting the function (Equation 4):

4 $$ {\hat{\sigma}}_{t,r,i,j}^2={A}_{t,w}{\mu}_{t,r,i,j}^2+{\mu}_{t,r,i,j} $$

to all *μ*_*t*,*r*,*i*,*j*_ and *σ*^2^_*t*,*r*,*i*,*j*_ to find the overdispersion parameter, *A*_*t*,*w*_, at each distance scale (detailed in the “Methods” section). We found that *A*_*t*,*w*_ also varied as a function of distance and was unique to each cell type (Fig. 2). Together, these data demonstrate that 5C counts are overdispersed and that the overdispersion parameter varies as a function of distance and cellular state.

To generate simulated 5C libraries, we weighted the predicted variance ${\hat{\sigma}}_{t,r,i,j}^2$ against the original observed variance *σ*^2^_*t*,*r*,*i*,*j*_ to generate a final weighted variance ${\overline{\sigma}}_{t,r,i,j}^2$ for each interaction at each distance scale as in Equation 5 (detailed in the “Methods” section):

5 $$ {\overline{\sigma}}_{t,r,i,j}^2=\alpha {\hat{\sigma}}_{t,r,i,j}^2+\beta {\sigma^2}_{t,r,i,j} $$

We used *α* = *β* = 0.5 to achieve simulated 5C counts with pairwise correlations on par with that of real replicates while improving the quality of our variance estimate with the predicted contribution (Additional file 4: Table S3). Finally, we parameterized the negative binomial model for each *C*′_*t*,*r*,*i*,*j*_ interaction and generated simulated counts from our models for each (*t*,*r*,*i*,*j*) interaction (Equation 6):

6 $$ {C}_{t,r,i,j}^{\prime \mathrm{sim}}\sim \mathrm{NB}\left({\mu}_{t,r,i,j},{\overline{\sigma}}_{t,r,i,j}^2\right) $$

**Fig. 3:**
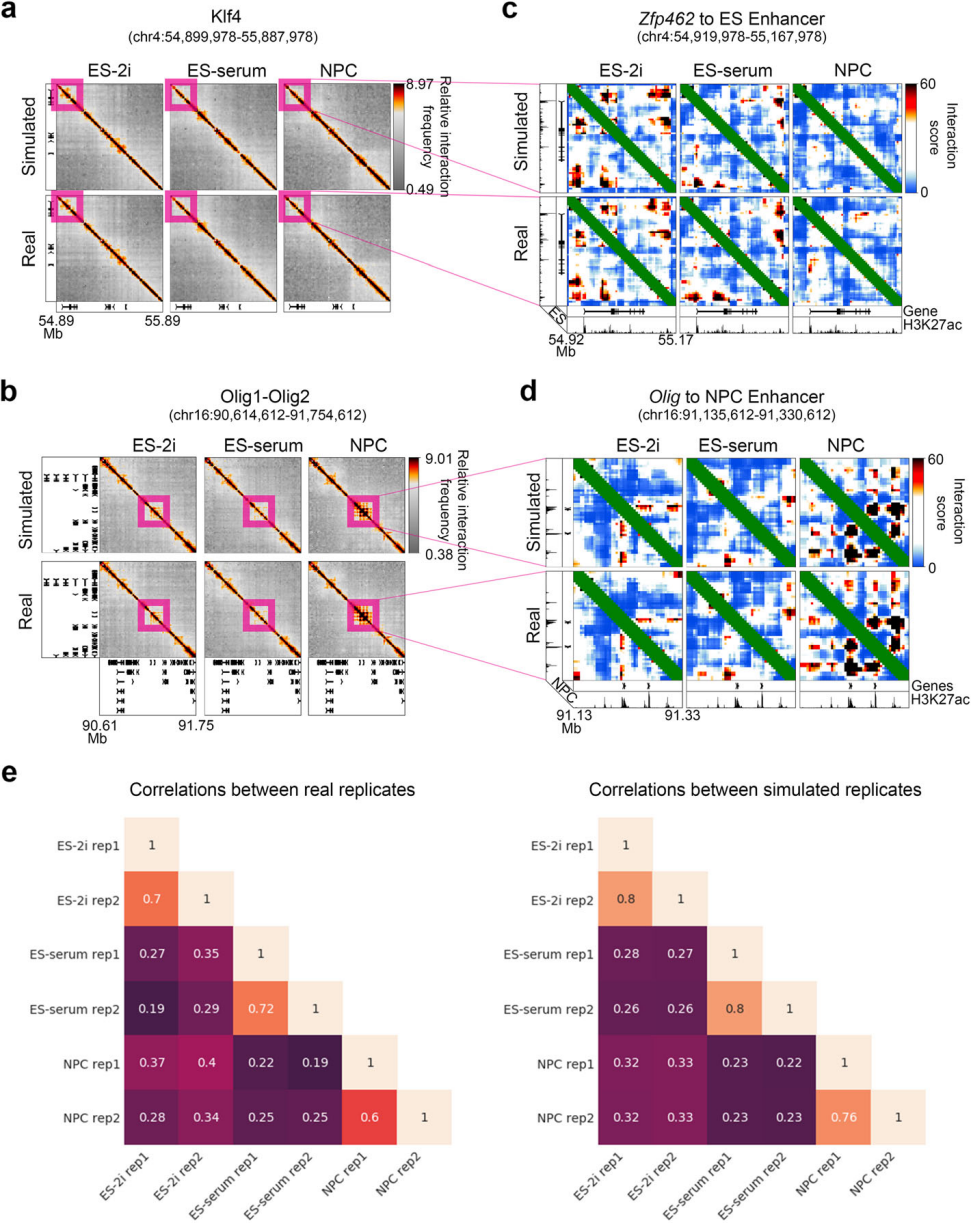
Simulated 5C datasets exhibit strong similarity to experimental 5C datasets. **a**, **b** Heatmaps of relative 5C interaction frequency in the genomic regions surrounding the **a** *Klf4* and **b** *Olig1/2* genes are shown for simulations and real experimental data. **c**, **d** Heatmaps of interaction scores in the genomic regions surrounding the **c** *Klf4* and **d** *Olig1/2* genes are shown for simulations and real experimental data. **e** Matrices of pairwise Spearman’s correlations between real and simulated 5C replicates after conditional quantile normalization (see the “Methods” section)

We created simulated replicates by filling in a simulated counts value for each (*t*, *r*, *i*, *j*) interaction with a random variable drawn from the negative binomial distribution parameterized by *μ*_*t*,*r*,*i*,*j*_ and ${\overline{\sigma}}_{t,r,i,j}^2$. We then subjected the simulated 5C libraries, ${C}_{t,r}^{\prime \mathrm{sim}}$, to the same matrix balancing, binning, expected normalization, and modeling as the real 5C libraries (see the “Methods” section). Simulated 5C counts were highly similar to real 5C data in a qualitative comparison (Fig. 3a–d, Additional file 2: Fig. S2). Moreover, for the final predicted variance estimates (Equation 5 weighted at *α* = *β* = 0.5), our simulated 5C libraries exhibit Spearman’s correlations within and between conditions that are nearly equivalent to real replicates (Fig. 3). Together, these data show that 5C libraries can be simulated with a negative binomial distribution parameterized with an overdispersed distance-specific MVR.

**Fig. 4:**
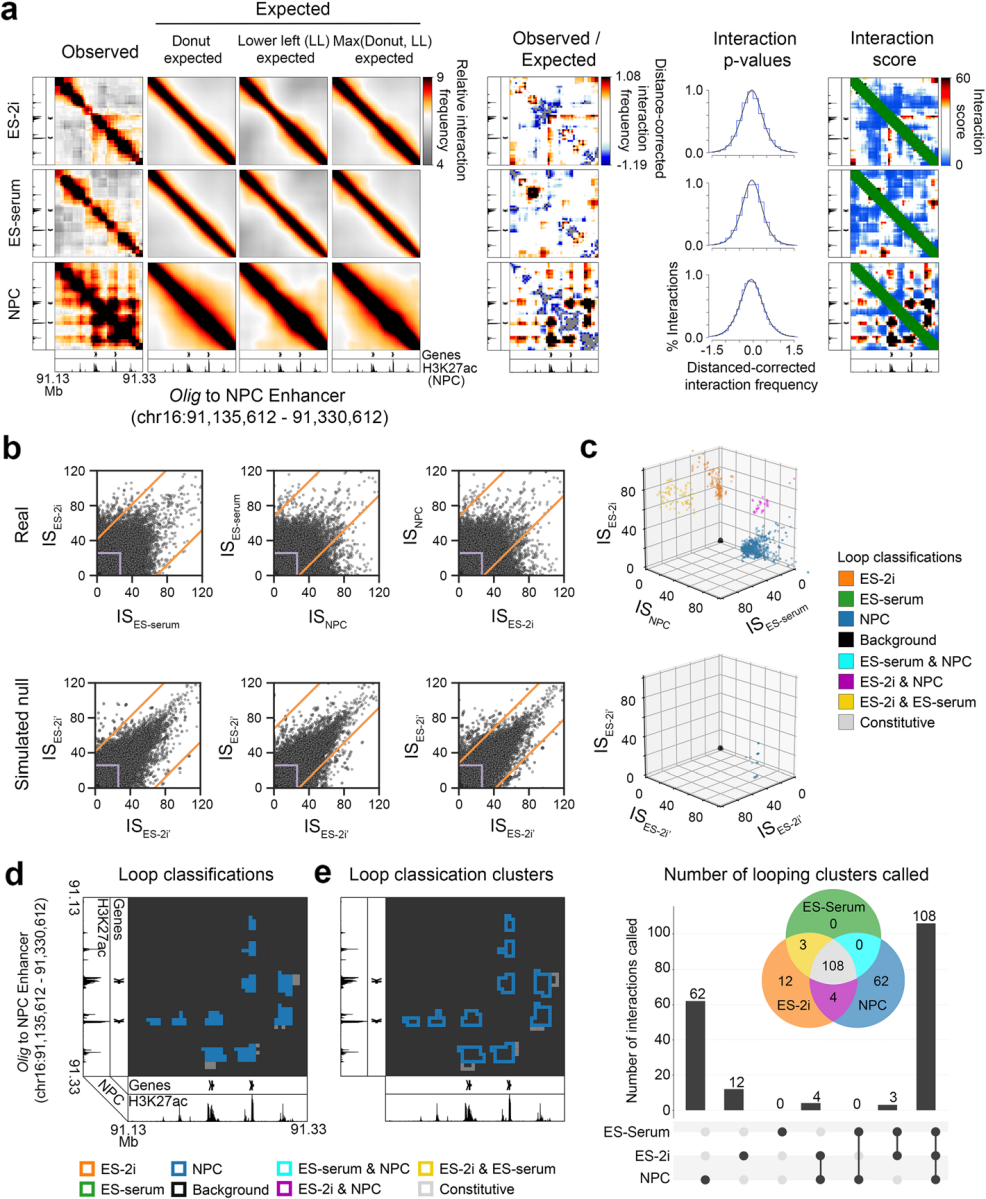
Application of 3DeFDR-5C to find cell type-specific looping interactions across three cellular states. **a** Heatmaps representing binned, matrix balanced 5C counts (Observed) around a known looping interaction between the *Olig1* gene and an NPC-specific enhancer (chr16:91,135,612-91,330,612). Observed counts are divided by the computed local expected signal to obtain background-normalized counts (Observed/Expected). These counts are fitted with a logistic distribution and the resulting *p*-values are transformed into interaction scores, where interaction score = − 10*log2(*p* value). **b** Interaction scores are thresholded to isolate contacts that are differentially looping across cellular conditions and whose signal meets a baseline requirement for significance. This thresholding procedure is applied to both real and simulated null replicate sets to compute an eFDR estimate. The dynamic thresholding procedure is applied with increasing stringency until a user-specified target false discovery rate is reached. **c** Loop classifications obtained with 3DeFDR-5C in real (top) and simulated null (bottom) replicate sets shown in an interaction scatterplot representation. **d**, **e** Heatmap of final loop classifications at **d** individual bin-bin pairs and **e** classified looping clusters after applying 3DeFDR-5C at a threshold of 2%. **f** UpsetR scalable Venn diagrams for differential looping clusters called by 3DeFDR-5C at a target eFDR of 2%

**Fig. 5:**
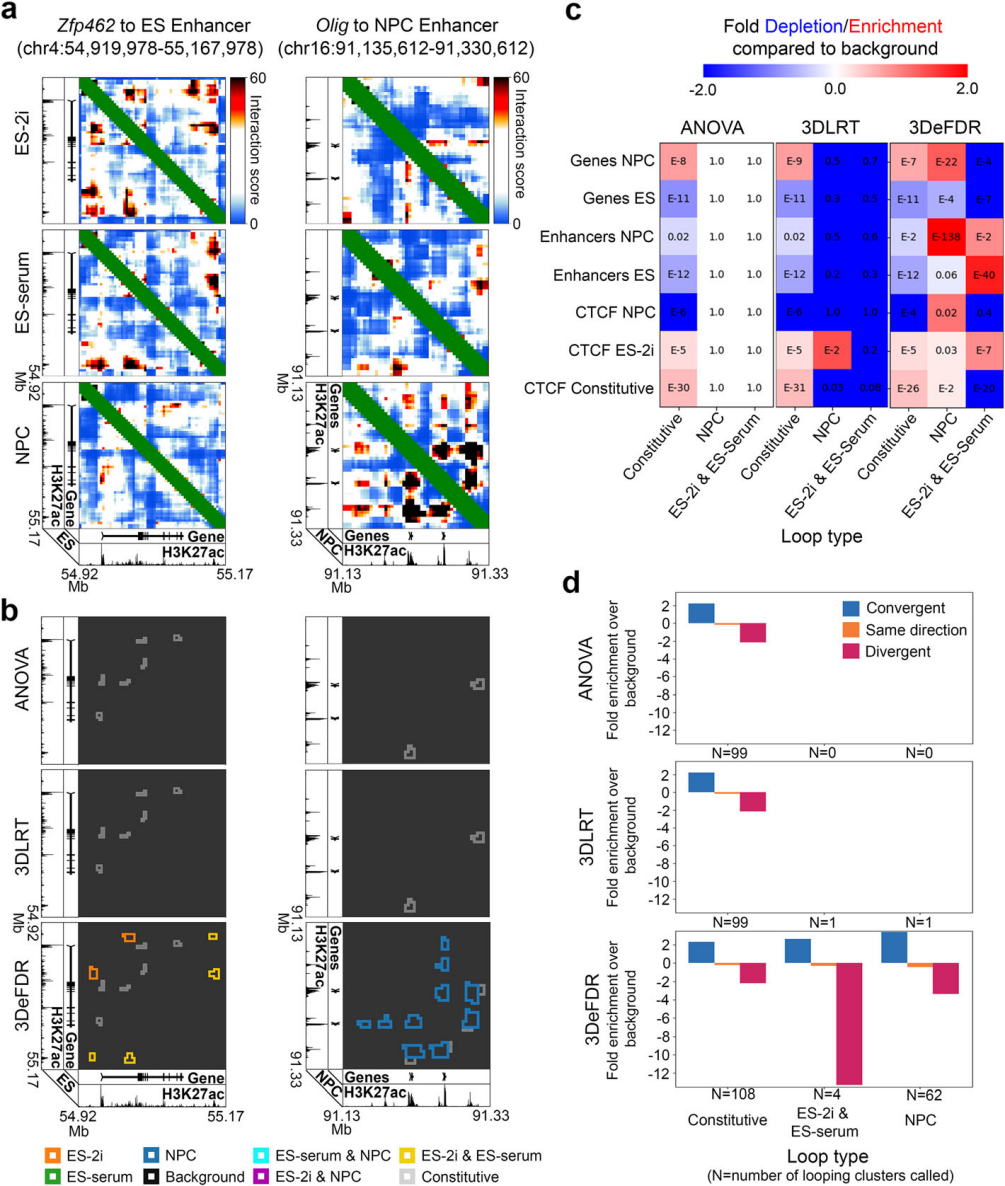
Dynamic 3D chromatin looping interactions identified using 3DeFDR-5C, 3DLRT, and ANOVA. **a** Reference interaction score heatmaps for two sample loci. **b** Loop classification results achieved with each differential looping detection method at a target false discovery rate (FDR) of 2%. **c** Enrichment of cell-type specific markers in loops classified as NPC or ES-2i & ES-serum for each of the three methods at a target FDR of 2%. **d** Log fold-change in percent CTCF orientation among loops classified as constitutive, ES-2i & ES-serum, or NPC, over percent CTCF orientation among loops classified as background

We next used simulated IS matrices (Fig. 4a) to compute an empirical FDR (eFDR) estimate for our looping classes across a sweep of IS difference thresholds applied to both real (${\mathrm{IS}}_{t_s,r,k,l}$) and simulated (${\mathrm{IS}}_{t_s,r,k,l}^{\mathrm{sim}}$) values. For each loop classification, we computed eFDR estimates across a range of difference threshold values *d*, acquiring a difference threshold-to-eFDR mapping for each class, eFDR_*d*, *h*_, as in Equation 7:

7 $$ {\mathrm{eFDR}}_{d,h}=\frac{\operatorname{card}\left(\left\{\left(r,k,l\right)\in {h}_{\mathrm{null}}^d\right\}\right)}{\operatorname{card}\left(\left\{\left(r,k,l\right)\in {h}_{\mathrm{exp}}^d\right\}\right)} $$

where ${h}_{\mathrm{null}}^d$ represents the set of interactions assigned to differential class *h* in the simulated null dataset at difference threshold *d* and ${h}_{\mathrm{exp}}^d$ represents the set of interactions assigned to the same class in the real experimental dataset at the same difference threshold *d*. We selected our final eFDR threshold *τ* as 2% (Figs. 4 and 5). We performed this eFDR controlling procedure for every differential looping class across our three cellular states to identify significantly differential bin-bin pairs (Fig. 4). We then clustered significantly differential bin-bin pairs of a similar looping class by spatial adjacency (see the “Methods” section); the end result was 108 constitutive, 12 ES-2i only, 62 NPC only, 3 ES-2i and ES-Serum, and 4 ES-Serum and NPC looping clusters (Fig. 4). The 3DeFDR-5C algorithm is designed so that the user can tune the final looping classifications to a pre-determined target eFDR.

To evaluate the performance of the 3DeFDR-5C pipeline, we implemented two additional methods for classifying differential looping interactions: ANOVA-BH and 3DLRT-BH (Additional file 2: Fig. S3). These methods use ANOVA and our newly formulated likelihood ratio test (3DLRT), respectively, to assign a differential looping *p* value to every bin-bin pair in an experimental dataset (detailed in the “Methods” section). In both approaches, output *p* values are then corrected for multiple testing using the Benjamini-Hochberg step-up procedure. When we compared ANOVA and 3DLRT benchmarking tests to 3DeFDR-5C, we found that the three different methods had different optimal FDR thresholds for identifying differential loops (Supplementary Figures 4–6, 8–10), with 3DeFDR-5C identifying the known, previously reported looping interactions at significantly lower FDR estimates than the other two approaches (Fig. 5). Thus, 3DeFDR-5C can identify known cell type-specific looping interactions with a lower estimated false discovery rate than ANOVA and 3DLRT benchmarking tests under the assumptions of our model.

To further understand the dynamic loops called by 3DeFDR-5C, we also compared them to chromatin modifications on the 1-D genome as well as to the performance of the leading non-specific differential interaction caller built for Hi-C data. We observed that classes of differential loops identified by 3DeFDR-5C at an FDR of 2% were strongly enriched for genes and enhancers characteristic of cell types matching their differential loop class (Fig. 5c, Additional file 2: Figs. S7, S11). Moreover, we observed that convergently and divergently oriented CTCF motifs were over- and under-enriched, respectively, at the base of loops identified by 3DeFDR-5C (Fig. 5). Together, these data indicate that 3DeFDR-5C calls differential loops that exhibit the known hallmarks of cell type-specific looping interactions.

**Fig. 6:**
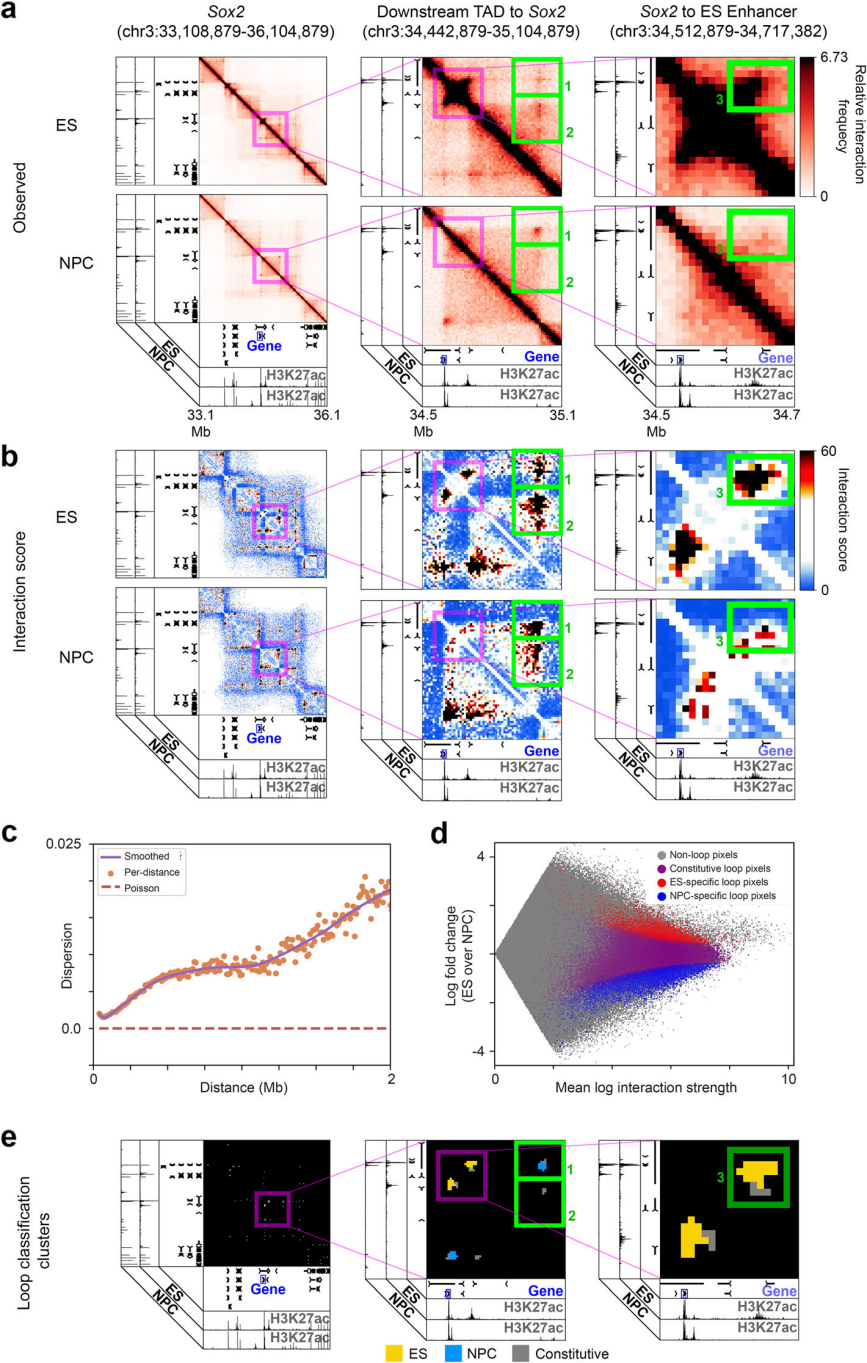
Cell-type specific looping interactions identified from Hi-C using 3DeFDR-HiC. **a** Reference heatmaps of relative Hi-C interaction frequency (Observed) for the *Sox2* region and two zoom-in views of loops involving the *Sox2* gene. Boxes 1, 2, and 3 highlight areas of differential looping. **b** Reference interaction score heatmaps of the same genomic regions shown in **a**. **c** Distance-dispersion relationship in the ES condition in the Bonev et al. Hi-C dataset. The orange dots show the estimated negative binomial dispersion parameter at each distance scale. The purple line represents a LOWESS smoothing of the orange points. The red dashed line shows the effective dispersion of the Poisson distribution for comparison. **d** MA plot of the differential loop analysis comparing the ES and NPC conditions in the Bonev et al. Hi-C dataset. The *x*- and *y*-axes represent the average log interaction frequency and the log fold change across cell types, respectively, computed on observed Hi-C counts normalized for both locus specific biases and sequencing depth differences. The densities of non-loop, constitutive, and differential (called by our method at an FDR threshold of 1%) pixels are shown in different colors as indicated in the legend. **e** Heatmaps of final loop cluster classifications for each genomic region called by 3DeFDR-HiC at an FDR threshold of 1%

Finally, we formulated 3DeFDR-HiC to identify cell type-specific loops genome-wide in Hi-C data. To develop 3DeFDR-HiC, we relied on ultra-high-resolution Hi-C data from mouse ES cells and ES-derived NPCs [61]. We first identified loops genome-wide in each cell type individually (see the “Methods” section, Fig. 6a, b). To identify which of the identified loops were ES- or NPC-specific, we formulated a negative binomial model parameterized by (i) the mean count per pixel across replicates for every biological condition, (ii) a distance-dependent scaling factor to normalize for sequencing depth (Additional file 2: Fig. S14), (iii) bias factors for every row in the raw Hi-C matrix, and (iv) an estimated dispersion per pixel across replicates for every biological condition (see the “Methods” section). We estimated the dispersion of loops at every 10-kb increment of genomic distance via a distance-dispersion-relationship (DDR) (see the “Methods” section, Fig. 6). After fitting the parameters of our model to the data, we performed a likelihood ratio test to obtain *p* values against the null hypothesis that each interaction in a loop was not differential and applied the Benjamini-Hochberg step-up procedure to correct these *p* values for multiple testing. At an FDR threshold of 1% and a loop cluster size threshold of 3 (see the “Methods” section), we identified 818 ES-specific loops and 1435 NPC-specific loops (Fig. 6), including the ES-specific loop connecting the *Sox2* gene to its ES-specific enhancer (box 1), and the longer-range ES-specific, NPC-specific, and constitutive loops around *Sox2* at this locus (box 2, box 3) (Fig. 6 b, e). Thus, we can identify cell type-specific looping interactions genome-wide in Hi-C data with 3DeFDR-HiC.

Our 3DeFDR-HiC method makes three critical assumptions: (1) the use of a negative binomial distribution is necessary to account for overdispersion in Hi-C data, (2) the model needs to account for the DDR, and (3) pooling dispersion or variance estimates is necessary to achieve good performance in the face of small numbers of available replicates. To test these three assumptions, we benchmarked the performance of 3DeFDR-HiC on simulated data against three alternative models that each dropped one of our three assumptions. These alternative models included a Poisson model (which assumes mean is equal to variance with no overdispersion), a “global negative binomial” model (which does not account for the DDR), and a “sample variance parameterized negative binomial” model (which does not pool dispersion or variance estimates and uses a sample variance computed for each pixel across replicates instead) (see the “Methods” section). We provide the intuition for how each of the three models compares to our 3DeFDR-HiC method in Additional file 2: Fig. S13A. Our inspection of distributions of *p* values called on true null simulations revealed that the Poisson model failed to control type I error (Additional file 2: Fig. S13B). This failure to control type I error was also reflected in a failure to control FDR in simulations containing truly differential loops (Additional file 2: Fig. S13C). Next, we assessed the performance of the different approaches using receiver operator characteristic (ROC) curves, revealing that the “sample variance parameterized negative binomial” model resulted in inferior cell type-specific loop classification performance compared to 3DeFDR-HiC, which uses pooled dispersion estimates (Additional file 2: Fig. S13D). Finally, we assessed the bias of low *p* values in simulated null datasets with respect to distance (Additional file 2: Fig. S13E), revealing that the “global negative binomial” model is overly conservative at short distances, where it overestimates dispersion, and overly permissive at long distances, where it underestimates dispersion. Altogether, these results were used to formulate and justify the assumptions upon which we built our 3DeFDR-HiC model.

**Fig. 7:**
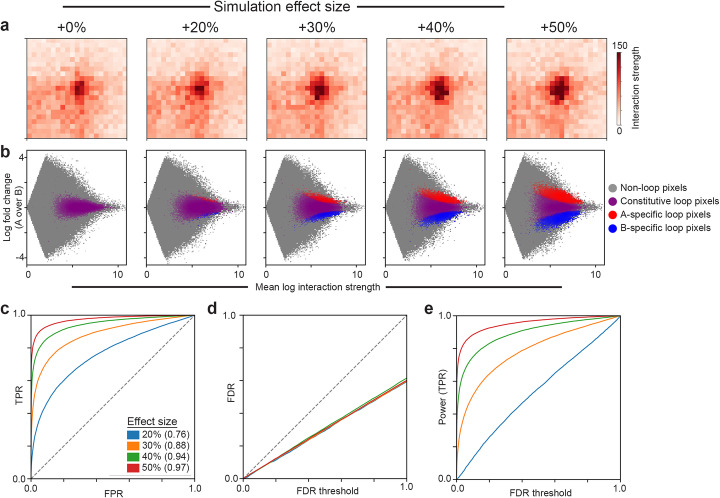
Characterization of performance of 3DeFDR-HiC method using simulated Hi-C data. **a** Heatmaps showing a single example loop in simulations generated using varying effect sizes. The difference between any heatmap and the baseline loop strength shown in the far-left panel becomes more pronounced as effect size increases. **b** MA plots resulting from analysis of simulations of two artificial conditions (“A” and “B”) generated using varying effect sizes, with red and blue points representing interactions called as differential by our method at a false discovery rate of 1%. No interactions are called differential when no loops are truly differential (effect size + 0%). The number of interactions called as differential increases with increasing effect size, though the true proportion of differential interactions remains fixed at 40% in the simulations shown here. **c** Receiver operating characteristic (ROC) curves showing performance of our method on simulations generated using varying effect sizes. Like in **b**, the true proportion of differential interactions remains fixed at 40%. The *x-*axis shows the false-positive rate (FPR), or one minus the specificity. The *y*-axis shows the true positive rate (TPR), or sensitivity. The area under the receiver operating characteristic curve (AUROC) for each curve is shown in parentheses in the legend. **d** False discovery rate (FDR) control curves showing FDR control characteristics of our method on simulations generated using varying effect sizes, colored as in (C). The *x*-axis shows a range of FDR thresholds, while the *y*-axis shows the actual FDR we observe in the differential calls made by our method at that FDR threshold. Methods that control FDR should stay below the dashed gray line. All FDR control curves should show an FDR of 60% at an FDR threshold of 100%, since only 40% of loops in each simulation are truly differential. **e** Power curves showing the proportion of truly differential interactions called differential by our method (*y*-axis) as a function of the FDR threshold used for thresholding (*x*-axis) in simulations generated using varying effect sizes, colored as in **c**

Finally, to benchmark 3DeFDR-HiC’s performance, we applied diffHic [45] to the same Hi-C data. When comparing the two methods, we held constant either the FDR threshold or the total number of significant differential loops. In both the ‘matched FDR’ and ‘matched loop number’ benchmarking scenarios, we observed that diffHic called cell type-specific interactions throughout Hi-C data irrespective of whether or not the interactions were bona fide loops (Additional file 2: Fig. S12A,C). We also created simulated Hi-C maps containing pre-defined cell type dynamic looping interactions with a range of interaction strength effect sizes (see the “Methods” section, Fig. 7). 3DeFDR-HiC markedly outperformed diffHic in the sensitivity and specificity of differential loops called on our simulated datasets (Additional file 2: Fig. S12D). As expected, running 3DeFDR-HiC on simulations with stronger looping fold changes resulted in a higher number of differential loops called (Fig. 7). 3DeFDR-HiC exhibits strong sensitivity and specificity of loop detection which increases with increasing interaction frequency effect size (Fig. 7), as well as consistently strong FDR control at every tested interaction frequency effect size (Fig. 7). Our simulations can be used to perform power calculations at a variety of effect sizes (Fig. 7), providing estimates of the proportion of uncalled truly differential loops across a range of differential effect sizes. Together, these data characterize the performance of 3DeFDR-HiC and suggest that it outperforms the leading Hi-C interaction caller diffHiC.

## Conclusion

Since the invention of 5C and Hi-C technologies, the field has been in need of statistical methods and computational tools for identifying differential long-range looping interactions among biological conditions. To date, there is a severe lack of differential loop calling methods available for analysis of 5C data by the scientific community. Moreover, although a small number of “general differential interaction identification” methods have been published for Hi-C data, differential loop calling largely remains an open question because (1) currently available tools do not account for local distance-dependent background signal and TAD/subTAD/compartment structure to identify changes specifically at loops and (2) Hi-C datasets with the resolution necessary for looping interaction analysis have only very recently become available. We describe two variants of our method: 3DeFDR-5C, our original approach designed for identifying cell type-specific loops from 5C data, and 3DeFDR-HiC, a simplified and parallelized variant fast enough to identify differential loops in genome-wide Hi-C datasets.

It is important to acknowledge potential limitations in our methods. 3DeFDR-5C and 3DeFDR-HiC cannot in their current form detect global changes in looping due to a biological perturbation such as nuclear volume change which would lead to global shift in signal at a specific distance scale. We have created our code in a way that allows users to alter bias vectors and scaling parameters to account for their biological question. In cases of global changes, the normalization and correction of samples together would not be preferred. We also acknowledge that our work here represents one of the first in-depth studies of the problem of variance estimation in Hi-C data. To further enhance differential loop calling performance, newer modeling approaches will be needed to improve upon our dispersion estimates in the future. In an ideal scenario, Hi-C data for every condition would be obtained with a high number of biological replicates, thus facilitating the ability to estimate variance on a per-pixel basis and account for the local TAD/subTAD and compartment folding patterns that influence mean and variance estimates at each pixel. Here, we pool interaction frequencies by distance to create a DDR, but future studies may reveal that dispersion is controlled by additional factors beyond distance and biological condition.

In this study, we analyze Hi-C datasets using a 10-kb bin resolution. In principle, our implementation of 3DeFDR-HiC is fast enough to call differential loops using smaller bin sizes; however, we have chosen to present results using 10 kb bins due to the scarcity of Hi-C datasets with sufficient read depth to reliably detect loops at bin sizes smaller than 10 kb. We expect that assessing the < 10-kb bin matrix resolution performance of 3DeFDR-HiC and other differential loop calling models will become an important area for future work as more ultra-high-resolution Hi-C datasets become available.

Our analyses thus far have suggested that variance estimation is not as critical for differential loop calling genome-wide in “C” data as it is for differential gene expression analyses in RNA-seq data. Our hypothesis for this discrepancy is that RNA-seq data has a much higher dynamic range of counts than “C” data and that the dispersion estimates matter most for modeling very highly expressed genes. Consistent with this idea, we do indeed observe that both of our methods (3DeFDR-5C and 3DeFDR-HiC) allow for more sensitive and specific loop detection in the case of ultra-short-range loops where the interaction frequencies have the highest mean. The advantage is, however, small compared to using per-pixel sample variances or a zero-dispersion Poisson model, and future studies will unravel how improved sensitivity/specificity in loop calling will aid in biological discovery in high-resolution Hi-C data. A systematic comparison of all differential looping models—including a more quantitative performance assessment for 5C differential loop calling—remains an important area for future work.

In conclusion, we provide 3DeFDR as a new statistical framework and computational tool for detecting and classifying differential looping interactions in high-resolution, multi-condition 5C and Hi-C datasets. We note that the performance of 3DeFDR is highly dependent on the quality of the input dataset and how effectively the raw sequencing counts of detected interactions have been processed to reduce batch effects, correct for bias, and account for distance-dependent and TAD/subTAD background signal. We provide 3DeFDR as a modular coding package that the user may integrate into their own 5C or Hi-C analysis pipeline. For the convenience of users, this package includes companion visualization tools for assessing 3DeFDR results to determine how effectively counts have been modeled for simulation, viewing differential loop calls as color-coded clusters, and computing the enrichment of classical epigenetic marks within classes of called loops.

## Methods

### 5C data

5C libraries generated with a single alternating primer design [32] in embryonic stem (ES) cells cultured in 2i media (ES-2i), ES cells cultured in serum/LIF (ES-Serum), and primary.

### Hi-C data

Hi-C libraries were downloaded from GEO (Additional file 6: Table S5). Briefly, we used all raw Hi-C sequencing reads from the ES_1, ES_3, NPC_1, and NPC_2 replicates (representing the ES and NPC conditions), keeping the replicates separate.

### 5C data processing pipeline

#### Overview

Raw 5C counts were subjected to our previously published 5C count modeling methods [32, 60, 62–65]. The processing steps described briefly below ultimately resulted in the conversion of fragment-level, raw count matrices to a bias- and expected background-corrected contact matrices of interaction scores. Pre-processing steps were performed prior to the post-processing steps of matrix balancing, binning, mean-variance relationship modeling, and 5C replicate simulation. Binning and all subsequent normalization and modeling steps were performed on both experimental and simulated 5C replicates.

#### Data structure and pre-processing

We assembled sequencing counts from each 5C experiment *t*_*s*_ and each genomic region *r* into an *n*_*r*_ × *n*_*r*_ raw contact matrix, ${C}_{t_s,r}$, where *n*_*r*_ represents the total number of HindIII restriction fragments in each region *r*, *t* ∈ {ES2i, ESserum, NPC} represents a cellular condition, and *s* ∈ {1, 2} represents a biological replicate of the cellular condition *t*. Thus, ${C}_{t_s,r,i,j}$ is the number of reads that represent contacts between the *i*th and *j*th fragments in region *r*, where *i* ∈ {1, 2, 3, …, *n*_*r*_} and *j* ∈ {1, 2, 3, …, *n*_*r*_}. Raw contact matrices were then normalized as described [32]. Briefly, the raw contact matrices ${C}_{t_s,r}$ were normalized for replicate biases due to batch effects, sequencing depth differences, and library complexity differences by conditional quantile normalization to create a normalized contact matrix ${C}_{t_s,r}^{\prime }$.

#### Matrix balancing

Each normalized contact matrix ${C}_{t_s,r}^{\prime }$ was then matrix balanced with joint express as described [32, 49] to correct for differences in fragment-specific biases, such as GC content, fragment length, and 5C primer-specific efficiency at each primer in region *r* to create a balanced contact matrix ${C}_{t_s,r}^{\prime }$.

#### Contact matrix binning

Balanced contact matrices ${C}_{t_s,r}^{\prime }$ were converted to binned interaction frequency matrices by binning at regular 4-kb intervals and smoothing at 16-kb intervals as described in [32, 49, 62]. The smoothing was performed because we developed the 3DeFDR-5C method on older 5C data from an alternating 5C primer design. 5C libraries made with double alternating designs do not require this smoothing step [64]. The resulting binned interaction frequency matrices ${B}_{t_s,r}$ have *m*_*r*_ by *m*_*r*_ elements where *m*_*r*_ is the total number of bins in region *r*. ${B}_{t_s,r,k,l}$ represents the arithmetic mean contact frequency between fragments in the *k*th and *l*th bins in genomic region *r* as recorded in replicate *s* under condition *t*. Binned interaction frequency matrices have reduced spatial noise relative to the original fragment-level matrices while preserving the underlying signal.

#### Distance dependence normalization

Following binning, expected values for each interaction in the binned interaction frequency matrices were computed using a modification of the local donut expected described by Aiden and colleagues that accounts for the local TAD/subTAD structure and the global distance-dependent background signal [11, 32, 49]. The binned interaction frequency values ${B}_{t_s,r,k,l}$ (Observed) were corrected by the maximum of expected donut values ${\mathrm{DE}}_{t_s,r,k,l}$ and expected lower left values ${\mathrm{LLE}}_{t_s,r,k,l}$ to yield contact enrichments (Observed/Expected, or Obs/Exp) normalized for distance-dependent 5C count signal and local chromatin domain structure as detailed previously [32].

#### Probabilistic model fitting

As detailed previously [32], contact enrichment values (Obs/Exp) were modeled within each region by parameterizing a log-logistic distribution using maximum likelihood estimation, resulting in matrices of right-tailed *p* values ${P}_{t_s,r}$. *P* values were computed for each 5C genomic region separately.

#### Removal of interactions below distance limit

Interactions occurring between bins within 20 kb of each other on the linear chromatin fiber were removed from consideration and not included in further processing.

#### Interaction scores and *z*-scores

The final step of the post-processing pipeline is the conversion of modeled *p* values to interaction scores. We use ${\mathrm{IS}}_{t_s,r}$ to refer to the matrix of interaction scores for region *r* and replicate *s* in condition *t*. For 3DeFDR-5C, *p* values were transformed to an interaction score of −10 × log_2_(*p* value). For benchmarking approaches, ANOVA, and 3DLRT (detailed below), *p* values were transformed to both an interaction score of −10 × log_2_(pvalue) as well as a *z*-score computed using the standard normal quantile function (the inverse of the standard normal cumulative distribution function) (Equations 8 and 9):

8 $$ \varPhi (z)=\frac{1}{\sqrt{2\pi }}{\int}_{-\infty}^z{e}^{-\frac{x^2}{2}} dx $$

9 $$ {Z}_{t_s,r,k,l}\kern0.5em ={\varPhi}^{-1}\left(1-{P}_{t_s,r,k,l}\right) $$

where ${P}_{t_s,r,k,l}$ is the right-tail *p* value computed for the interaction between bins *k* and *l* in genomic region *r* as recorded in biological replicate *s* under condition *t*. We implemented the conversion of *p* values to *z*-scores using the stats.norm.isf function in the scipy Python library.

### Hi-C data processing pipeline

Raw Hi-C data were aligned to the mm9 genome using bowtie2 (global parameters: --very-sensitive -L 30 --score-min L,-0.6,-0.2 --end-to-end --reorder; local parameters: --very-sensitive -L 20 --score-min L,-0.6,-0.2 --end-to-end --reorder) through the HiC-Pro software. Unmapped reads, non-uniquely mapped reads, and PCR duplicates were filtered out, and uniquely aligned reads were paired. *Cis* contact matrices were assembled by binning paired reads into uniform 10 kb bins.

### 3DeFDR-5C

#### Overview

3DeFDR-5C is designed to identify differential looping interactions across a set of 5C experiments containing either two or three cellular conditions with at least two replicates each. In this section, we describe the application of 3DeFDR-5C to three cellular conditions, referring to a set of three conditions *T* = {*A*, *B*, *C*} and of six replicates as *S* = {*A*1, *A*2, *B*1, *B*2, *C*1, *C*2}.

#### Differential loop categories

In the 3DeFDR-5C framework, the set of possible classes of differential looping interactions is defined as all nonempty proper subsets *H* of the input condition set *T* (Equation 10):

10 $$ H=\left\{\left\{A\right\},\left\{B\right\},\left\{C\right\},\left\{A,B\right\},\left\{A,C\right\},\left\{B,C\right\}\right\} $$

Interactions assigned to single-condition classes, e.g., {*A*}, {*B*}, or {*C*}, are considered to be interacting significantly higher in replicates of that specific condition than in those of the other two conditions (Additional file 2: Fig. S1E and Additional file 3: Table S2). Interactions assigned to dual condition classes, e.g., {*A*, *B*}, {*A*, *C*}, or {*B*, *C*}, are considered to be interacting significantly higher in replicates of the two specific conditions than in the remaining single condition (i.e., *C*, *B*, and *A*, respectively). If interaction scores for an interaction are sufficiently high in all conditions, that interaction is interpreted to be non-differential and labeled as a constitutive looping interaction. If interaction scores are sufficiently low in all replicates of all conditions, the interaction is not called a looping interaction; therefore, it is not tested for differential looping signal (Additional file 2: Fig. S1D). Points with very low interaction scores in all replicates are assigned to a background class, representing interactions that are very unlikely to be loops (Additional file 2: Fig. S1C).

#### Computing empirical false discovery rate

3DeFDR-5C controls an empirically estimated false discovery rate (eFDR) to classify loops as differentially interacting across cellular condition set *T*. By definition, $\mathrm{FDR}=\mathrm{E}\left[\frac{V}{R}\right]$ where *V* is the number of false positives among tests declared significant and *R* is the total number of tests declared significant. *R* is computed as the total number of pixels called as significantly differential in any differential class in *H* (Equation 10). By contrast, *V* is not trivially computed and requires a model for estimating what proportion of looping interactions in each class in *H* are false positives.

We hypothesized that *V* is approximately equal to the total number of interactions incorrectly labeled as differential when applying 3DeFDR-5C (holding all of its thresholds fixed) to a set of biological samples known to have no truly differential loops (i.e., a null biological sample set). We defined our null data set as a set of samples that are all replicates of a single cellular condition but are assigned a set of labels matching the different conditions in *T*. The key assumption of this approach is that that the false-positive rate (FPR) of calls on the null dataset (FPR_null_) is approximately equivalent to that of the real experimental dataset (FPR_exp_), such that FPR_null_ ≈ FPR_exp_. We computed and controlled an empirical false discovery rate (eFDR) (Equation 11):

11 $$ \mathrm{eFDR}=\frac{n_{\mathrm{null}}}{n_{\mathrm{exp}}}\approx \frac{V}{R} $$

where *n*_exp_ is the total number of interactions classified as significantly differential in the real experimental dataset and *n*_null_ is the total number of interactions classified as significantly differential in the null dataset.

We computed a piecewise interaction score thresholding scheme for each looping interaction class in the set of possible differential classifications *H*. To classify dynamic loops, 3DeFDR-5C applies a thresholding scheme based on the difference in interaction scores between conditions (Fig. 1). Using a sweep of IS difference thresholds *d* (see orange lines in Fig. 1), 3DeFDR-5C assigns every pixel in a 5C data set either to one of the differential classes in *H* or to the background, constitutive, or “other” class (as described above and below) and computes a class-specific eFDR for each differential looping interaction class *h* ∈ *H* as (Equation 12):

12 $$ {\mathrm{eFDR}}_{d,h}\approx \frac{n_{\mathrm{null}}^{d,h}}{n_{\mathrm{exp}}^{d,h}} $$

where ${n}_{\mathrm{exp}}^{d,h}$ is the total number of interactions assigned to differential looping class *h* in the real experimental dataset at difference threshold *d* and ${n}_{\mathrm{null}}^{d,h}$ is the total number of interactions assigned to differential looping class *h* in the simulated null dataset at the same difference threshold *d*. 3DeFDR-5C adapts the distance threshold for each differential looping class to maintain a user-specified target empirical FDR threshold *τ* across all differential looping classes. For each looping interaction class *h*, we determined the distance threshold *d* at which eFDR_*d*, *h*_ is closest to *τ* while remaining less than *τ*. Thus, each differential looping class *h* will have a unique difference threshold *d* to reach the study-specific target eFDR threshold *τ*. Constitutive looping pixels are identified as those that are strong in all conditions and are not sufficiently differential to admit assignment to one of the differential classes (Additional File 3: Table S2). “Other” or “uncalled” pixels include those that pass the looping threshold but do not meet the requirements of any of the other classes (Additional File 3: Table S2). Overall, 3DeFDR-5C employs eFDR estimate control to guide the placement of IS thresholds to call differential looping classes.

We did not have access to an experimental dataset with enough replicates of the same cellular condition to create a null replicate set directly from real 5C libraries. To avoid the high costs and labor required to run additional experiments, we modeled and created simulations of our existing experimental replicates to create additional simulated replicates. We constructed a null dataset from six simulated replicates (*S*_null_ = {*A*1_sim_, *A*2_sim_, *A*3_sim_, *A*4_sim_, *A*5_sim_, *A*6_sim_}) all based on the same biological condition (*T*_null_ = {*A*, *A*, *A*})).

#### Modeling and simulation of preprocessed replicates

We simulated 5C replicates of the same condition at the level of fragment-level counts after conditional quantile normalization. Our rationale for simulating quantile normalized counts rather than raw counts was that doing so would allow us to omit library complexity, batch effect, and sequencing depth terms in our count-generating models. We simulated fragment-resolution counts that have been quantile normalized but not balanced (resulting in simulated matrices comparable to ${C}_{t_s,r}^{\prime }$) by parameterizing a different negative binomial distribution for each interaction as described below and then drawing a random variable from this distribution.

To begin constructing our simulation-generating model, we computed the sample mean and sample variance of the preprocessed sample counts of a single interaction across replicates of the same condition as in Equations 13 and 14:

13 $$ {\mu}_{t,r,i,j}=\frac{\sum_{s=1}^{n_t}{C}_{t_s,r,i,j}^{\prime }}{n_t} $$

14 $$ {\sigma}_{t,r,i,j}^2=\frac{\sum_{s=1}^{n_t}{\left({C}_{t_s,r,i,j}^{\prime }-{\mu}_{t_s,r,i,j}\right)}^2}{n_t-1} $$

where *n*_*t*_ is the number of replicates of condition *t* and ${C}_{t_s,r,i,j}^{\prime }$ is the conditional quantile normalized 5C count value for the interaction between the *i*th and *j*th bins of region *r* in the *s*th replicate of condition *t*.

Most genomics experiments suffer from poor parameter estimation due to the low number of replicates that are financially and logistically feasible to generate for every biological condition. We did not use *μ*_*t*,*r*,*i*,*j*_ and ${\sigma}_{t,r,i,j}^2$, computed from only *n*_*t*_ = 2 replicates, to directly parameterize the negative binomial (NB) counts models for each quantile normalized interaction count ${C}_{t_s,r,i,j}^{\prime }$. Instead, we modeled the mean-variance relationship (MVR) between *μ*_*t*,*r*,*i*,*j*_ and ${\sigma}_{t,r,i,j}^2$, thereby leveraging the high-dimensional nature of our data set to improve our variance estimates. We stratified quantile normalized counts, ${C}_{t_s,r,i,j}^{\prime }$, for all regions by their linear genomic distance using overlapping stratification windows of different sizes depending on genomic distance. For distance regime 1 (0–150 kb), we stratified the interactions using fine-grained, 12-kb-sized sliding windows with a 4-kb step. For distance regime 2 (151–600 kb), we stratified the interactions into 24-kb-sized sliding windows with an 8-kb step. For distance regime 3 (601–1000 kb), we stratified the interactions into coarse-grained, 60-kb-sized sliding windows with a 24-kb step. For each window *w* in each distance regime, we modeled the MVR for each condition *t* by fitting the function *σ*^2^ = *A*_*t*,*w*_*μ*^2^ + *μ* to the *μ*_*t*,*r*,*i*,*j*_ and ${\sigma}_{t,r,i,j}^2$ values for all regions *r* and for all bin-bin pairs *i*, *j* whose linear genomic separation distance fell in window *w*. Prior to estimation of *A*_*t*,*w*_, interactions with mean counts of one or less, or more than 2.5 standard deviations above the mean of mean counts for interactions in bin *w* were removed. The dispersion parameters, *A*_*t*,*w*_, were then plotted as a function of genomic distance, and LOWESS smoothing with a smoothing fraction of 0.5 was used to compute the final dispersion estimates, ${\overline{A}}_{t,w}$. The predicted sample variance ${\hat{\sigma}}_{t,r,i,j}^2$ for each individual interaction was then computed using the LOWESS-smoothed dispersion estimate ${\overline{A}}_{t,w}$ appropriate for the window *w* corresponding to the interaction distance |*i* − *j*| as in Equation 15:

15 $$ {\hat{\sigma}}_{t,r,i,j}^2={\overline{A}}_{t,w}{\mu}_{t,r,i,j}^2+{\mu}_{t,r,i,j} $$

We weighted the predicted variance value ${\hat{\sigma}}_{t,r,i,j}^2$ against the original observed variance of the interaction ${\sigma}_{t,r,i,j}^2$ to generate a final weighted variance ${\overline{\sigma}}_{t,r,i,j}^2$ for each interaction as in Equation 16:

16 $$ {\overline{\sigma}}_{t,r,i,j}^2=\alpha {\hat{\sigma}}_{t,r,i,j}^2+\beta {\sigma}_{t,r,i,j}^2 $$

We chose to use *α* = *β* = 0.5 to achieve pairwise correlations on par with that of real replicates while improving the quality of our variance estimate with the predicted contribution. As shown in Additional file 4: Table S3, increasing *α* led to higher pairwise correlation between simulated replicates. Finally, we parameterized a negative binomial distribution for each *C*′_*t*,*r*,*i*,*j*_ interaction and generated simulated counts ${C}_{t,r,i,j}^{\prime \mathrm{sim}}$ from it as in Equation 17:

17 $$ {C}_{t,r,i,j}^{\prime \mathrm{sim}}\sim \mathrm{NB}\left({\mu}_{t,r,i,j},{\overline{\sigma}}_{t,r,i,j}^2\right) $$

#### Creating the null replicate set

Using the generative models described above, we created six simulated replicates of a chosen biological condition *t* ∈ *T*. For our results, we chose to use the NPC condition (which we will denote as condition *A* ∈ *T*), because this was the condition which showed the highest dispersion between replicates (Fig. 2) and would therefore result in the most conservative eFDR estimate. The simulated replicates that made up our null replicate set are shown in Equation 18:

18 $$ {S}_{\mathrm{sim}}=\left\{A{1}_{\mathrm{sim}},A{2}_{\mathrm{sim}},A{3}_{\mathrm{sim}},A{4}_{\mathrm{sim}},A{5}_{\mathrm{sim}},A{6}_{\mathrm{sim}}\right\} $$

Users of 3DeFDR-5C may choose whichever condition they like when generating the null replicate set.

The simulated interaction counts ${C}_{t,r,i,j}^{\prime \mathrm{sim}}$ were then subjected to the matrix balancing, binning, modeling, and *p* value transformation steps described above. 3DeFDR-5C takes as input the simulated replicate interaction scores ${\mathrm{IS}}_{t_s,r}^{\mathrm{sim}}$ and experimental replicate interaction scores ${\mathrm{IS}}_{t_s,r}$ for each region *r*, replicate *s*, and condition *t*.

#### Identification of the background interaction set

Prior to the identification of differential looping interactions, we created a background null interaction set consisting of all interactions for which the interaction scores ${\mathrm{IS}}_{t_s,r,k,l}$ of all replicates of every condition were less than a background threshold *b* as in Equation 19:

19 $$ \mathrm{Background}\ \mathrm{loops}=\left\{\left(r,k,l\right):\underset{t_s\in S}{\max}\left({\mathrm{IS}}_{t_s,r,k,l}\right)<b\right\} $$

The exact threshold for background interactions that we used was *b* =  − 10 × log_2_(0.8), corresponding to a *p* value threshold of 0.8. Interactions not placed in this set were then passed on for further analysis for differential looping in the 3DeFDR-5C pipeline.

#### Preliminary classification of differential looping interactions

As outlined in Additional file 2: Fig. S1, to ultimately be classified as differential, a loop must pass thresholds for both baseline significance and IS difference across conditions.

#### Baseline significance filtering

To meet the criteria for differential looping for any differential classification *h*, an interaction must have ${\mathrm{IS}}_{t_s,r,k,l}$ greater than a specific significance threshold *g* for all replicates in at least one condition in *T* as in Additional file 2: Fig. S1D and Equation 20:

20 $$ \mathrm{Significant}\ \mathrm{loops}=\left\{\left(r,k,l\right):\underset{t}{\max}\left[\underset{s}{\min}\left({\mathrm{IS}}_{t_s,r,k,l}\right)\right]>g\right\} $$

The threshold for a significant looping interaction used in the results presented in the main figures was *g* =  − 10 × log_2_(0.165), corresponding to a *p* value threshold of 0.165.

#### Thresholding interaction score differences across conditions

Starting with the subset of significant loops across conditions, we then set out to classify interactions according to how much their interaction scores changed across cellular conditions (Additional file 2: Fig. S1E and Additional file 3: Table S2). For each interaction (*r*, *k*, *l*) in the set of significant loops (Equation 20), we computed the difference in interaction score between each possible pair of replicates belonging to different conditions. We then computed initial looping class assignments (Equation 3) across a sweep of IS difference thresholds *d* as shown in Additional file 3: Table S2. Additionally, in Additional file 3: Table S2, we provide the exact set of thresholds applied to obtain each possible looping classification of a bin-bin pair in dataset capturing three conditions.

In 3DeFDR-5C, loop classifications are determined using this thresholding approach for each difference threshold across a sweep of all possible difference thresholds in a given data set. These classifications are considered preliminary prior to the application of the eFDR control procedure described in the next section.

#### Final loop classification via an adaptive eFDR control procedure

After obtaining preliminary classifications of each interaction across a sweep of IS difference thresholds, we determined each ${\mathrm{IS}}_{t_s,r,k,l}$ interaction’s final classification via the application of a classification-specific eFDR control procedure. For each possible loop classification *h* ∈ *H*, we computed its eFDR for every tested difference threshold *d*, acquiring a difference threshold-to-eFDR mapping for each class, eFDR_*d*, *h*_, as in Equation 5. We next applied the eFDR threshold *τ* to this mapping, identifying the difference threshold *d* at which eFDR_*d*, *h*_ is closest to but still less than *τ*, and report loop calls of class *h* at this distance threshold. We perform the eFDR controlling procedure for every differential looping class *h* ∈ *H*, and the combined set of loop calls for each class constitutes our final set of differential classified loops.

Additionally, eFDR estimates can be computed as an average over a user-specified number, *N*_null − sets_, of null replicate sets as in Equation 21:

21 $$ {\mathrm{eFDR}}_{d,h}=\frac{\frac{1}{N_{\mathrm{null}-\mathrm{sets}}}{\sum}_{m=1}^{N_{\mathrm{null}-\mathrm{sets}}}\operatorname{card}\left(\left\{\left(r,k,l\right)\in {h}_{\mathrm{null}}^d\ \right\}\right)\ }{\operatorname{card}\left(\left\{\left(r,k,l\right)\in {h}_{\mathrm{exp}}^d\ \right\}\right)} $$

The numerator is now the average number of loops called as class *h* in the null data sets at difference threshold *d*. The approach in Equation 21 can reduce variability in eFDR estimates due to random differences between different simulation sets generated from the same counts-generating model.

### Benchmarking 3DeFDR on 5C

To benchmark the effectiveness of 3DeFDR-5C for loop calling, we implemented two additional methods for classifying differential looping interactions: (1) we applied conventional ANOVA and (2) we formulated a new likelihood ratio test, 3DLRT. Using these methods, we assigned a differential looping (DL) *p* value to every interaction in an experimental dataset. In both approaches, output *p* values were then corrected for multiple testing using the Benjamini-Hochberg step-up procedure for controlling FDR. Differential looping classifications were ultimately assigned using ANOVA and 3DLRT as detailed below and described in Additional file 2: Fig. S3.

#### ANOVA

As a basic benchmark to compare to our more sophisticated methods, we applied ANOVA directly to either the interaction scores ${\mathrm{IS}}_{t_s,r,k,l}$ or the *z*-scores ${Z}_{t_s,r,k,l}$ of the experimental replicate set. To account for the large number of interactions tested, we corrected the resulting differential interaction *p* values for multiple testing by applying the Benjamini-Hochberg (BH) step-up procedure. The resulting BH-FDR adjusted *p* values were then thresholded according to a user-defined FDR to determine which interactions are significantly differential.

It should be noted that ANOVA may not be particularly appropriate when applied to the experimental design described in this paper. The interaction scores ${\mathrm{IS}}_{t_s,r,k,l}$ are not normally distributed, whereas ANOVA assumes that the data are normally distributed. Unlike the interaction scores, the *z*-scores ${Z}_{t_s,r,k,l}$ are normally distributed with unit variance under the null hypothesis of the statistical model we use to call loops. Despite this, ANOVA attempts to independently re-estimate variance parameters for every ${\mathrm{IS}}_{t_s,r,k,l}$ or ${Z}_{t_s,r,k,l}$ interaction tested without sharing information across interactions.

#### 3DLRT

As an alternative method for identifying statistically significant differential interactions, we developed a new likelihood ratio test (3DLRT). 3DLRT compares the likelihood of the data assuming that the loop is not differentially interacting (null model) to the likelihood of the data under the assumption that the loop is differential (alternative model). We parameterized each model with parameters that best matched the underlying data under the constraints of that model. If the likelihood of the observed data under the alternative model was significantly higher than that under the null model, then we rejected the null hypothesis that the loop is not differential between conditions.

We formulated 3DLRT using either the *z*-scores ${Z}_{t_s,r,k,l}$ (3DLRT-Z) or the interaction scores ${\mathrm{IS}}_{t_s,r,k,l}$ (3DLRT-IS) of the experimental replicate set. The derivation of 3DLRT that uses IS as input (3DLRT-IS) is provided in additional supplementary discussion below. In the 3DLRT-Z model, the *z*-scores are assumed to follow a unit-variance normal probability density function with a single looping effect size or shift parameter *μ* (Equation 22):

22 $$ {f}_Z\left(Z;\mu \right)=\frac{1}{\sqrt{2\pi }}{e}^{-\frac{{\left(Z-\mu \right)}^2}{2}}\kern0.5em $$

The test statistic for the likelihood ratio test based on the *z*-scores is shown as Equation 23:

23 $$ {T}_{\mathrm{LRT}}=2\log \frac{\underset{{\hat{\mu}}_A,{\hat{\mu}}_B,{\hat{\mu}}_C}{\max}\left[{\prod}_{t\in T,s\in S}{f}_Z\left({Z}_{t_s};{\hat{\mu}}_t\right)\right]}{\underset{{\hat{\mu}}_0}{\max}\left[{\prod}_{t\in T,s\in S}{f}_Z\left({Z}_{t_s};{\hat{\mu}}_0\right)\right]} $$

where under the null hypothesis *T*_LRT_ should be asymptotically chi-square distributed with two degrees of freedom. The intuition behind our formulation of 3DLRT is included below in the “Detailed description of the 3DLRT-Z test” section.

Finally, we assessed the significance of the test statistic by comparing it to the chi-square distribution with degrees of freedom equal to the difference in the number of free parameters in our two models. In our case, the alternative hypothesis model has three parameters and the null hypothesis model has one, so we have two degrees of freedom. We then adjusted chi-square *p* values for multiple testing by applying the Benjamini-Hochberg step-up procedure. The resulting BH-FDR adjusted *p* values were then thresholded according to the user-defined target FDR to determine which interactions are significantly differential. Significantly differential interactions were then assigned to differential looping classes using the same logic as that used by 3DeFDR-5C (Additional file 2: Fig. S3 and Additional file 3: Table S2).

### Detailed description of the 3DLRT-Z test

#### Set-up and assumptions

The test statistic for 3DLRT-Z is shown in Equation 24:

24 $$ {T}_{\mathrm{LRT}}=2\log \frac{\prod_if\left({z}_i;{\hat{\mu}}_{1i}\right)}{\prod_if\left({z}_i;{\hat{\mu}}_{0i}\right)} $$

where *f*(*x*; *μ*) is the probability density function for the normal distribution, constrained to unit variance and parameterized with a single looping effect size or shift parameter *μ*, as specified in (Equations 22–23), ${\hat{\mu}}_{0i}$ is the appropriate shift parameter estimate for *z*_*i*_ under the null hypothesis, and ${\hat{\mu}}_{1i}$ is the appropriate shift parameter estimate for *z*_*i*_ under the alternate hypothesis.

The parameter estimates ${\overset{\rightharpoonup }{\hat{\mu}}}_0$ and ${\overset{\rightharpoonup }{\hat{\mu}}}_1$ are constrained by the specific choice of null and alternate hypotheses. These parameters are chosen to maximize the likelihood of the data under the null (Equation 25) and alternate hypothesis (Equation 26), respectively:

25 $$ {\overset{\rightharpoonup }{\hat{\mu}}}_0=\underset{{\overset{\rightharpoonup }{\mu}}_0}{\mathrm{argmax}}{\prod}_if\left({z}_i;{\mu}_{0i}\right),\mathrm{subject}\ \mathrm{to}\ \mathrm{null}\ \mathrm{hypothesis}\ \mathrm{constraints} $$

26 $$ {\overset{\rightharpoonup }{\hat{\mu}}}_1=\underset{{\overset{\rightharpoonup }{\mu}}_1}{\mathrm{argmax}}{\prod}_if\left({z}_i;{\mu}_{1i}\right),\mathrm{subject}\ \mathrm{to}\ \mathrm{alternate}\ \mathrm{hypothesis}\ \mathrm{constraints} $$

Applying this choice of ${\overset{\rightharpoonup }{\hat{\mu}}}_0$ and ${\overset{\rightharpoonup }{\hat{\mu}}}_1$ to the equation for the likelihood ratio test statistic *T* above, we derive Equation 27:

27 $$ T=2\log \frac{\underset{{\overset{\rightharpoonup }{\mu}}_1}{\max }{\prod}_if\left({z}_i;{\mu}_{1i}\right)}{\underset{{\overset{\rightharpoonup }{\mu}}_0}{\max }{\prod}_if\left({z}_i;{\mu}_{0i}\right)} $$

Equation 27 can also be written as Equation 28:

28 $$ T=2\log \frac{\underset{{\overset{\rightharpoonup }{\mu}}_1}{\max }L\left(\overset{\rightharpoonup }{z}\ |\ {\overset{\rightharpoonup }{\mu}}_1\right)}{\underset{{\overset{\rightharpoonup }{\mu}}_0}{\max }L\left(\overset{\rightharpoonup }{z}\ |\ {\overset{\rightharpoonup }{\mu}}_0\right)} $$

where $L\left(\overset{\rightharpoonup }{z}\ |\ {\overset{\rightharpoonup }{\mu}}_1\right)$ is the total likelihood of the data $\overset{\rightharpoonup }{z}$ given a mean parameter vector ${\overset{\rightharpoonup }{\mu}}_1$ under the alternate hypothesis (Equation 29):

29 $$ L\left(\overset{\rightharpoonup }{z}\ |\ {\overset{\rightharpoonup }{\mu}}_1\right)={\prod}_if\left({z}_i;{\mu}_{1i}\right) $$

and $L\left(\overset{\rightharpoonup }{z}\ |\ {\overset{\rightharpoonup }{\mu}}_0\right)$ is the total likelihood of the data $\overset{\rightharpoonup }{z}$ given a mean parameter vector ${\overset{\rightharpoonup }{\mu}}_0$ under the null hypothesis (Equation 30):

30 $$ L\left(\overset{\rightharpoonup }{z}\ |\ {\overset{\rightharpoonup }{\mu}}_0\right)={\prod}_if\left({z}_i;{\mu}_{0i}\right) $$

The key difference between the two likelihood functions is that the constraints on ${\overset{\rightharpoonup }{\mu}}_1$ and ${\overset{\rightharpoonup }{\mu}}_0$ may be different, as dictated by the alternate and null hypotheses, respectively.

The likelihood ratio test statistic *T*_LRT_ should be asymptotically chi-square distributed under the null hypothesis. The degrees of freedom in the chi-square distribution depend on the constraints imposed by the null and alternate hypotheses. For example, in our 3 conditions, 2 replicate per condition experimental design, the parameters include (Equations 31 and 32):

31 $$ {\overset{\rightharpoonup }{\hat{\mu}}}_1=\left[{\hat{\mu}}_A,{\hat{\mu}}_A,{\hat{\mu}}_B,{\hat{\mu}}_B,{\hat{\mu}}_C,{\hat{\mu}}_C\right] $$

32 $$ {\overset{\rightharpoonup }{\hat{\mu}}}_0=\left[{\hat{\mu}}_{ABC},{\hat{\mu}}_{ABC},{\hat{\mu}}_{ABC},{\hat{\mu}}_{ABC},{\hat{\mu}}_{ABC},{\hat{\mu}}_{ABC}\right] $$

In this case, the chi-square distribution will have two degrees of freedom, since the alternate hypothesis has three free parameters and the null hypothesis has one free parameter. More specifically, our null hypothesis is Equation 33:

33 $$ {\mu}_A={\mu}_B,{\mu}_A={\mu}_C,{\mu}_B={\mu}_C $$

and our alternate hypothesis is Equation 34:

34 $$ {\mu}_A\ne {\mu}_B\ \mathrm{OR}\ {\mu}_A\ne {\mu}_C\ \mathrm{OR}\ {\mu}_B\ne {\mu}_C $$

The optimal estimate of the single shift parameter for the null hypothesis is Equation 35:

35 $$ {\hat{\mu}}_0=\underset{\mu }{\mathrm{argmax}}{\prod}_if\left({x}_i;\mu \right)={\hat{\mu}}_{ABC} $$

The optimal estimates of the shift parameters for the alternate hypothesis are shown in Equation 36:

36 $$ {\hat{\mu}}_{1i}=\Big\{{\displaystyle \begin{array}{cc}\underset{\mu }{\mathrm{argmax}}{\prod}_{i\in A}f\left({x}_i;\mu \right)={\hat{\mu}}_A&, \forall i\in A\\ {}\underset{\mu }{\mathrm{argmax}}{\prod}_{i\in B}f\left({x}_i;\mu \right)={\hat{\mu}}_B&, \forall i\in B\\ {}\underset{\mu }{\mathrm{argmax}}{\prod}_{i\in C}f\left({x}_i;\mu \right)={\hat{\mu}}_C&, \forall i\in C\end{array}}\operatorname{} $$

For all six replicates in our three-condition experiment, the 3DLRT test statistic is then Equation 37:

37 $$ {T}_{\mathrm{LRT}}=2\log \frac{f\left({z}_{A1};{\hat{\mu}}_A\right)\times f\left({z}_{A2};{\hat{\mu}}_A\right)\times f\left({z}_{B1};{\hat{\mu}}_B\right)\times f\left({z}_{B2};{\hat{\mu}}_B\right)\times f\left({z}_{C1};{\hat{\mu}}_C\right)\times f\left({z}_{C2};{\hat{\mu}}_C\right)}{f\left({z}_{A1};{\hat{\mu}}_{AB C}\right)\times f\left({z}_2;{\hat{\mu}}_{AB C}\right)\times f\left({z}_{B1};{\hat{\mu}}_{AB C}\right)\times f\left({z}_{B2};{\hat{\mu}}_{AB\mathrm{C}}\right)\times f\left({z}_{C1};{\hat{\mu}}_{AB C}\right)\times f\left({z}_{C2};{\hat{\mu}}_{AB C}\right)} $$

which can be rewritten more compactly to obtain Equation 23.

#### 3DLRT-Z *p* value assignment and interaction classification

We call a single *p* value *P*_LRT_ for each bin-bin pair being tested for differential interaction strength from the likelihood ratio test statistic *T*_LRT_ using a chi-square distribution with two degrees of freedom. If there are *N* bin-bin pairs being tested for differential interaction strength, there are *N* of these *p* values. We perform Benjamini-Hochberg multiple testing correction across all *N* of these *p* values, obtaining adjusted *p* values *Q*_LRT_. Interactions whose *Q*_LRT_ is above the target false discovery rate are called “constitutive.” Interactions whose *Q*_LRT_ is below the target false discovery rate are assigned a differential interaction classification category by first ranking the $$ \hat{\mu} $$ values across the three conditions so that $$ {\hat{\mu}}_{A^{\prime }}>{\hat{\mu}}_{B^{\prime }}>{\hat{\mu}}_{C^{\prime }} $$ (where $$ A^{\prime } $$, $$ B^{\prime } $$, $$ C^{\prime } $$ represent a permutation of the original conditions *A*, *B*, *C*), and then deciding between the $$ A^{\prime } $$ only and $$ A^{\prime } $$$$ B^{\prime } $$ classification categories by checking to see which of the pairs ($$ A^{\prime } $$, $$ B^{\prime } $$) or ($$ A^{\prime } $$, $$ B^{\prime } $$) have $$ \hat{\mu} $$ values closer together. For example, if the pairwise comparisons have a low enough *Q*_LRT_ to pass the FDR threshold and also follow $$ {\hat{\mu}}_{A^{\prime }}>{\hat{\mu}}_{B^{\prime }}>{\hat{\mu}}_{C^{\prime }} $$, then the classification category is assigned to be (Equation 38):

38 $$ \mathrm{Classification}\ \mathrm{category}=\left\{\begin{array}{cl}{A}^{\prime}\mathrm{only}&, \mid {\hat{\mu}}_{A^{\prime }}-{\hat{\mu}}_{B^{\prime }}\mid >\mid {\hat{\mu}}_{B^{\prime }}-{\hat{\mu}}_{C^{\prime }}\mid \\ {}{A}^{\prime }{B}^{\prime }&, \mid {\hat{\mu}}_{A^{\prime }}-{\hat{\mu}}_{B^{\prime }}\mid <\mid {\hat{\mu}}_{B^{\prime }}-{\hat{\mu}}_{C^{\prime }}\mid \end{array}\right) $$

The full series of logic statements for all looping classifications are shown in Additional file 4: Fig. S3.

### 3DeFDR-HiC

#### Overview

3DeFDR-HiC was developed to allow application of 3DeFDR to Hi-C data by addressing the key ways in which Hi-C data differs from 5C data. First, Hi-C contact matrices are significantly larger than 5C contact matrices. The large size of the data makes it infeasible to generate large numbers of simulated replicates needed for estimation of the null distribution of the 3DeFDR-5C test statistic. Second, Hi-C datasets are typically analyzed using a different set of transformations than those used for 5C data. Most notably, Hi-C read counts are typically summed within pairs of non-overlapping genomic bins, allowing easy interpretation of the sum of raw read counts in each bin-bin pair as a discrete independent random variable. Modeling of discrete random variables by Poisson or negative binomial (NB) distributions permits the formulation of a dramatically simplified statistical model whose FDR can be determined directly from analytically defined null distributions fitted directly to the real data rather than empirical distributions estimated via complex transformations and computationally expensive simulations. In the following sections, we describe the simplified statistical model that makes up 3DeFDR-HiC, how we estimated this model’s parameters, and how we used this model to test for differential loops.

#### Statistical model of Hi-C counts

We described binned Hi-C read counts using the negative binomial distribution, parameterized in terms of its mean *μ* and dispersion *α*, whose probability mass function is (Equation 39):

39 $$ f\left(x;\mu, \alpha \right)=\frac{\Gamma \left({\alpha}^{-1}+x\right)}{x!\Gamma \left({\alpha}^{-1}\right)}{\left(\frac{\alpha^{-1}}{\alpha^{-1}+\mu}\right)}^{\alpha^{-1}}{\left(\frac{\mu }{\alpha^{-1}+\mu}\right)}^x $$

We modeled the read count for the interaction between the *i*th and *j*th bin on a given chromosome in replicate *r* ∈ *R* (where *R* is the set of all replicates being analyzed) in condition *c*(*r*) (the biological condition of replicate *r*) as a negative binomial random variable *X*_*r*, *i*, *j*_ (Equation 40):

40 $$ {X}_{r,i,j}\sim \mathrm{NB}\left({\mu}_{c(r),i,j}{b}_{r,i}{b}_{r,j}{s}_{r,j-i}\kern0.5em ,\kern0.5em {\alpha}_{c(r),j-i}\right) $$

where *μ*_*c*(*r*),*i*,*j*_ represents a condition-specific, replicate-independent true interaction strength for the interaction between the *i*th and *j*th bin in condition *c*(*r*), *b*_*r*,*i*_ and *b*_*r*,*j*_ represent a locus-specific bias factor for the *i*th and *j*th bin, respectively, *s*_*r*,*j* − *i*_ represents a distance-dependent library size factor for replicate *r* at distance *j* − *i*, *α*_*c*(*r*),*j* − *i*_ represents a condition-specific, distance-dependent dispersion value for condition *c*(*r*) at distance *j* − *i*, and NB(*μ*, *α*) represents a negative binomial distribution with mean μ and dispersion α. Distance *j* − *i* is in units of the number of bins.

The replicate-specific bias vectors *b* are intended to capture the effects of locus-specific biases such as GC content, restriction fragment length, and mappability which are known to influence Hi-C read counts [58, 59]. The replicate-specific, distance-dependent library size factors *s*_*r*,*j* − *i*_ are intended to capture the effects of different sequencing depths across the replicates being analyzed, which often influence different distance scales in an inconsistent, nonlinear manner. The probability mass function of our final model is shown in Equation 41:

41 $$ {\displaystyle \begin{array}{rl}f\left({x}_{r,i,\mathrm{j}}\kern0.5em ;\kern0.5em {\mu}_{c(r),i,j}{b}_{r,i}{b}_{r,j}{s}_{r,j-i}\kern0.5em ,\kern0.5em {\alpha}_{c(r),j-i}\right)& =\frac{\Gamma \left({\alpha_{c(r),j-i}}^{-1}+{x}_{r,i,j}\right)}{x_{r,i,j}!\Gamma \left({\alpha_{c(r),j-i}}^{-1}\right)}\\ {}& \times {\left(\frac{{\alpha_{c(r),j-i}}^{-1}}{{\alpha_{c(r),j-i}}^{-1}+{\mu}_{c(r),i,j}{b}_{r,i}{b}_{r,j}{s}_{r,j-i}}\right)}^{\alpha^{-1}}\\ {}& \times {\left(\frac{\mu_{c(r),i,j}{b}_{r,i}{b}_{r,j}{s}_{r,j-i}}{{\alpha_{c(r),j-i}}^{-1}+{\mu}_{c(r),i,j}{b}_{r,i}{b}_{r,j}{s}_{r,j-i}}\right)}^{x_{r,i,j}}\end{array}} $$

#### Estimating bias vectors

We estimated ${\hat{b}}_{r,:}$, the locus-specific bias vector for each replicate *r*, using the Knight-Ruiz matrix balancing algorithm [11, 59]. In order to facilitate convergence of the algorithm, we filtered out sparse rows and columns of the contact matrix. Specifically, we filtered out any row of the contact matrix with fewer than 25 nonzero entries within the first 300 entries counting from the diagonal in either direction (upstream or downstream). After matrix balancing, we filtered out rows and columns with bias factors above 10 or below 0.1, as we and others have observed that these rows and columns are likely to contain artifacts from the balancing procedure [66].

#### Estimating size factors

We found that a single median of ratios size factor [67] was insufficient to normalize the data across libraries with different sequencing depths, leading to biases visible in MA plots of the data. Therefore, we chose to estimate ${\hat{s}}_{r,d}$, the per-distance size factors for replicate *r* at distance *d*, using a distance-dependent variation of the median of ratios method. To reduce the variance in our size factor estimates, we first grouped all bin-bin pairs on each chromosome with a non-zero read count in any replicate and with interaction distances less than 5 Mb into 100 equal-number groups by distance, creating sets of bin-bin pair indices *G*_*k*_ = {(*i*, *j*) ∣ pixel (*i*, *j*) is in the *k*th group}. We then computed a per-group median of ratios size factor $\tilde{s}_{r,k}$ for each group *G*_*k*_ for each replicate *r* ∈ *R* (Equation 42):

42 $$ \tilde{s}_{r,k}=\underset{\left(i,j\right)\in {G}_k}{\mathrm{median}}\frac{y_{r,i,j}}{{\left(\prod \limits_{r^{\prime}\in R}{y}_{r^{\prime },i,j}\right)}^{\frac{1}{\mid R\mid }}} $$

where ${y}_{r,i,j}=\frac{x_{r,i,j}}{{\hat{b}}_{r,i}{\hat{b}}_{r,j}}$ represents the matrix balanced read count for the interaction between the *i*th and *j*th bin in replicate *r*. We excluded points (*i*, *j*) that have a zero value in any replicate from further analysis. To ensure that our final size factor estimates are a smooth function of interaction distance, we then used piecewise linear interpolation on the graph of our per-group size factors $\tilde{s}_{r,k}$ versus their per-group average distance $\underset{\left(i,j\right)\in {G}_k}{\mathrm{mean}}\left(j-i\right)$ to obtain per-distance size factors ${\hat{s}}_{r,d}$ for all interaction distances *d* between 0 and 5 Mb (0 ≤ *d* ≤ 500) and for all replicates *r* ∈ *R*. We excluded interactions beyond 5 Mb (*d* > 500) from further analysis, because the vast majority of true looping interactions detectable by Hi-C are thought to occur within this distance. We found that this distance-dependent size factor-based normalization was sufficient to equalize library size differences across length scales (Additional file 2: Fig. S14).

#### Estimating the dispersion

To estimate dispersion parameters using only a small number of replicates, we took advantage of the large number of pixels in Hi-C datasets. Similar to the underlying assumption in 3DeFDR-5C, we assumed that binned Hi-C read counts at similar distance scales should have similar statistical properties. Therefore, we pooled interactions with identical interaction distances to obtain less noisy estimates of the dispersion parameter. To account for the dependence between dispersion and mean commonly observed in high-throughput sequencing datasets [67], we pooled together interactions with the same interaction distance (and therefore similar means). We estimated a condition-specific distance-dispersion relationship (DDR), where the dispersion for an interaction can be estimated from its condition and the linear genomic distance separating the two interacting bins.

To estimate the DDR for a given condition *c*, we first computed the mean across replicates of the same condition for every pixel in Hi-C matrices of bias- and size factor-normalized data (Equation 43):

43 $$ {\overline{z}}_{c,i,j}=\frac{1}{\mid {R}_c\mid}\sum \limits_{r\in {R}_c}{z}_{r,i,j} $$

where *R*_*c*_ = {*r* ∈ *R* ∣ *c*(*r*) = *c*} represents the set of replicates in condition *c* and ${z}_{r,i,j}=\frac{x_{r,i,j}}{{\hat{b}}_{r,i}{\hat{b}}_{r,j}{\hat{s}}_{r,j-i}}$ represents the interaction count for the interaction between the *i*th and *j*th bin in replicate *r*, normalized for locus-specific bias and library size. During our exploratory analysis, we learned that we are underpowered to answer statistical questions about possible looping interactions at pixels with < 1 read in a given condition. Therefore, we eliminated points (*i*, *j*) which have a normalized mean for a given pixel, ${\overline{z}}_{c,i,j}$, below 1 for replicates across any condition *c*. For each condition *c*, we grouped the surviving points (*i*, *j*) according to their interaction distance *j* − *i*. At each interaction distance *d*, and for each condition *c*, we estimated a single dispersion value ${\overset{\sim }{\alpha}}_{c,d}$ across the normalized interaction counts *z*_*r*,*i*,*j*_ for all points (*i*, *j*) ∈ {(*i*, *j*) ∣ *j* − *i* = *d*} and all replicates *r* ∈ *R*_*c*_ using an implementation of quantile-adjusted conditional maximum likelihood (qCML) [68]. We tried simpler dispersion estimation approaches, but qCML provided the most unbiased estimates during our exploratory data analysis. Briefly, we started from an initial dispersion guess of 0.01 and iteratively update it by repeating two steps. In the first step, we used a “quantile-to-quantile mapping” method based on the q2qnbinom() function in EdgeR to transform *x*_*r*,*i*,*j*_ into pseudocount $\tilde{x}_{r,i,j}$ on the scale of the geometric mean of the combined scaling factors ${\hat{b}}_{r,i}{\hat{b}}_{r,j}{\hat{s}}_{r,j-i}$ for each pixel across replicates of the same condition. To determine the means of the negative binomial distributions we were converting between, we fitted the mean parameter *μ*_*c*(*r*),*i*,*j*_ of our negative binomial model in Equation 41 for each pixel directly via maximum likelihood using the raw data, our combined per-pixel scaling factors ${\hat{b}}_{r,i}{\hat{b}}_{r,j}{\hat{s}}_{r,j-i}$, and our latest estimate of the dispersion ${\overset{\sim }{\alpha}}_{c,d}$. In the second step, we updated our guess for ${\overset{\sim }{\alpha}}_{c,d}$ by optimizing the conditional maximum likelihood of the pseudodata (Equation 44):

44 $$ {\displaystyle \begin{array}{rl}{\overset{\sim }{\alpha}}_{c,d}=\underset{\alpha }{\mathrm{argmax}}\sum \limits_{\left(i,j\right)\mid j-i=d}\Big[& \sum \limits_{r\in {R}_c}\mathrm{log}\Gamma \left(\tilde{x}_{r,i,j}+{\alpha}^{-1}\right)+\mathrm{log}\Gamma \left(|{R}_c|\times {\alpha}^{-1}\right)\\ {}& \kern1em -\mathrm{log}\Gamma \left(\sum \limits_{r\in {R}_c}\tilde{x}_{r,i,j}+|{R}_c|\times {\alpha}^{-1}\right)-\mid {R}_c\mid \times \mathrm{log}\Gamma \left({\alpha}^{-1}\right)\Big]\end{array}} $$

We repeated these steps until the absolute change in the estimated dispersion ${\overset{\sim }{\alpha}}_{c,d}$ was less than 1e−4. Unlike all other steps in our workflow which were performed independently on each chromosome, we estimated a single value of ${\overset{\sim }{\alpha}}_{c,d}$ for each condition and each distance scale using data from all chromosomes. We excluded distance scales lower than 40 kb (*d* < 4) since we found these distance scales to be difficult to model and call loops in even within individual conditions. We also filtered our per-condition loop call sets to exclude loops within 40 kb to help avoid false-positive calls at extremely short distances. Future work may extend our model to include shorter distance scales.

#### Fitting distance versus dispersion trends

We observed that the per-distance dispersion estimates ${\overset{\sim }{\alpha}}_{c,d}$ showed a generally clear and consistent trend with respect to distance, though the estimates appeared to get noisy at high distance scales where fewer bin-bin pairs passed our minimum-mean filter. Therefore, we applied LOWESS smoothing separately for each condition *c* to the graph of interaction distance *d* versus ${\overset{\sim }{\alpha}}_{c,d}$, obtaining final smoothed per-condition, per-distance dispersion estimates ${\hat{\alpha}}_{c,d}.$ We observed that the per-distance dispersion estimates ${\overset{\sim }{\alpha}}_{c,d}$ showed low sampling variability at short distances, but high sampling variability at long distances. Therefore, when performing this LOWESS fit, we decided to employ a weighted LOWESS fitting strategy as detailed below.

We started by estimating the precision of each per-distance dispersion estimate ${\overset{\sim }{\alpha}}_{c,d}$ via a rolling sample variance with a centered window of size 20 distance scales *d*. When the rolling window rolled off the left or right edge of ${\overset{\sim }{\alpha}}_{c,:}$, we filled it with the first or highest sample variance, respectively. In this way, we obtained an estimate of the sampling variance of ${\overset{\sim }{\alpha}}_{c,d}$ for each distance *d*, which we denote as *v*_*c*,*d*_ (Equation 45):

45 $$ {v}_{c,d}=\left\{\begin{array}{ccc}\mathrm{undefined}&, & d<4\kern0.5em \left(\mathrm{these}\ \mathrm{distance}\ \mathrm{ranges}\ \mathrm{were}\ \mathrm{excluded}\right)\\ {}{v}_{c,14}&, & 4\le d<14\kern0.5em \left(\mathrm{window}\ \mathrm{rolls}\ \mathrm{beyond}\ \mathrm{left}\ \mathrm{edge}\right)\\ {}\frac{\sum \limits_{d^{\prime }=d-10}^{d+9}{\left({\overset{\sim }{\alpha}}_{c,{d}^{\prime }}-\frac{\sum \limits_{d^{{\prime\prime} }=d-10}^{d+9}{\overset{\sim }{\alpha}}_{c,{d}^{\prime }}}{20}\right)}^2}{19}&, & 14\le d\le 491\left(\mathrm{rolling}\ \mathrm{window}\ \mathrm{variance}\right)\\ {}\underset{14\le {d}^{\prime}\le 491}{\max}\left({v}_{c,{d}^{\prime }}\right)&, & 491<d\le 500\kern0.5em \left(\mathrm{window}\ \mathrm{rolls}\ \mathrm{beyond}\ \mathrm{right}\ \mathrm{edge}\right)\\ {}\mathrm{undefined}&, & d>500\kern0.5em \left(\mathrm{these}\ \mathrm{distance}\ \mathrm{ranges}\ \mathrm{were}\ \mathrm{excluded}\right)\end{array}\right) $$

We then computed unscaled weights *w*_*c*,*d*_ as the respective precisions (inverse variances) raised to the 1/4th power (Equation 46):

46 $$ {w}_{c,d}={\left(\frac{1}{v_{c,d}}\right)}^{\frac{1}{4}} $$

We found empirically that this choice of transformation resulted in weights that yielded LOWESS fits robust to changes in the LOWESS fraction parameter across all distance scales.

To weigh the LOWESS fit towards the higher-precision points, we rescaled the unscaled weights *w*_*c*,*d*_ so that the lowest was equal to 1, rounded them to the closest non-greater integer, and created a number of duplicates of each point equal to this integer $\tilde{w}_{c,d}$ (Equation 47):

47 $$ \tilde{w}_{c,d}=\left\lfloor \frac{w_{c,d}}{\underset{d^{\prime }}{\min}\left({w}_{c,d}^{\prime}\right)}\right\rfloor $$

Finally, we performed LOWESS fitting on the duplicated data points using a LOWESS smoothing fraction of $\frac{15}{\underset{d}{\max}\left(\tilde{w}_{c,d}\right)\times \underset{d}{\mathrm{mean}}\left({w}_{c,d}\right)}$. We empirically found this LOWESS smoothing fraction to yield reasonable LOWESS fits for a wide range of dataset sizes (number of chromosomes) and maximum distance scales. The optimal choice of LOWESS smoothing fraction remains an open research question, and this value can be overridden by the user with a simple keyword argument. Thus, we obtained a smooth dispersion-distance relationship function *g*(*d*) which returns the smoothed dispersion given a distance *d*.

We observed that dispersion decreased with increasing distance at extremely short distance scales (typically within ~ 70 kb). To avoid underestimation of dispersion at these distance scales by the LOWESS fit, we used the per-distance dispersion estimates ${\overset{\sim }{\alpha}}_{c,d}$ as our final dispersion estimates ${\hat{\alpha}}_{c,d}$ for the first few distances *d* until these dispersion estimates began increasing, switching to the LOWESS fit for all remaining distances (Equation 48):

48 $$ {\hat{\alpha}}_{c,d}=\left\{\begin{array}{ccc}{\overset{\sim }{\alpha}}_{c,d}&, & d<{d}_c^{\ast}\\ {}g(d)&, & d\ge {d}_c^{\ast}\end{array}\right) $$

where *g*(*d*) is the smooth dispersion-relationship function we obtained via our weighted LOWESS fitting and ${d}_c^{\ast }$ is the first distance (lowest *d*) for which ${\overset{\sim }{\alpha}}_{c,{d}^{\ast }}>{\overset{\sim }{\alpha}}_{c,{d}^{\ast }-1}$ (Equation 49):

49 $$ {d}_c^{\ast }=\underset{d\in \left\{{d}^{\prime}\mid {\overset{\sim }{\alpha}}_{c,{d}^{\prime }}>{\overset{\sim }{\alpha}}_{c,{d}^{\prime }-1}\right\}}{\min }(d) $$

We believe that using the per-distance dispersion estimates ${\overset{\sim }{\alpha}}_{c,d}$ at the shortest distances scales is acceptable because we observed that the sampling variability of ${\overset{\sim }{\alpha}}_{c,d}$ was lowest at the shortest distance scales.

#### Likelihood ratio test for differential loops

Having fitted almost all the parameters of our statistical model, we then tested for differences in the last parameter, the true condition-specific interaction strength *μ*_*c*, *i*, *j*_. Our null hypothesis (for each tested interaction) was that the true interaction strength is the same in each condition, *μ*_*c*′,*i*,*j*_ = *μ*_*c*′′,*i*,*j*_  ∀  *c*′, *c*′′ ∈ *C*, where *C* is the set of all conditions. Our alternative hypothesis was that any pair of conditions differ in their true interaction strength (i.e., ∃ *c*′, *c*′′ ∈ *C* such that *μ*_*c*′,*i*,*j*_ ≠ *μ*_*c*′′,*i*,*j*_). To test these hypotheses, we constructed models corresponding to each hypothesis and compared their likelihoods using a likelihood ratio test. Under the null hypothesis model, we computed a single true interaction strength parameter *μ*_0,*i*,*j*_, representing the constraint that all the condition-specific true interaction strength parameters must be equal (i.e., *μ*_0,*i*,*j*_ = *μ*_*c*,*i*,*j*_  ∀  *c* ∈ *C*). Under the alternative hypothesis model, we allowed each condition to have its own condition-specific interaction strength parameter *μ*_*c*,*i*,*j*_. For each model, we fitted the true interaction strength parameter(s) using maximum likelihood estimation and Equation 39, holding all other model parameters fixed, to obtain estimates ${\hat{\mu}}_{0,i,j}$ and ${\hat{\mu}}_{c,i,j}$ (for each condition *c*) for the null and alternative hypothesis models, respectively. We then constructed the likelihood ratio for each pixel (*i*, *j*) (Equation 50):

50 $$ {\lambda}_{i,j}=\frac{\prod \limits_{c\in C}\prod \limits_{r\in {R}_c}f\left({x}_{r,i,j};{\hat{\mu}}_{c,i,j}{\hat{b}}_{r,i}{\hat{b}}_{r,j}{\hat{s}}_{r,j-i},{\hat{\alpha}}_{c,i,j}\right)}{\prod \limits_{c\in C}\prod \limits_{r\in {R}_c}f\left({x}_{r,i,j};{\hat{\mu}}_{0,i,j}{\hat{b}}_{r,i}{\hat{b}}_{r,j}{\hat{s}}_{r,j-i},{\hat{\alpha}}_{c,i,j}\right)} $$

where *f*(*x*; *μ*, *α*) is the negative binomial probability mass function (Equation 39). Under the null hypothesis, the likelihood ratio test statistic should asymptotically follow a chi-square distribution (Equation 51):

51 $$ -2\log {\lambda}_{i,j}\sim {\chi}_{\mid C\mid -1}^2 $$

where the degrees of freedom |*C*| − 1 of the *χ*^2^ distribution matches the difference in degrees of freedom between the null and alternate models. We used this asymptotic distribution to call right-tail *p* values for each pixel (*i*, *j*) (Equation 52):

52 $$ {p}_{i,j}=P\left({\chi}_{\mid C\mid -1}^2\ge -2\log {\lambda}_{i,j}\right) $$

#### Per-condition loop calling

While our model is capable of calling *p* values for differential interaction strength across conditions at all pixels within our chosen interaction distance range (40 kb to 5 Mb) and with an average normalized count across replicates of the same condition ${\overline{z}}_{\mathrm{c},i,j}$ of at least 1, we were primarily interested in specifically identifying differential interactions occurring specifically at loops. Therefore, in addition to the raw contact matrices used for the statistical analysis described above, 3DeFDR-HiC also takes as input a list of loops identified in any of the individual conditions analyzed, called by any external loop calling algorithm. For our main analysis, we used a loop call set which we obtained from merged raw contact matrices from all four replicates of both the ES and NPC conditions in the Bonev et al. dataset, processing these two conditions independently. We identified loops using an approach detailed elsewhere [69] with only a few minor changes in parameters. To parameterize the size of the donut filters, we used *p* = 2 and *w* = 6. We skipped the lambda chunking step; instead, we used BH-FDR directly on the set of all *p* values called across all chromosomes to obtain *q* values. Finally, for thresholding significant loops within conditions, we used a *q* value threshold of 0.025, a minimum cluster size of 4, and we excluded loop clusters with interaction distances smaller than 40 kb.

#### False discovery rate control

To control the false discovery rate given the large number of pixels being tested for differential looping, we first discarded all pixels (*i*, *j*) which were not involved in loops present in any of the condition, as determined by the per-condition loop calls described above. We then applied Benjamini-Hochberg false discovery rate (BH-FDR) control to the *p* values *p*_*i*,*j*_ at all remaining pixels across all chromosomes to obtain corresponding *q* values *q*_*i*,*j*_ for each loop pixel.

#### Clustering significantly differential interactions

We have previously observed that true looping interactions typically involve multiple adjacent pixels in the contact matrix. The same appears to be true for differential looping interactions. In order to avoid false positives caused by single significant pixels that are not supported by other adjacent significant pixels, we clustered all significant pixels at the chosen FDR threshold into clusters of contiguous, directly adjacent pixels and discarded pixels lying in clusters smaller than a specific minimum size threshold. We repeated this process for the insignificant pixels. For the final calls presented in Fig. 6, we selected an FDR threshold of 1% and a minimum cluster size threshold of 3.

#### Classifying significantly differential interactions

For simplicity, we classified each differential pixel (*i*, *j*) as specific to whichever condition in which its bias- and size factor-normalized mean value ${\overline{z}}_{c,i,j}$ (as defined in Equation 43) is highest. This approach works well for our Hi-C example analysis, in which there are only two conditions. More complicated experimental designs may require more sophisticated labeling of the differential interactions.

#### Simulating raw contact matrices

To simulate raw contact matrices, we started from the mean of bias- and size factor-normalized data for each pixel across replicates within a chosen condition. We used the ES condition, so this corresponds to ${\overline{z}}_{\mathrm{ES},i,j}$ as defined in Equation 43 above. We used ${\overline{z}}_{\mathrm{ES},i,j}$ as a starting point from which we create similar matrices for two new artificial conditions *A* and *B*. We formed the condition-specific true interaction strength matrices for these new artificial conditions, *μ*_*A*,*i*,*j*_ and *μ*_*B*,*i*,*j*_, by starting from ${\overline{z}}_{\mathrm{ES},i,j}$ and perturbing some looping clusters present in the real ES dataset (as indicated by our externally obtained loop calls). To make our simulations, we chose an effect size *β* and a proportion of truly differential loops *p*_diff_. At random, we labeled each real ES loop as constitutive with probability 1 − *p*_diff_ (leaving it unchanged across the two artificial conditions) and truly differential with probability *p*_diff_. The truly differential loops were then assigned into four differential categories with equal probability ($\frac{p_{\mathrm{diff}}}{4}$ each): up in *A*, down in *A*, up in *B*, and down in *B*. If a loop was labeled as up in *A*, we perturbed the loop by increasing the values of *μ*_*A*,*i*,*j*_ under the loop by adding *β* × *μ*_*A*,*i*,*j*_, where *β* represents an effect size in terms of a percentage change from the original interaction strength. In order to preserve the smooth appearance of the contact heatmap, we also perturbed pixels adjacent to the loop by half of this effect size. If a loop was labeled as down in *A*, we instead subtracted *β* × *μ*_*A*,*i*,*j*_ from the values of *μ*_*A*,*i*,*j*_ under the loop. If each loop pixel (*i*, *j*) is labeled according to the differential or constitutive label of the loop it lies under, the construction of *μ*_*A*,*i*,*j*_ can be written as follows (Equation 53):

53 $$ {\mu}_{A,i,j}=\left\{\begin{array}{ccc}{\overline{z}}_{\mathrm{ES},i,j}&, & \left(i,j\right)\kern0.18em \mathrm{is}\ \mathrm{constitutive},\mathrm{down}\ \mathrm{in}\kern0.18em B,\mathrm{or}\ \mathrm{up}\ \mathrm{in}\kern0.18em B\left(1-\frac{p_{\mathrm{diff}}}{2}\right)\\ {}{\overline{z}}_{\mathrm{ES},i,j}+\beta \times {\overline{z}}_{\mathrm{ES},i,j}&, & \left(i,j\right)\kern0.18em \mathrm{is}\ \mathrm{up}\ \mathrm{in}\kern0.18em A\left(\frac{p_{\mathrm{diff}}}{4}\right)\\ {}{\overline{z}}_{\mathrm{ES},i,j}-\beta \times {\overline{z}}_{\mathrm{ES},i,j}&, & \left(i,j\right)\kern0.18em \mathrm{is}\ \mathrm{down}\ \mathrm{in}\kern0.18em A\left(\frac{p_{\mathrm{diff}}}{4}\right)\\ {}{\overline{z}}_{\mathrm{ES},i,j}+\frac{\beta }{2}\times {\overline{z}}_{\mathrm{ES},i,j}&, & \left(i,j\right)\kern0.18em \mathrm{is}\ \mathrm{not}\ \mathrm{a}\ \mathrm{loop}\ \mathrm{but}\ \mathrm{a}\mathrm{ny}\ \mathrm{a}\mathrm{djacent}\ \mathrm{pixel}\ \mathrm{is}\ \mathrm{up}\ \mathrm{in}\kern0.18em A\\ {}{\overline{z}}_{\mathrm{ES},i,j}-\frac{\beta }{2}\times {\overline{z}}_{\mathrm{ES},i,j}&, & \left(i,j\right)\kern0.18em \mathrm{is}\ \mathrm{not}\ \mathrm{a}\ \mathrm{loop}\ \mathrm{but}\ \mathrm{a}\mathrm{ny}\ \mathrm{a}\mathrm{djacent}\ \mathrm{pixel}\ \mathrm{is}\ \mathrm{down}\ \mathrm{in}\kern0.18em A\\ {}{\overline{z}}_{\mathrm{ES},i,j}&, & \left(i,j\right)\kern0.18em \mathrm{is}\ \mathrm{not}\ \mathrm{a}\ \mathrm{loop}\ \mathrm{and}\ \mathrm{no}\ \mathrm{a}\mathrm{djacent}\ \mathrm{pixel}\ \mathrm{is}\ \mathrm{differential}\ \mathrm{in}\kern0.18em A\end{array}\right) $$

where the values in parentheses represent the probability that a loop will receive the indicated label. We perturbed the loops labeled as up in *B* and down in *B* in an analogous manner (Equation 54):

54 $$ {\mu}_{B,i,j}=\left\{\begin{array}{ccc}{\overline{z}}_{\mathrm{ES},i,j}&, & \left(i,j\right)\;\mathrm{is}\ \mathrm{constitutive},\mathrm{down}\ \mathrm{in}\kern0.18em A,\mathrm{or}\ \mathrm{up}\ \mathrm{in}\kern0.18em A\left(1-\frac{p_{\mathrm{diff}}}{2}\right)\\ {}{\overline{z}}_{\mathrm{ES},i,j}+\beta \times {\overline{z}}_{\mathrm{ES},i,j}&, & \left(i,j\right)\kern0.18em \mathrm{is}\ \mathrm{up}\ \mathrm{in}\kern0.36em B\left(\frac{p_{\mathrm{diff}}}{4}\right)\\ {}{\overline{z}}_{\mathrm{ES},i,j}-\beta \times {\overline{z}}_{\mathrm{ES},i,j}&, & \left(i,j\right)\kern0.18em \mathrm{is}\ \mathrm{down}\ \mathrm{in}\kern0.18em B\left(\frac{p_{\mathrm{diff}}}{4}\right)\\ {}{\overline{z}}_{\mathrm{ES},i,j}+\frac{\beta }{2}\times {\overline{z}}_{\mathrm{ES},i,j}&, & \left(i,j\right)\kern0.18em \mathrm{is}\ \mathrm{not}\ \mathrm{a}\ \mathrm{loop}\ \mathrm{but}\ \mathrm{a}\mathrm{ny}\ \mathrm{a}\mathrm{djacent}\ \mathrm{pixel}\ \mathrm{is}\ \mathrm{up}\ \mathrm{in}\kern0.18em B\\ {}{\overline{z}}_{\mathrm{ES},i,j}-\frac{\beta }{2}\times {\overline{z}}_{\mathrm{ES},i,j}&, & \left(i,j\right)\kern0.18em \mathrm{is}\ \mathrm{not}\ \mathrm{a}\ \mathrm{loop}\ \mathrm{but}\ \mathrm{a}\mathrm{ny}\ \mathrm{a}\mathrm{djacent}\ \mathrm{pixel}\ \mathrm{is}\ \mathrm{down}\ \mathrm{in}\kern0.18em B\\ {}{\overline{z}}_{\mathrm{ES},i,j}&, & \left(i,j\right)\kern0.18em \mathrm{is}\ \mathrm{not}\ \mathrm{a}\ \mathrm{loop}\ \mathrm{and}\ \mathrm{no}\ \mathrm{a}\mathrm{djacent}\ \mathrm{pixel}\ \mathrm{is}\ \mathrm{differential}\ \mathrm{in}\kern0.18em B\end{array}\right) $$

Having obtained perturbed artificial condition-specific true interaction strength matrices *μ*_*A*,*i*,*j*_ and *μ*_*B*,*i*,*j*_, we next biased them using bias vectors $\hat{b}$ and size factors $\hat{s}$ estimated from the real dataset’s ES replicates to obtain matrices of biased mean values *μ*_*p*,*i*,*j*_ for each simulated pseudoreplicate (Equation 55):

55 $$ {\mu}_{p,i,j}^{\mathrm{biased}}={\mu}_{c(p),i,j}{\hat{b}}_{r(p),i}{\hat{b}}_{r(p),j}{\hat{s}}_{r(p),j-i},\kern0.5em p\in \left\{A1,A2,B1,B2\right\} $$

where *p* ∈ {*A*1, *A*2, *B*1, *B*2} represents a simulated pseudoreplicate, *c*(*p*) ∈ {*A*, *B*} represents the artificial condition that simulated replicate *p* belongs to, and *r*(*p*) ∈ {ES _ 1, ES _ 3} represents a real ES replicate that has been matched to the simulated pseudoreplicate *p*. Finally, we simulated raw contact matrices *X*_*p*, *i*, *j*_ for each simulated pseudoreplicate *p* from the model (Equation 56):

56 $$ {X}_{p,i,j}\sim \mathrm{NB}\left({\mu}_{p,i,j}^{\mathrm{biased}},{\hat{\alpha}}_{\mathrm{ES},j-i}\right) $$

where ${\mu}_{p,i,j}^{\mathrm{biased}}$ is the pseudoreplicate-specific biased mean matrix for a simulated pseudoreplicate replicate *p* in artificial condition *c*(*r*) ∈ {*A*, *B*} and ${\hat{\alpha}}_{\mathrm{ES},j-i}$ is the final ES condition-specific distance-dependent dispersion value estimated from the real ES data (as defined in Equation 48). Examples of a simulated loop at the range of effect sizes are shown in Fig. 7a.

#### Evaluating model performance on simulated Hi-C data

Having simulated raw contact matrices with known true differential loops, we could repeat our differential looping analysis on these simulated matrices and compare the results to the locations of the truly differential loops. In order to perform the comparison, we first constructed a Boolean variable *t*_*i*,*j*_ which is one whenever the pixel at (*i*, *j*) is part of a truly differential loop and is zero otherwise. We then used a receiver operating characteristic (ROC) curve to assess how well the score 1 − *q*_*i*,*j*_ (where *q*_*i*,*j*_ is the *q* value against the null hypothesis that the pixel is a differential interaction) predicted the true differential status *t*_*i*,*j*_ of each pixel (*i*, *j*) which is part of a loop present in the real condition used to generate the simulations (in this case, ES) as determined by the external loop call set. We used this approach to plot the ROC curves shown in Fig. 7c, Additional file 2: Fig. S12D, and Additional file 2: Fig. S13D. We also computed the false discovery rate (FDR) at each FDR threshold along the ROC curve (by binarizing the score 1 − *q*_*i*,*j*_ using each FDR threshold and using the confusion matrix to directly determine the FDR) to draw the FDR control curves shown in Fig. 7d and Additional file 2: Fig. S13C. Finally, we used this same approach to plot power (the true positive rate, or one minus the type II error rate) as a function of FDR threshold as shown in Fig. 7e.

To assess the performance of our models at different distances scales, we stratified all loops in our real ES call set into short (< 400 kb), medium (400–800 kb), or long (> 800 kb) subsets and assessed the performance of our models at each distance subset using ROC curves (Additional file 2: Fig. S13D) and FDR control curves (Additional file 2: Fig. S13C). When drawing the FDR control curves for each distance subset, we re-ran the BH-FDR control procedure on the subset of *p* values falling in that distance subset to avoid the possibility that a true positive rate (TPR) at short distances might artificially inflate the FDR at long distances. Since our power (or equivalently, TPR) is lower at long distances where there are fewer reads (Additional file 2: Fig. S13D), it is expected that the proportion of false discoveries will be higher at longer distances. Because they are generated by re-running the BH-FDR step on every distance subset, our distance subset FDR control curves are adjusted for this effect.

To directly measure the extent to which our models showed a bias for calling differential loops at different distance scales, we quantified the proportion of loop pixels in each of the distance subsets described above with a *p* value below the 5th percentile of all *p* values in all loop pixels (irrespective of interaction distance). To rule out the possibility that low *p* values are enriched in regions where loops are more often truly differential, we performed this quantification using simulated data containing no truly differential loops. We used this approach to generate the bar plots in Additional file 2: Fig. S13E.

#### Comparison to simplified alternative models

To justify certain choices made in the design of our model, we compared its performance to that of simplified alternative models. These comparisons are visualized in Additional file 2: Fig. S13.

In our Poisson model, we replaced the negative binomial distribution with the simpler Poisson distribution, which does not account for any possible overdispersion in the data. Under this model, dispersion was not estimated or used directly, but was effectively assumed to be equal to zero for all pixels (Additional file 2: Fig. S13A, Poisson model). Under this model, the likelihood ratio (corresponding to Equation 50 above) becomes (Equation 57):

57 $$ {\lambda}_{i,j}^{\mathrm{Pois}}=\frac{\prod \limits_{c\in C}\prod \limits_{r\in {R}_c}{f}^{\mathrm{Pois}}\left({x}_{r,i,j};{\hat{\mu}}_{c,i,j}^{\mathrm{Pois}}{\hat{b}}_{r,i}{\hat{b}}_{r,j}{\hat{s}}_{r,j-i}\right)}{\prod \limits_{c\in C}\prod \limits_{r\in {R}_c}{f}^{\mathrm{Pois}}\left({x}_{r,i,j};{\hat{\mu}}_{0,i,j}^{\mathrm{Pois}}{\hat{b}}_{r,i}{\hat{b}}_{r,j}{\hat{s}}_{r,j-i}\right)} $$

where *f*^Pois^(*x*; *μ*) is the Poisson probability mass function (Equation 58):

58 $$ {f}^{\mathrm{Pois}}\left(x;\mu \right)=\frac{\mu^x{e}^{-\mu }}{x!} $$

and ${\hat{\mu}}_{c,i,j}^{\mathrm{Pois}}$ and ${\hat{\mu}}_{0,i,j}^{\mathrm{Pois}}$ are the mean parameters of this Poisson model, estimated via maximum likelihood while holding all other model parameters fixed.

This model performed reasonably well on true positive simulations in terms of sensitivity and specificity (Additional file 2: Fig. S13D) but failed to control type I error in simulations containing only constitutive loops (Additional file 2: Fig. S13B, Poisson model) and failed to control FDR in simulations containing some truly differential loops (Additional file 2: Fig. S13C, orange curve).

In our unsmoothed dispersion model, we used pixel-wise sample variance estimates to parameterize our negative binomial (NB) distributions, entirely forgoing pooling of pixels during variance estimation (Additional file 2: Fig. S13A, Sample Variance). We first computed pixel-wise sample variance estimates ${\hat{\sigma}}_{c,i,j}^2$ within each condition *c* (Equation 59):

59 $$ {\hat{\sigma}}_{c,i,j}^2=\frac{1}{\left|{R}_c\right|-1}\sum \limits_{r\in {R}_c}{\left({z}_{r,i,j}-{\overline{z}}_{c,i,j}\right)}^2 $$

where *R*_*c*_ = {*r* ∈ *R* ∣ *c*(*r*) = *c*} represents the set of replicates in condition *c*; ${z}_{r,i,j}=\frac{x_{r,i,j}}{{\hat{b}}_{r,i}{\hat{b}}_{r,j}{\hat{s}}_{r,j-i}}$ represents the interaction count for the interaction between the *i*th and *j*th bin in replicate *r*, normalized for locus-specific bias and library size; and ${\overline{z}}_{c,i,j}$ is the pixel-wise mean of the *z*_*r*, *i*, *j*_ for all *r* ∈ *R*_*c*_ as defined in Equation 43. To parameterize the negative binomial distributions with variance equal to ${\hat{\sigma}}_{c,i,j}^2$ in a manner consistent with our existing framework, we used the properties of the NB distribution to find the dispersion ${\hat{\alpha}}_{c,i,j}^{\mathrm{SV}}$ such that $\mathrm{Var}\left[\mathrm{NB}\left({\overline{z}}_{c,i,j},{\hat{\alpha}}_{c,i,j}^{\mathrm{SV}}\right)\right]={\hat{\sigma}}_{c,i,j}^2$. Because the negative binomial distribution is undefined when dispersion is less than or equal to zero, we enforced a minimum value for ${\hat{\alpha}}_{c,i,j}^{\mathrm{SV}}$ of 1e−7 to avoid numerical instability (Equation 60):

60 $$ {\hat{\alpha}}_{c,i,j}^{\mathrm{SV}}=\max \left(1\times {10}^{-7},\frac{{\hat{\sigma}}_{c,i,j}^2-{\overline{z}}_{c,i,j}}{{\overline{z}}_{c,i,j}^2}\right) $$

This model suffered from reduced specificity and sensitivity compared to our pooled dispersion model (Additional file 2: Fig. S13D, red curve).

In our global dispersion model, we used qCML to fit a single dispersion value for each condition, forgoing the fitting of a trend between dispersion and mean (Additional file 2: Figure S13A, Global NB) to obtain a single dispersion estimate for each condition *c*, which we denote ${\hat{\alpha}}_c^{\mathrm{global}}$. In this fitting, we included all pixels which are part of looping interactions, independent of their interaction distance. This approach ignores a clear trend between dispersion and distance visible in the real data (Fig. 6). This model performed reasonably well on true positive simulations in terms of sensitivity and specificity (Additional file 2: Figure S13D) but showed a strong bias for calling more differential interactions at longer distances and fewer differential interactions at shorter distances (Additional file 2: Figure S13D, green curve and Additional file 2: Figure S13E, green bars).

#### Comparison to diffHic

In addition to comparing against our own simplified models, we also compared he performance of 3DeFDR-HiC to diffHiC [45] genome-wide on a simulated dataset based on the ES condition of the real Bonev dataset, setting 40% of ES loops to be differential with an effect size of ± 30% (Additional file 6: Table S5). We applied diffHiC following the package’s provided usage guidelines for Hi-C data. We inputted our Hi-C dataset as raw, binned (10 kb wide bins) counts. Library sizes were inputted as the total number of counts in each replicate. Following the diffHic user guide, we first filtered rows with any NaN counts. Then we filtered by average abundance (diffHic user guide section 4.1), discarding any bin-bin pair with an average abundance across all sample replicates of less than 5. We then used the direct filter (diffHic user guide section 4.2) to directly remove low-abundance bin-bin pairs, discarding any with abundances less than 2-fold higher than the estimated non-specific ligation rate (estimated as the median abundance across bin-bin pairs). We chose 2-fold above this estimate as our threshold instead of 5-fold, as presented in the usage instructions’ example, because we found this threshold discarded too many points for us to perform later steps in the diffHiC pipeline. Next as recommended (diffHic user guide section 4.3), we filtered bin-bin pairs as a function of interaction distance, discarding pairs with abundances less than the value expected from compaction (generated using the filterTrended method, no parameters to set) choosing not to increase this threshold by an additional fold change value. Finally, we filtered bin-bin pairs via the peak calling approach (diffHic user guide section 4.4) using the guide recommended values for the filter’s parameters (flank.width = 5, the minimum threshold for peak enrichment min.enrich at 0.17, the minimum threshold for peak counts min.count at 3, and the near diagonal cut-off min.diag of 2L). Next, we applied non-linear normalization (diffHic user guide section 5.2) to remove trended biases between libraries, specifically using LOESS normalization, using type=“loess” for the normOffsets method. We also separately filtered near diagonal points, using by.dist=1.5e6 with method filterDiag and again LOESS normalization using type=“loess” with method normOffSets as presented in the user guide (second half of diffHic section 5.2.1).

We then modeled biological variability between replicates. First, we estimated negative binomial dispersion for each bin-bin pair (diffHic user guide section 6.1–6.2) using a single-factor design matrix design = model.matrix (~conditions) where conditions is the corresponding list of the biological condition label for the input sample set. We performed estimation of negative binomial dispersion using this design. We then estimated QL dispersion using this design as recommended in the user guide, setting robust = TRUE for method glmQLFit (diffHic user guide section 6.3).

Finally, we tested for significantly differential interactions using the quasi-likelihood *F*-test via method glmQLFTest (diffHic user guide section 7.1; no parameters to set) and obtained *p* values adjusted to correct for multiple testing using the Benjamini-Hochberg (BH) method (diffHic user guide section 7.2). We then saved output log fold change and adjusted *p* values for each bin-bin pair to file and then visualized these results using our own tools. We thresholded bin-bin pairs to an FDR threshold, assigned loop classifications according to the sign of the log fold change value, and finally plotted these classifications spatially. Specifically, in Additional file 2: Figure S12, we ran applied this scheme with FDR thresholds of 0.3 (or 30%), 0.25, 0.2, 0.15, 0.1, 0.05, 0.01, and 0.001. We used the true labels from our simulations, the adjusted *p* values from diffHic, and the *q* values called by 3DeFDR-HiC to draw ROC curves comparing the performance of the two methods (Additional file 2: Figure S12D).

## Supplementary information

**Additional file 1: Table S1.** 5C datasets analyzed in this study.

**Additional file 2: Figures S1-S14.** This file contains all supplementary figures.

**Additional file 3: Table S2.** 3DeFDR criteria for differential loop classification.

**Additional file 4: Table S3.** Correlations between real and simulated 5C replicates.

**Additional file 5: Table S4.** ChIP-seq datasets analyzed in this study.

**Additional file 6: Table S5.** Hi-C datasets analyzed in this study.

**Additional file 7.** Review history.

## Data Availability

Code, sample input data, and usage instructions are available on Bitbucket at the following URLs: https://bitbucket.org/creminslab/5c3defdr [70] (5C analysis package, available on zenodo at https://zenodo.org/record/3783843 with DOI 10.5281/zenodo.3783843 [71]) and https://bitbucket.org/creminslab/hic3defdr [72] (Hi-C analysis package, available on zenodo at https://zenodo.org/record/3783855 with DOI 10.5281/zenodo.3783855 [73]). Source code for both packages is provided under the MIT license. The data analyzed in this study are available on GEO with the following accession numbers: GSM2259911, GSM2259912, GSM2259913, GSM2259914, GSM2259915, GSM2259916, GSM2259905, GSM2259907, GSM2259909, GSM594579, GSM594585, GSM2259906, GSM2259908, GSM2259910, GSM594599, GSM883648, GSM2533818, GSM2533819, GSM2533820, GSM2533821, GSM2533822, GSM2533823, GSM2533824, and GSM2533825. The different data sources used are summarized in Additional file 1: Table S1, Additional file 5: Table S4, and Additional file 6: Table S5.
