# Supplementary material for: 3DeFDR: statistical methods for identifying cell type-specific looping interactions in 5C and Hi-C data
Source: Genome Biol. 2020 Aug 28;21:219. doi: 10.1186/s13059-020-02061-9 (PMC7496221; doi:10.1186/s13059-020-02061-9)
Supplement: Supplementary file 2 — Additional file 2: Figures S1-S14. This file contains all supplementary figures. [file 13059_2020_2061_MOESM2_ESM.pdf]

Supplementary Figures

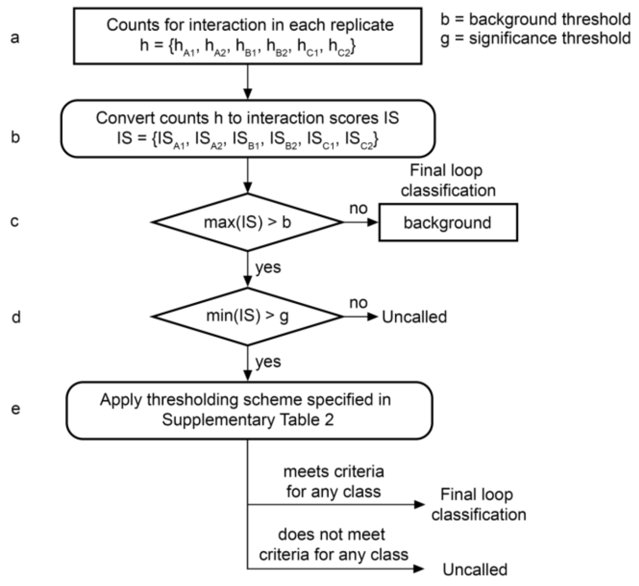

Figure S1: Overview schematic of 3DeFDR differential looping interaction classification procedure. (a) For each conditionally quantile normalized fragment-fragment interaction, we have a set h of six sample counts values with two samples collected for each of three cellular conditions A, B, and C. **(b)** 5C counts are matrix balanced, binned, modeled, and converted to interaction scores (IS) (detailed in Supplementary Methods) and then subjected to a thresholding scheme. **(c)** If all sample IS values are lower than a certain background threshold value b, the bin-bin pair is labeled as a background loop. **(d)** To be tested for differential looping, a bin-bin pair must have at least one sample interaction score in set IS that is greater than a given significance threshold g. (A bin-bin pair that passes the background threshold control point but is not above the significance threshold in any condition will not be subject to further analysis and not assigned any label.) **(e)** We determine the final differential loop classification by thresholding differences in IS across conditions against the difference threshold and IS in each condition against the significance threshold, as specified in **Additional file 3: Table S2**. Final loop classifications are assigned based on the criteria listed in **Additional file 3: Table S2** with a loop being characteristic of a single condition (e.g. A only, B only, or C only) if it is significant in that condition and differential when compared to remaining conditions with respect to IS. Similarly, a loop is characteristic of two conditions (e.g. A & B, B & C, or A & C) if is significant in those conditions and differential when compared to the single remaining condition.

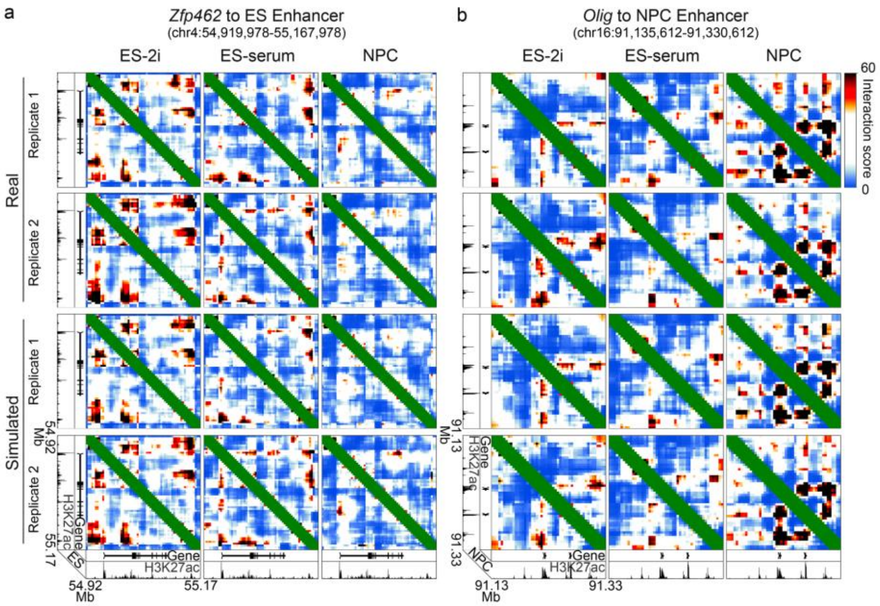

Figure S2: Real and simulated interaction score heatmaps for multiple replicates of each cell type. (a-b) Interaction score heatmaps for real and simulated ES-2i, ES-serum, and NPC 5C replicates. (**a**) Zoomed-in heatmap encompassing *Zfp462* to ES-specific enhancer interaction. Putative enhancer demarcated by enriched ES-serum H3K27ac signal underneath the looping anchors. (**b**) Zoomed-in heatmap encompassing *Olig1/2* to NPC-specific enhancer interaction. Putative enhancer demarcated by enriched NPC H3K27ac signal underneath the looping anchors.

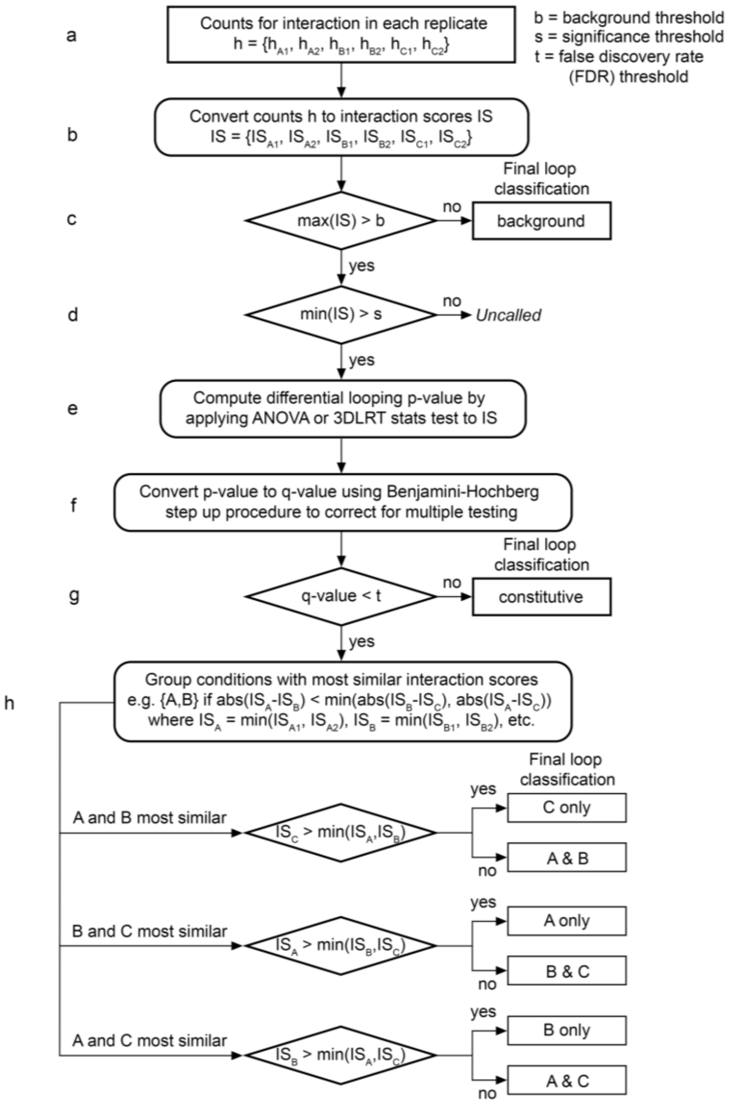

Figure S3: Overview schematic of ANOVA and 3DLRT differential looping interaction classification procedure. (a) For each conditionally quantile normalized fragment-fragment interaction, we have a set h of six sample counts values with two samples collected for each of three cellular conditions A, B, and C. **(b)** 5C counts are matrix balanced, binned, modeled, and converted to interaction scores (IS), or alternatively z-scores, (detailed in Supplementary Methods) and then subjected to a thresholding scheme. **(c-d)** To be tested for differential looping, a bin-bin pair must have at least one sample interaction score in set IS that is greater than a given significance threshold. Additionally, if all sample interaction scores in IS are lower than a specific background threshold value, the bin-bin pair is labeled as a background loop. A bin-bin pair that passes the background threshold control point but is not above the significance threshold in any condition will not be subject to further analysis and not assigned any label. **(e-f)** To identify a bin-bin pair as significantly differential across cellular conditions, we first compute its differential

looping p-value using either the ANOVA or 3DLRT statistical test. To account for multiple testing, p-values are adjusted to q-values with Benjamini-Hochberg. **(g)** If the resulting differential looping q-value is lower than a user-defined false discovery rate (FDR) threshold t, the interaction is classified as differential and otherwise is classified as non-differential or constitutive across conditions. **(h)** Differential interactions are further categorized according to the direction and fold change of their differential looping signal to obtain a final looping classification.

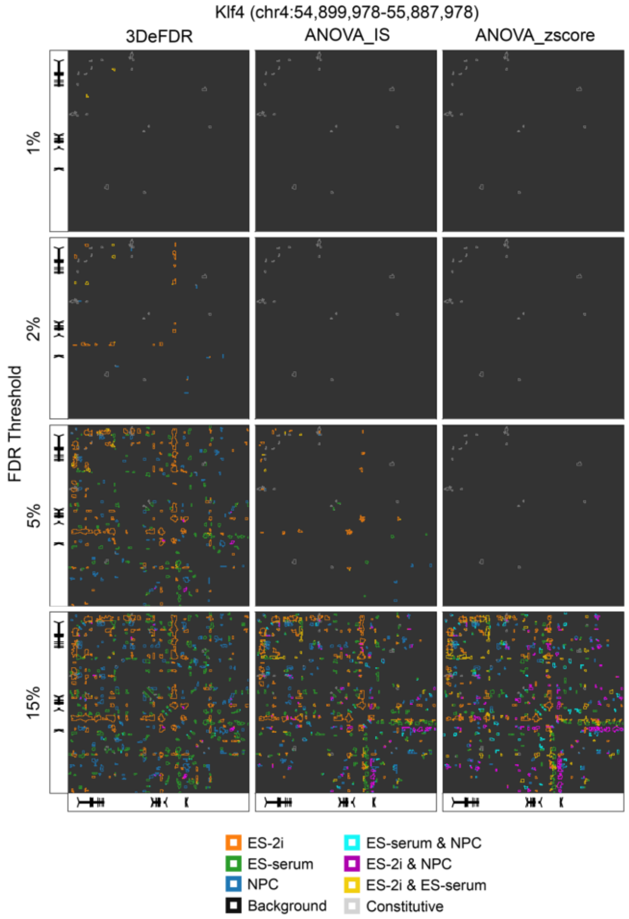

Figure S4: Benchmarking of 3DeFDR at the *Klf4* locus against ANOVA tests performed on interaction scores and z-scores. Looping interaction classes identified in the genomic region surrounding *Klf4* via 3DeFDR, ANOVA on interaction scores, and ANOVA on z-scores across a sweep of false discovery rate (FDR) thresholds. All three approaches used the interaction score as the random variable (see **Methods**).

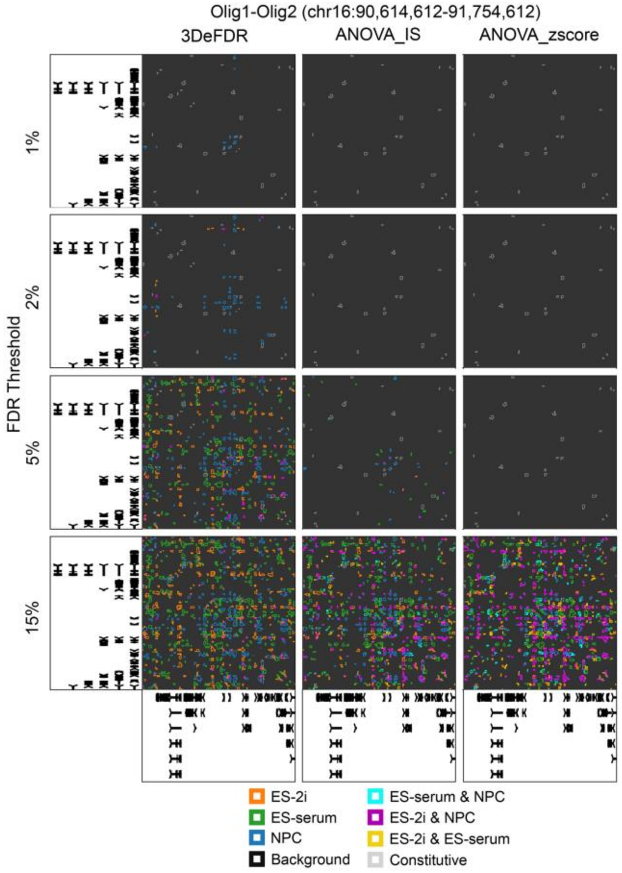

Figure S5: Benchmarking of 3DeFDR at the *Olig1-Olig2* locus against ANOVA tests performed on interaction scores and z-scores. Looping interaction classes identified in the genomic region surrounding *Olig1/2* via 3DeFDR, ANOVA on interaction scores, and ANOVA on z-scores across a sweep of false discovery rate (FDR) thresholds. All three approaches used the interaction score as the random variable (see **Methods**).

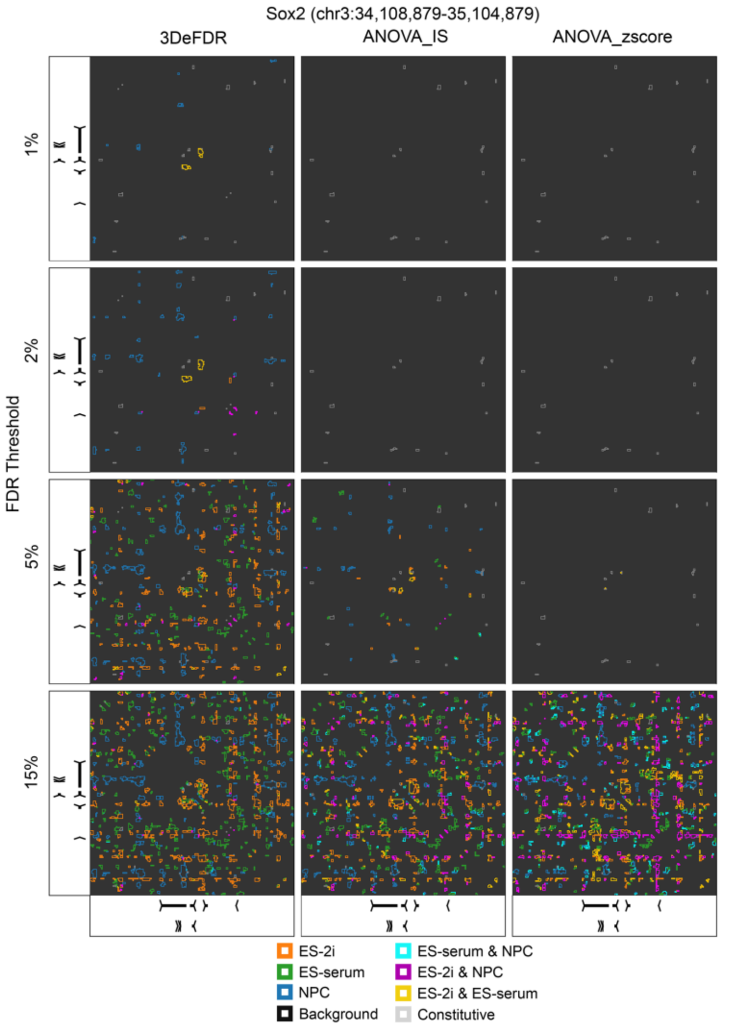

Figure S6: Benchmarking of 3DeFDR at the *Sox2* locus against ANOVA tests performed on interaction scores and z-scores. Looping interaction classes identified in the genomic region surrounding *Sox2* via 3DeFDR, ANOVA on interaction scores, and ANOVA on z-scores across a sweep of false discovery rate (FDR) thresholds. All three approaches used the interaction score as the random variable (see **Methods**).

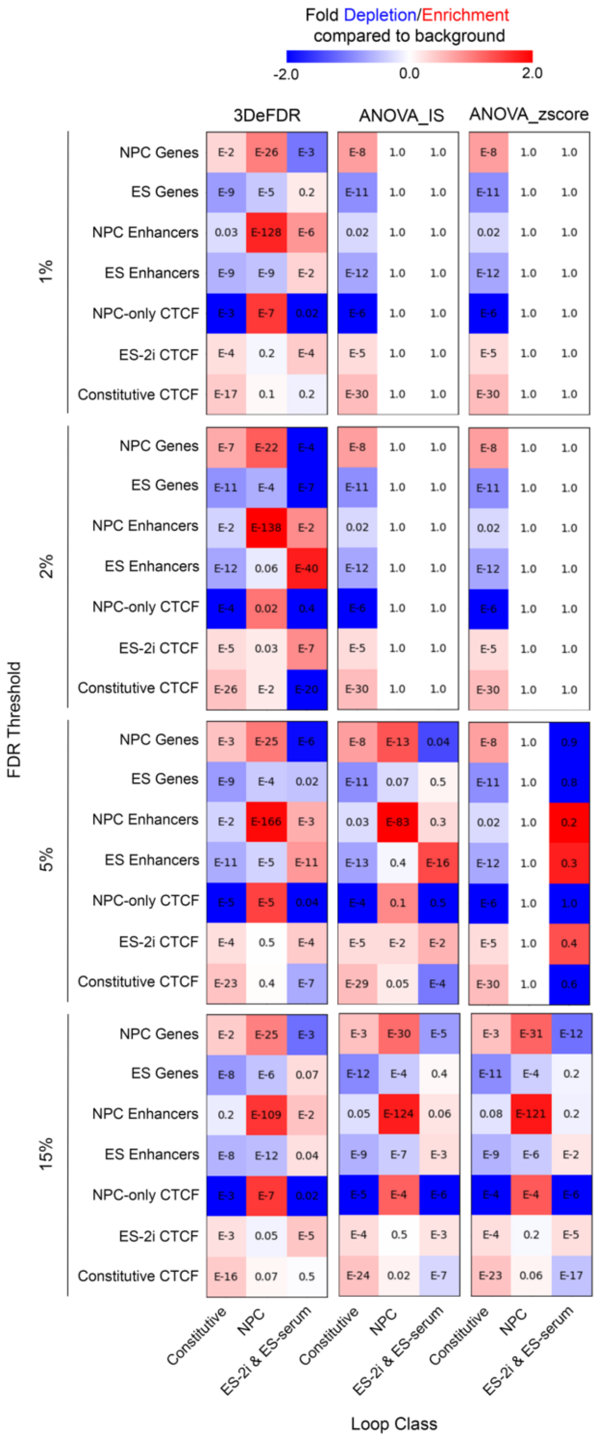

Figure S7: Fold change depletion/enrichment in cell-type specific chromatin features for differential loops classified via 3DeFDR, ANOVA performed on interaction scores, and ANOVA performed on z-scores. Fold change depletion/enrichment is computed as the proportion of binned interactions assigned to a given looping class (columns) that have at least one anchor occupied by a given

chromatin feature (rows) over the proportion of binned interactions labeled background which are likewise positive for that feature. P-values included within each entry are computed using Fischer's exact test.

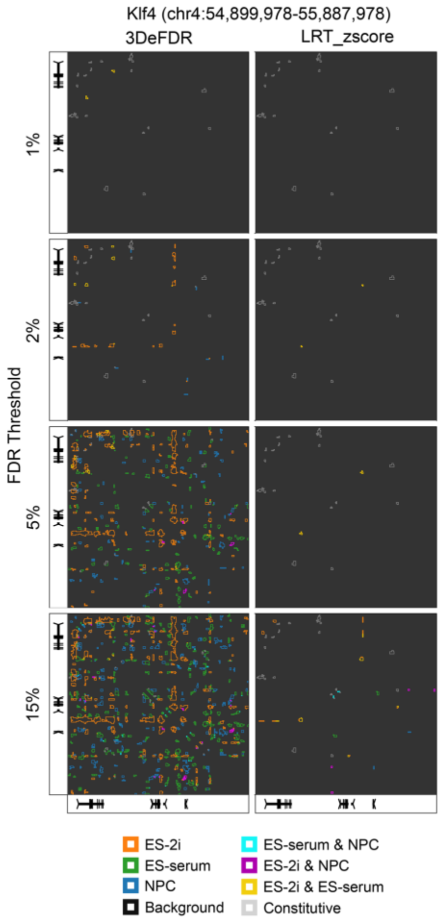

Figure S8: Benchmarking of 3DeFDR at the *Klf4* locus against 3DLRT performed on z-scores. Looping interaction classes identified in the genomic region surrounding *Klf4* via 3DeFDR and 3DLRT on z-scores across a sweep of false discovery rate (FDR) thresholds. All three approaches used the interaction score as the random variable (see **Methods**).

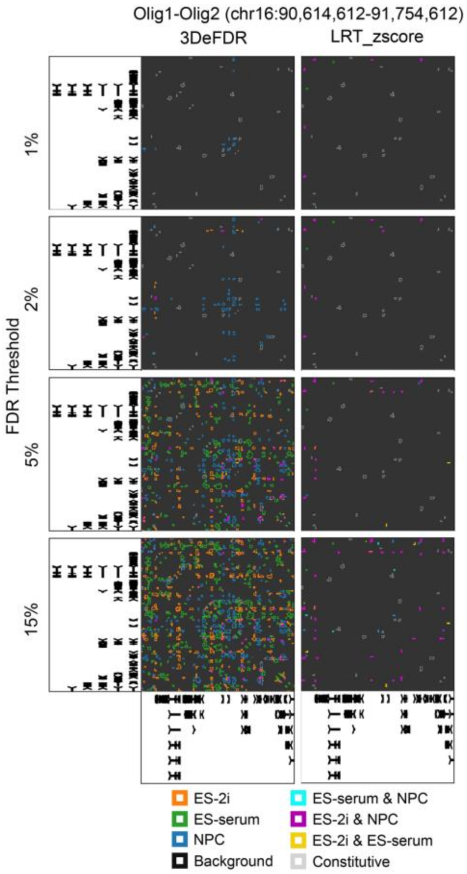

Figure S9: Benchmarking of 3DeFDR at the *Olig1-Olig2* locus against 3DLRT performed on z-scores. Looping interaction classes identified in the genomic region surrounding *Olig1/2* via 3DeFDR and 3DLRT on z-scores across a sweep of false discovery rate (FDR) thresholds. All three approaches used the interaction score as the random variable (see **Methods**).

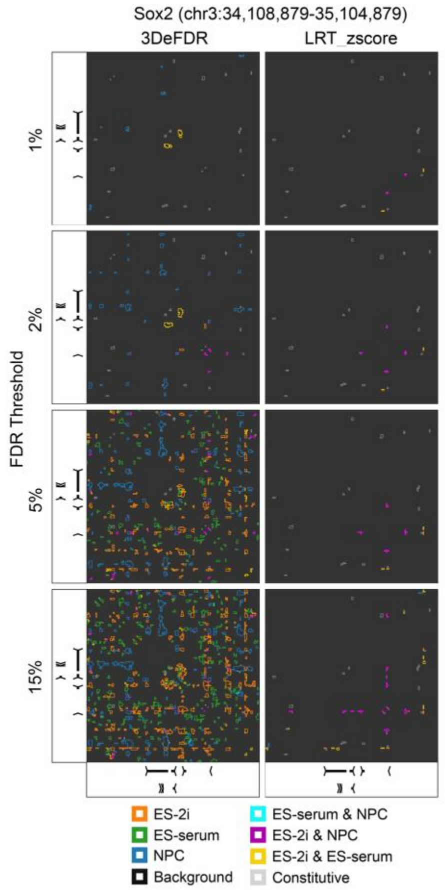

Figure S10: Benchmarking of 3DeFDR at the *Sox2* locus against 3DLRT performed on and z-scores. Looping interaction classes identified in the genomic region surrounding *Sox2* via 3DeFDR and 3DLRT on z-scores across a sweep of false discovery rate (FDR) thresholds. All three approaches used the interaction score as the random variable (see **Methods**).

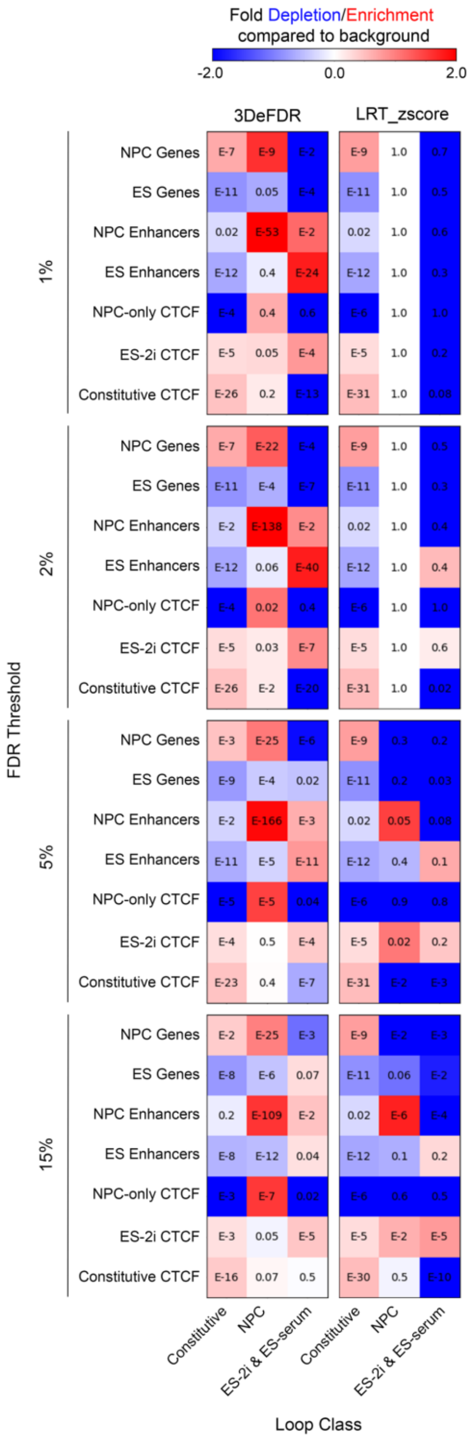

Figure S11: Fold change depletion/enrichment in cell-type specific chromatin features for differential loops classified via 3DeFDR and 3DLRT performed on z-scores. Fold change depletion/enrichment is computed as the proportion of binned interactions assigned to a given looping class (columns) that have at least one anchor occupied by a given chromatin feature (rows) over the proportion of binned

interactions labeled background which are likewise positive for that feature. P-values included within each entry are computed using Fischer's exact test.

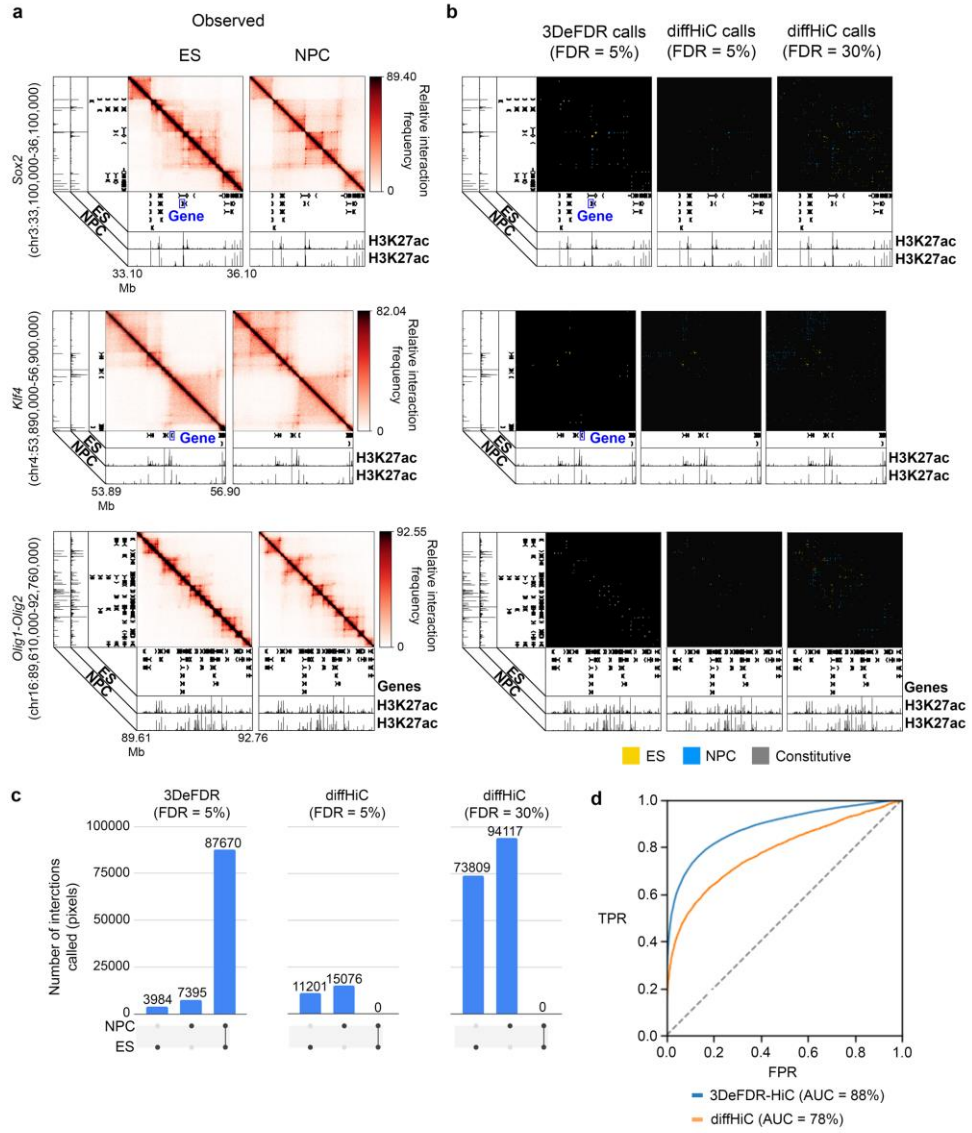

Figure S12: Differential loops classified from Hi-C data via 3DeFDR-HiC and diffHiC. (a) Reference heatmaps representing binned, quantile normalized, and joint express normalized Hi-C counts (Observed) around the *Sox2* (chr3:33,100,000-36,100,000), *Klf4* (chr4:53,890,000-56,900,000), *Olig1-Olig2* (chr16:89,610,000-92,760,000) genes. **(b)** Looping interaction classes identified across these regions in the Bonev Hi-C dataset. Loops are called using 3DeFDR-HiC with an FDR threshold of 5%, diffHiC at a matched FDR threshold of 5%, and diffHiC at an FDR of 30%. **(c)** UpSetR scalable Venn diagrams of the number of pixels called genome-wide as ES only, NPC only, or constitutive. **(d)** Receiver operating characteristic (ROC) curves showing performance of 3DeFDR-HiC and diffHiC on simulated Hi-C data. The x-axis shows the false positive rate (FPR), or one minus the specificity. The y-axis shows the true positive rate (TPR), or sensitivity. The area under the receiver operating characteristic curve (AUROC) for each curve is shown in parentheses in the legend.

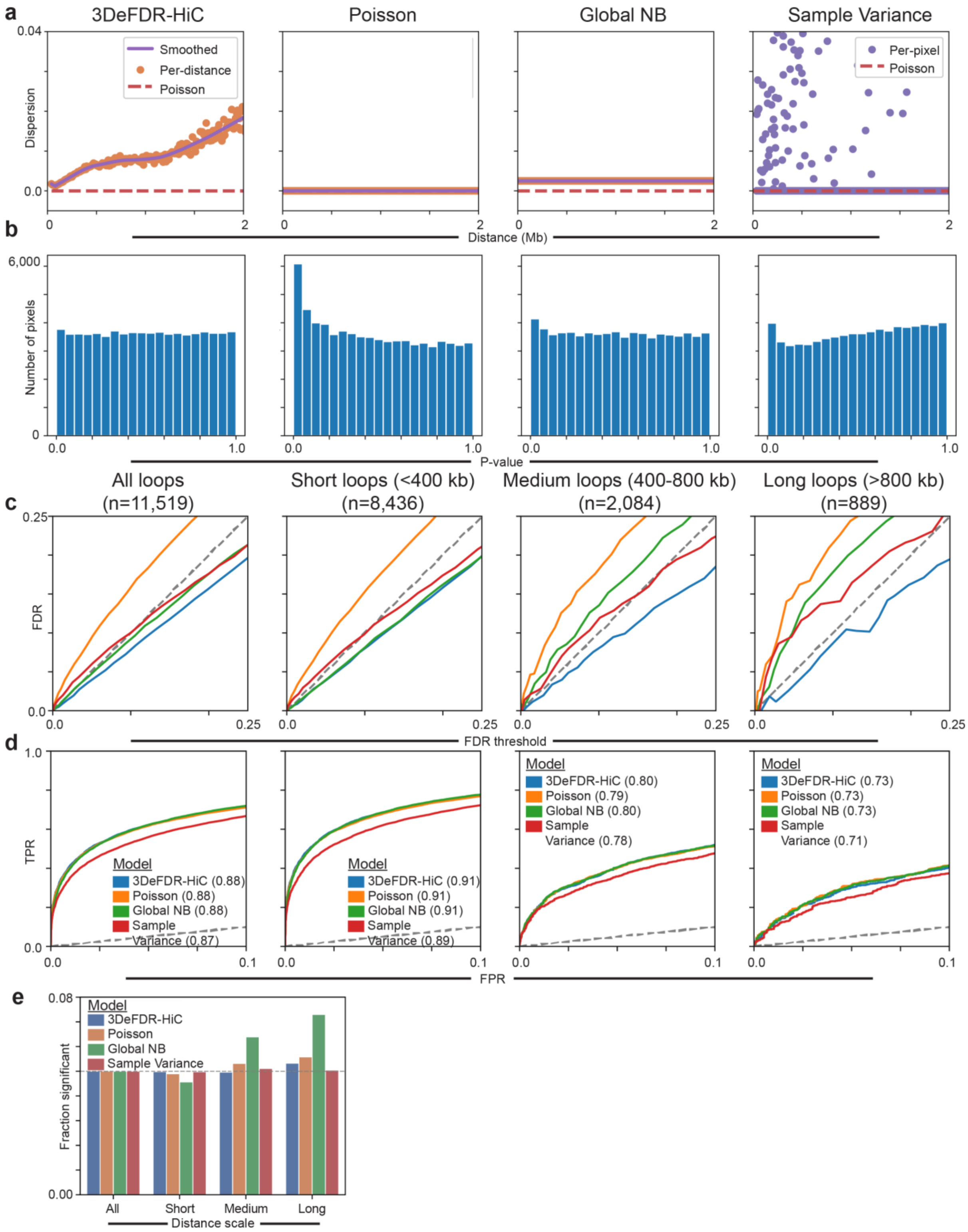

Figure S13: Additional comparison of performance of 3DeFDR-HiC method compared to alternative analysis strategies using simulated Hi-C data. (a) Dispersion-distance relationship of 3DeFDR-HiC compared to alternative analysis

strategies. The orange points represent per-distance dispersion estimates. The purple curves or points represent final dispersion values used at different distances. The 3DeFDR-HiC panel shows our method, which fits a trend through negative binomial (NB) dispersion parameters estimated separately at each distance. The Poisson panel shows the assumption implied by the use of the Poisson distribution, a limiting case of the NB distribution as dispersion approaches zero. The Global NB panel shows the results of fitting a single dispersion parameter to data from all distance scales. The Sample Variance panel shows the results of using the per-pixel sample variance across replicates directly without using any pooling. **(b)** Distributions of p-values called by each method on a simulated null dataset. Methods that successfully control type I error should show no enrichment for low p-values. The Poisson strategy fails to control type I error as evidenced by the enrichment for low p-values even though the dataset contains no truly differential interactions. **(c)** False discovery rate (FDR) control comparison of our method to alternative analysis strategies, colored as in (d), at a variety of distance scales. The x-axis shows a range of FDR thresholds, while the y-axis shows the actual FDR we observe in the differential calls made by each method at that FDR threshold. Methods that control FDR should stay below the dashed gray line. The FDR control curves are evaluated on simulations in which 20% of loops are truly differential with an effect size of +/-40%. The far-left panel shows the aggregate FDR control profile at all distance scales, while the other three panels show FDR control profiles specifically at short (< 400 kb interaction distance), medium (400 – 800 kb), and long (> 800 kb) distance scales. The Poisson strategy (shown in orange) fails to control FDR at all distance scales, especially at medium and long distance scales where dispersion is highest. The Global NB strategy (shown in green) is very conservative at short loops but too permissive at long loops, reflecting its failure to account for the distance-dispersion relationship. **(d)** Receiver operating characteristic (ROC) curves comparing performance of our method to alternative analysis strategies at the distance scales shown in (c). The x-axes show the false positive rate (FPR), or one minus the specificity. The y-axes show the true positive rate (TPR), or sensitivity. The ROC curves are evaluated on simulations in which 40% of loops are truly differential with an effect size of +/-30%. The far-left panel shows the aggregate ROC curves at all distance scales, while the other three panels show ROC curves specifically at short (< 400 kb interaction distance), medium (400 – 800 kb), and long (> 800 kb) distance scales. The area under the receiver operating characteristic curve (AUROC) for each curve is shown in parentheses in the legend. The Sample Variance strategy (shown in red) consistently underperforms the other options, possibly due to excessive variance in its variance estimates. Under our choice of simulation conditions, performance diminishes as distance increases, presumably due to the lower coverage at higher distances. **(e)** Bar plot showing the fraction of loop pixels whose p-values fall below the 5th percentile of all loop pixel p-values for the distance scale groups used in (c) and (d) in true null simulated data. The gray dashed line indicates the expected proportion of pixels that should fall below the 5th percentile if the method shows no bias with respect to distance scale. The Global NB method is too conservative at short distances and too permissive at long distances, reflecting its failure to account for the distance-dispersion relationship.

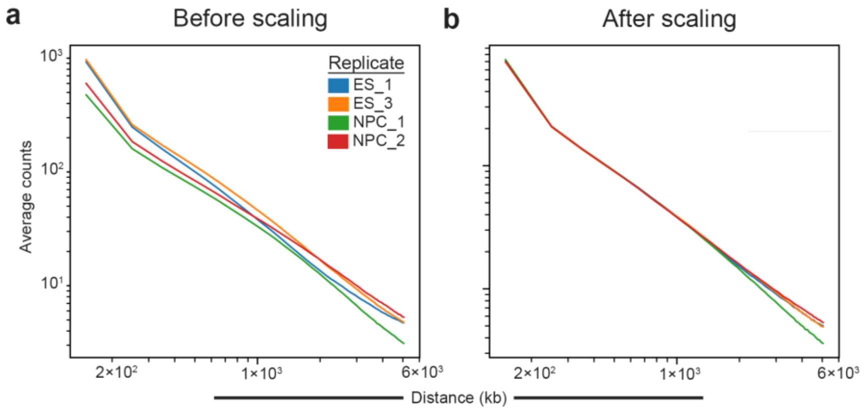

Figure S14: Characterization of distance dependent library size normalization. (a) Distance dependence curves of matrix balanced data on chromosome 1 before distance-dependent median of ratios library size normalization. The curves show the average read count at each distance scale for each replicate. **(b)** Same as (a), but after applying distance-dependent median of ratios library size normalization.
