# Supplementary material for: 3DeFDR: statistical methods for identifying cell type-specific looping interactions in 5C and Hi-C data
Source: Genome Biol. 2020 Aug 28;21:219. doi: 10.1186/s13059-020-02061-9 (PMC7496221; doi:10.1186/s13059-020-02061-9)
Supplement: Supplementary file 3 — Additional file 3: Table S2. 3DeFDR criteria for differential loop classification. [file 13059_2020_2061_MOESM3_ESM.docx]

**Table S2: 3DeFDR criteria for differential loop classification.**

| **Looping class** | **Significance criteria**  **(**$g$ significance threshold) | **Differential looping criteria**  **(**$d$ interaction score difference threshold) |
| --- | --- | --- |
| {A} | ${IS}_{A1}> g$  ${IS}_{A2}> g$ | ${IS}_{A1}- {IS}_{B1}>d$  ${IS}_{A1}- {IS}_{B2}>d$  ${IS}_{A2}- {IS}_{B1}>d$  ${IS}_{A2}- {IS}_{B2}>d$  ${IS}_{A1}- {IS}_{C2}>d$  ${IS}_{A2}- {IS}_{C1}>d$  ${IS}_{A2}- {IS}_{C2}>d$ |
| {B} | ${IS}_{B1}> g$  ${IS}_{B2}> g$ | ${IS}_{B1}- {IS}_{A1}>d$  ${IS}_{B1}- {IS}_{A2}>d$  ${IS}_{B2}- {IS}_{A1}>d$  ${IS}_{B2}- {IS}_{A2}>d$  ${IS}_{B1}- {IS}_{C1}>d$  ${IS}_{B1}- {IS}_{C2}>d$  ${IS}_{B2}- {IS}_{C1}>d$  ${IS}_{B2}- {IS}_{C2}>d$ |
| {C} | ${IS}_{C1}> g$  ${IS}_{C2}> g$ | ${IS}_{C1}- {IS}_{A1}>d$  ${IS}_{C1}- {IS}_{A2}>d$  ${IS}_{C2}- {IS}_{A1}>d$  ${IS}_{C2}- {IS}_{A2}>d$  ${IS}_{C1}- {IS}_{B1}>d$  ${IS}_{C1}- {IS}_{B2}>d$  ${IS}_{C2}- {IS}_{B1}>d$  ${IS}_{C2}- {IS}_{B2}>d$ |
| {A, B} | ${IS}_{A1}> g$  ${IS}_{A2}> g$  ${IS}_{B1}> g$  ${IS}_{B2}> g$ | ${IS}_{A1}- {IS}_{C1}>d$  ${IS}_{A1}- {IS}_{C2}>d$  ${IS}_{A2}- {IS}_{C1}>d$  ${IS}_{A2}- {IS}_{C2}>d$  ${IS}_{B1}- {IS}_{C1}>d$  ${IS}_{B1}- {IS}_{C2}>d$  ${IS}_{B2}- {IS}_{C1}>d$  ${IS}_{B2}- {IS}_{C2}>d$  $\left\vert{IS}_{A1}- {IS}_{B1} \right\vert\leq d$  $\left\vert{IS}_{A1}- {IS}_{B2} \right\vert\leq d$  $\left\vert{IS}_{A2}- {IS}_{B1} \right\vert\leq d$  $\left\vert{IS}_{A2}- {IS}_{B2} \right\vert\leq d$ |
| {A, C} | ${IS}_{A1}> g$  ${IS}_{A2}> g$  ${IS}_{C1}> g$  ${IS}_{C2}> g$ | ${IS}_{A1}- {IS}_{B1}>d$  ${IS}_{A1}- {IS}_{B2}>d$  ${IS}_{A2}- {IS}_{B1}>d$  ${IS}_{A2}- {IS}_{B2}>d$  ${IS}_{C1}- {IS}_{B1}>d$  ${IS}_{C1}- {IS}_{B2}>d$  ${IS}_{C2}- {IS}_{B1}>d$  ${IS}_{C2}- {IS}_{B2}>d$  $\left\vert{IS}_{A1}- {IS}_{C1} \right\vert\leq d$  $\left\vert{IS}_{A1}- {IS}_{C2} \right\vert\leq d$  $\left\vert{IS}_{A2}- {IS}_{C1} \right\vert\leq d$  $\left\vert{IS}_{A2}- {IS}_{C2} \right\vert\leq d$ |
| {B, C} | ${IS}_{B1}> g$  ${IS}_{B2}> g$  ${IS}_{C1}> g$  ${IS}_{C2}> g$ | ${IS}_{B1}- {IS}_{A1}>d$  ${IS}_{B1}- {IS}_{A2}>d$  ${IS}_{B2}- {IS}_{A1}>d$  ${IS}_{B2}- {IS}_{A2}>d$  ${IS}_{C1}- {IS}_{A1}>d$  ${IS}_{C1}- {IS}_{A2}>d$  ${IS}_{C2}- {IS}_{A1}>d$  ${IS}_{C2}- {IS}_{A2}>d$  $\left\vert{IS}_{B1}- {IS}_{C1} \right\vert\leq d$  $\left\vert{IS}_{B1}- {IS}_{C2} \right\vert\leq d$  $\left\vert{IS}_{B2}- {IS}_{C1} \right\vert\leq d$  $\left\vert{IS}_{B2}- {IS}_{C2} \right\vert\leq d$ |
| Constitutive | ${IS}_{A1}> g$  ${IS}_{A2}> g$  ${IS}_{B1}> g$  ${IS}_{B2}> g$  ${IS}_{C1}> g$  ${IS}_{C2}> g$ | $\left\vert{IS}_{A1}- {IS}_{B1} \right\vert\leq d$  $\left\vert{IS}_{A1}- {IS}_{B2} \right\vert\leq d$  $\left\vert{IS}_{A2}- {IS}_{B1} \right\vert\leq d$  $\left\vert{IS}_{A2}- {IS}_{B2} \right\vert\leq d$  $\left\vert{IS}_{A1}- {IS}_{C1} \right\vert\leq d$  $\left\vert{IS}_{A1}- {IS}_{C2} \right\vert\leq d$  $\left\vert{IS}_{A2}- {IS}_{C1} \right\vert\leq d$  $\left\vert{IS}_{A2}- {IS}_{C2} \right\vert\leq d$  $\left\vert{IS}_{B1}- {IS}_{C1} \right\vert\leq d$  $\left\vert{IS}_{B1}- {IS}_{C2} \right\vert\leq d$  $\left\vert{IS}_{B2}- {IS}_{C1} \right\vert\leq d$  $\left\vert{IS}_{B2}- {IS}_{C2} \right\vert\leq d$ |

***** We use the shorthand ${IS}_{A1}$ to refer to the sample interaction score of a single loop between bins *k* and *l* in region *r* for replicate 1 of condition A, a value denoted ${IS}_{A_{1}, r, k, l}$ in the Supplementary Methods.
