# Supplementary material for: 3DeFDR: statistical methods for identifying cell type-specific looping interactions in 5C and Hi-C data
Source: Genome Biol. 2020 Aug 28;21:219. doi: 10.1186/s13059-020-02061-9 (PMC7496221; doi:10.1186/s13059-020-02061-9)
Supplement: Supplementary file 7 — Additional file 7. Review history. [file 13059_2020_2061_MOESM7_ESM.docx]

Review History

**First round of review**

**Reviewer 1**

**Were you able to assess all statistics in the manuscript, including the appropriateness of statistical tests used?**

Yes, and I have assessed the statistics in my report.

**Comments to author:**

Overview:

In the manuscript under consideration Fernandez et al describe a new software, 3DeFDR, that identifies differential looping events from 5C (and Hi-C) data sets. Importantly, their null model considers sample specific differences in distance-dependent interaction frequencies, sequence 'visibility', sequencing depth, and non-looping structures. Therefore, their method directly tests for significant differences in looping/loop strength rather than simply differences in interaction frequency. This is a key distinction from existing methods and an important aspect of 3DeFDR. To the best of my knowledge no distributed tool currently exists for this purpose. Since comparative looping studies are increasing in prevalence this tool is greatly needed. The authors describe the math and theory behind the software in great detail. The software controls the False Discovery Rate using a method with reasonable assumptions. The paper is well reasoned and well written and the data presented is convincing. Below are a few issues for consideration:

Installation, usage, and documentation:

Installation was fairly straightforward and worked well. The software is extensively documented and includes data as well as instructions to test the code. I actually received an error message when I tried to run the code itself; however, I cannot rule out the possibility that this was an error on my end. In general, though it appears that the authors put a great deal of effort into usability and I suspect if there are any issues they would be addressed quickly.

Issues:

1. (Major though outside the scope) I see that this paper was also submitted as a preprint to the preprint server bioRxiv. While it may be outside the scope of this review process I feel compelled to comment on that. I commend the authors for submitting a preprint describing their work. In my mind preprints serve two major purposes: (1) they benefit the scientific community by allowing rapid access to research and results and (2) they benefit the authors by allowing the researchers to get credit for their work in a timely fashion and avoid getting 'scooped'. However, both for the sake of the scientific community and for appropriate dispensation of credit it is of upmost importance that preprints are accompanied by all of the data/methods/code that are required of a peer reviewed publication. The preprint in question describes their software but does not provide access to the software itself! It therefore provides the scientific community no way to validate the claims made in the paper and is little use to the scientific community. In contrast these preprints are still used by the authors to establish precedence, show productivity on grants, etc. This practice defeats the entire purpose of preprints and drastically diminishes the value of preprints altogether. We suggest that the authors should post their code at their earliest convenience and rethink this practice in the future. If they are not comfortable sharing their code then they should just not submit a preprint.

2. (Minor) 3DeFDR appears to be a valuable tool for detecting significant differences in looping detected by 5C. This is fantastic and alone justifies its publication in Genome Biology. The authors also demonstrate is applicability to Hi-C data but as far as I could tell this was only applied to a small region of the Hi-C data. The authors should state whether or it can be applied to compare entire Hi-C data sets, how that effects run time, how many differential loops are detected, etc. That may be well beyond the scope of this paper. But if the authors could at least indicate to the reader if this is something they can apply to their Hi-C data in a straightforward way that would be greatly appreciated.

Conclusions:

There is a dire need for this type of software that explicitly tests for changes in 'looping' as opposed to changes in 'contact frequency'. The software was carefully designed and tested and is well described both in the manuscript and in software documentation. I recommend this manuscript for publication in Genome Biology with very minimal modification.

**Reviewer 2**

**Were you able to assess all statistics in the manuscript, including the appropriateness of statistical tests used?**

Yes: Examined methodology, including SI material

**Were you able to directly test the methods?**

No

**Comments to author:**

The study by Fernandez et al addresses an important problem in analysis of chromsome-conformation capture-based studies (e.g. 5C and potentially Hi-C), namely, identifying loop interactions that are separate from architectural features across different cell types. Authors present a novel method, 3DeFDR, for calling significantly differential 'loop interactions' in 5C data. Authors use standard bias correction techniques such as matrix balancing and then fit a logistic distribution over the observed-to-expected contact ratio to assign p-values at each (i,j) interaction pair. Each (i,j) p-value is then log-transformed and scaled to form an 'interaction score' (IS) comparable across multiple cellular conditions. The heart of the 3DeFDR algorithm is assignment of an empirical FDR based on the ratio: (number of significant null set loops) : (number of significant experimental set loops), for a given IS threshold difference and looping class. The null set is generated by modeling each (i,j) count as a negative binomial distribution with mean at the experimental mean (i,j) count and with variance a quadratic function of that mean; contact matrices are then generated from these negative binomial distributions to form the (6) replicates constituting the null set. The authors compare the 3DeFDR method to 3 other methods: ANOVA, a z-score based likelihood ratio test (LRT), and diffHic.

There are a number of concerns with this manuscript:

1. The describe method is built upon several well-known algorithms, including quantile normlization, and matrix balancing. There are known issues with these procedures, making these existing methods difficult to use when comparing different cell types. For example, matrix balancing renders any contact matrix into essentially double-stochastic matrix, therefore cannot detect the different degree of overall interactions between two cell types, one has overall stronger interactions and one less so, such as well-documented effects of nucleus size changes, which has enormous effects on overall interaction reads. The authors work inherits all these problems as well.

2. The generation of replicas is based on negative binomial distribution from independent Bernoulli trials. There are a number of issues. First, as pointed out by the authors, the number of replica (=2) is insufficient for estimation of parameters of the binomial distributions. The authors' solution is to generate simulated replica following some probabilistic models. To construct such models, however, the authors stratified all genomic regions by genomic distance. This essentially makes the assumption of independent Bernoulli trial, with parameters derived from averaged pooled data.

This is highly problematic when one is trying to determine change point-type of rare events (forming cell-condition specific loops) that is strongly specific to individual locus (hence the well-known local donut correction), as the averaged null model may be very different from a proper null model for that specific region when there is a large number of replicates. Furthermore, the mean-variance relationship is assumed to be quadratic. This again has not been adequately justified.

In addition, the authors removed anything above 2.5 standard deviation, as well as low counts (suggesting even pooled stratfieid data is insufficient). These heavily massaged data are guaranteed to generate mild and nice simulated data points, but at great cost of distorting the real physical phenomena.

In fact, I am puzzled. The pseudoreplicates are generated with an assumed model, therefore an empriical derived parametric model is already available. If the authors are very confident that these null models are valid, why not doing the logically obvious and directly obtain p-value from the negative binomial distributions and calculate a simple FDR using p-value of these null distribution whose analytical form is known from the estimated parameters?

3. The approach of pooling bin-bin interactions of similar genomic distance together is also problematic in another regard, as this equalizes potentially strong heterogeneity in interactions at different genomic locations.

4. The fundamental importance of nuclear confinement on the overall folding landscape of chromosomes - it is so important that even scaling is different. Hence, it would be difficult to compare 5C/Hi-C across different cell types without explicit considerations of differences in nuclear volume. The authors should compare their approach with another method for loop interactions of 5C data that does take volume confinement into consideration https://doi.org/10.1093/nar/gkx784

Overall, this is an interesting study attempting to address an important problem. The authors made some decision in modeling random replicates in an ad hoc fashion. However, the method is largely built upon application of a series of processing tools, and inheriting many of the problems there as well. Admittedly, there are many issues in existing methods as well. However, the proposed new method does not provide solution to these key issues. The authors is attempting to find some ad hoc solutions to important problems, whose answers is currently unknowable - the authors rightly pointed out that no data of large biological replica exist. With this caveat, the overall value of the describe work is uncertain. Sans adequate number of biological replicates, the effectiveness and improvement of this study are unknowable, as there are no true ground truth due to lack of biological replicas.

Other issues:

5 There are many ad hoc tuning parameters that are left to user choice with little guidance or specifics towards a rational means of selecting specific values.

5i) A major concern is the choice of biological condition to serve as the null set. The supplement (page 8) notes that there are 6 biological for 3 (A, B, C) conditions; however it is up to the user to choose which of the conditions (A, B, or C) serves as the null set. How sensitive is the choice of condition to the observed differential loop results? Would it perhaps make more sense to separately use each condition as the null set and then take the consensus among all null conditions? Or perhaps a Bayesian approach using a uniform prior over the conditions?

5ii) Another choice is the background cutoff for determining if a loop should be tested for differential interactions. From SI p9, a p-value of 0.8 across all replicates is required before considering a loop for testing. How sensitive are the results (in terms of calling cell type specific loops) to this background threshold? This background threshold ultimately reduces the number of tests performed and therefore can make your approach more powerful. Is this ad hoc background filtering applied to all other methods (ANOVA, LRT, diffHic)? If not, how similar or different are the differential loops when background filtering is applied to all methods?

5iii) What are the justifications for the various distance regimes and associated smoothing windows? Are these also tune-able by the user? Again, how sensitive are the final differential loop results to the specific choose of distance regimes and windows?

6. SI p.8. It seems the number of null replica shows up as above threshold is important for eFDR, but why the authors a priori choose the number of 6 replica? The authors should also specify how sensitive are the results to then number of replicates. As they are simulated, their generation should of very low cost. Thousands to even hundreds of thousands would be appropriate.

7.It is unclear if the donut filter was applied to pre-process the diffHic data. If not, then that could explain why diffHic was not specific for loops relative to 3DeFDR - in which case, the comparison made was then not a fair comparison.

8. In the submission letter, corresponding authors mentions a "gold standard" set of loop interactions. However, there does not appear to be any mention of such a set in the main text, nor are there any standard comparisons against such a set (e.g. sensitivity, specificity, precision, recall, accuracy, AUCROC, AUCPR, etc). Is there actually a putative gold standard set of loops? If so, where are these comparisons against the "gold standard" set?

9.The approach of emperical FDR estimation method is not novel. A well-known example is that of SAM (Significance Analysis of Microarrays): http://statweb.stanford.edu/~tibs/SAM/, which used bootstrap permutations to estimate FDR. The authors should acknowledge such earlier work.

Minor Issues:

10. p.8 l.21-24. A citation here is needed.

11. The statement on p. 8 that line 55 that "all confounders" have been corrected is an overstatement and needs to be modified.

12. The assumption of log-logistic distribution, and hence p-value are also not justified.

**Authors Response**

**Point-by-point responses to the reviewers’ comments:**

Reviewers’ Comments

Reviewer #1

Overview: In the manuscript under consideration Fernandez et al describe a new software, 3DeFDR, that identifies differential looping events from 5C (and Hi-C) data sets. Importantly, their null model considers sample specific differences in distance-dependent interaction frequencies, sequence 'visibility', sequencing depth, and non-looping structures. Therefore, their method directly tests for significant differences in looping/loop strength rather than simply differences in interaction frequency. This is a key distinction from existing methods and an important aspect of 3DeFDR. To the best of my knowledge no distributed tool currently exists for this purpose. Since comparative looping studies are increasing in prevalence this tool is greatly needed. The authors describe the math and theory behind the software in great detail. The software controls the False Discovery Rate using a method with reasonable assumptions. The paper is well reasoned and well written and the data presented is convincing. Below are a few issues for consideration:

Installation, usage, and documentation: Installation was fairly straightforward and worked well. The software is extensively documented and includes data as well as instructions to test the code. I actually received an error message when I tried to run the code itself; however, I cannot rule out the possibility that this was an error on my end. In general, though it appears that the authors put a great deal of effort into usability and I suspect if there are any issues they would be addressed quickly.

*Author response 1.1*

*We want to do everything in our power to make our code usable and accessible to scientists across a range of disciplines. We have updated the installation instructions so that 3DeFDR can be run within a containerized Docker environment. Docker is known for its ease of use and circumvents the problem of extensively testing applications to ensure they work across a wide range of operating systems and platforms by creating a completely self-contained environment that can be used effectively on any system. We will also provide the original documentation and instructions for installation with just Conda and pip for individuals who prefer not to use Docker.*

Issues:

1.	(Major though outside the scope) I see that this paper was also submitted as a preprint to the preprint server bioRxiv. While it may be outside the scope of this review process I feel compelled to comment on that. I commend the authors for submitting a preprint describing their work. In my mind preprints serve two purposes: (1) they benefit the scientific community by allowing rapid access to research and results and (2) they benefit the authors by allowing the researchers to get credit for their work in a timely fashion and avoid getting 'scooped'. However, both for the sake of the scientific community and for appropriate dispensation of credit it is of upmost importance that preprints are accompanied by all of the data/methods/code that are required of a peer reviewed publication. The preprint in question describes their software but does not provide access to the software itself! It provides the community no way to validate the claims made in the paper and is little use to the scientific community. In contrast these preprints are still used by the authors to establish precedence, show productivity on grants, etc. This practice defeats the entire purpose of preprints and drastically diminishes the value of preprints altogether. We suggest that the authors should post their code at their earliest convenience and rethink this practice in the future. If they are not comfortable sharing their code then they should just not submit a preprint.

*Author response 1.2*

*We have carefully read the reviewer’s point and will keep this top of mind for future manuscripts. In the specific case of the first submission, one of the authors expressed that it was highly important to them to get reviewer feedback on our statistical framework before releasing code and I wanted to honor the request. For this resubmission, we have made both 3DeFDR-5C and 3DeFDR-HiC publicly available:*

*https://bitbucket.org/creminslab/3defdr-hic*

*https://bitbucket.org/creminslab/3defdr-5c*

2.	(Minor) 3DeFDR appears to be a valuable tool for detecting significant differences in looping detected by 5C. This is fantastic and alone justifies its publication in Genome Biology. The authors also demonstrate is applicability to Hi-C data but as far as I could tell this was only applied to a small region of the Hi-C data. The authors should state whether or it can be applied to compare entire Hi-C data sets, how that effects run time, how many differential loops are detected, etc. That may be well beyond the scope of this paper. But if the authors could at least indicate to the reader if this is something they can apply to their Hi-C data in a straightforward way that would be greatly appreciated.

*Author response 1.3*

*We have now developed a completely new variant of 3DeFDR, 3DeFDR-HiC, to enable 3DeFDR to call differential loops on Hi-C data genome-wide in a reasonable time frame. To reflect this addition, we have updated the manuscript, Figures 6 and 7, and Supplementary Figures 12-14. Now 3DeFDR will have utility for calling differential loops on two conditions for Hi-C data as well.*

Conclusions: There is a dire need for this type of software that explicitly tests for changes in 'looping' as opposed to changes in 'contact frequency'. The software was carefully designed and tested and is well described both in the manuscript and in software documentation. I recommend this manuscript for publication in Genome Biology with very minimal modification.

*Author response 1.4*

*We thank the reviewer for their enthusiasm for the usefulness of our tool.*

Review #2

The study by Fernandez et al addresses an important problem in analysis of chromosome-conformation capture-based studies (e.g. 5C and potentially Hi-C), namely, identifying loop interactions that are separate from architectural features across different cell types. Authors present a novel method, 3DeFDR, for calling significantly differential 'loop interactions' in 5C data. Authors use standard bias correction techniques such as matrix balancing and then fit a logistic distribution over the observed-to-expected contact ratio to assign p-values at each (i,j) interaction pair. Each (i,j) p-value is then log-transformed and scaled to form an 'interaction score' (IS) comparable across multiple cellular conditions. The heart of the 3DeFDR algorithm is assignment of an empirical FDR based on the ratio: (number of significant null set loops) : (number of significant experimental set loops), for a given IS threshold difference and looping class. The null set is generated by modeling each (i,j) count as a negative binomial distribution with mean at the experimental mean (i,j) count and with variance a quadratic function of that mean; contact matrices are then generated from these negative binomial distributions to form the (6) replicates constituting the null set. The authors compare the 3DeFDR method to 3 other methods: ANOVA, a z-score based likelihood ratio test (LRT), and diffHic.

There are a number of concerns with this manuscript:

1.	The describe method is built upon several well-known algorithms, including quantile normalization, and matrix balancing. There are known issues with these procedures, making these existing methods difficult to use when comparing different cell types. For example, matrix balancing renders any contact matrix into essentially double-stochastic matrix, therefore cannot detect the different degree of overall interactions between two cell types, one has overall stronger interactions and one less so, such as well-documented effects of nucleus size changes, which has enormous effects on overall interaction reads. The authors work inherits all these problems as well.

*Author Comment 2.1*

*We agree with Reviewer 2 that the question of how to analyze genomics data for biological cases where there is a global change in nuclear volume or a global change in transcriptional amplification remains open and difficult to solve. Some investigators have approached this problem experimentally by adding spike-in controls from another species, and to our knowledge this has thus far been the most effective approach to control for global effects. For the 3D genome folding field, this problem has not been tackled computationally in published work to our knowledge – and it would be a stand-alone paper.*

*In our resubmission, we introduce a new tool, 3DeFDR-HiC, that tests raw contact frequencies while taking sequencing depth differences between replicates and bias factors per pixel into account in the model. 3DeFDR-HiC was built using many of Reviewer 2’s exciting ideas (his/her subsequent critiques below) and gives users the ability to now test loops genome-wide from Hi-C data, not just 5C. For Hi-C data, we do not test matrix balanced counts for differential looping but instead use matrix bias vectors and sequencing depth scaling factors in our model of differential interaction frequency across conditions. To facilitate the highest level of flexibility to adapt our method to a specific biological question, we allow the bias factor and scaling factor parameters to be modified or removed by the user. Thus, the code is set up to allow the discretion of the user to retain or remove replicate wide or condition wide shifts in interaction strength based on their specific biological question. As debates continue on whether or not nuclear volume affects looping strength and whether or not any specific looping interaction is significantly differential among biological conditions, 3DeFDR-HiC is now more suited to adapt to the assumptions in the future.*

*We have devoted nearly 1 year in revision to develop and extensively test 3DeFDR-Hi-C. For the case of nuclear volume questions, we believe that solving this specific problem computationally is far outside the scope of our current paper, which only claims to provide statistical and computational tools to identify cell type specific loops in 5C and Hi-C data. We have added this point to the usage instructions and added a new paragraph in the discussion openly sharing the limitations of our methods, including that they cannot in their current form detect global changes in genome folding due to nuclear volume.*

2.	The generation of replicas is based on negative binomial distribution from independent Bernoulli trials. There are a number of issues. First, as pointed out by the authors, the number of replica (=2) is insufficient for estimation of parameters of the binomial distributions. The authors' solution is to generate simulated replica following some probabilistic models. To construct such models, however, the authors stratified all genomic regions by genomic distance. This essentially makes the assumption of independent Bernoulli trial, with parameters derived from averaged pooled data.

This is highly problematic when one is trying to determine change point-type of rare events (forming cell-condition specific loops) that is strongly specific to individual locus (hence the well-known local donut correction), as the averaged null model may be very different from a proper null model for that specific region when there is a large number of replicates.

*Author Comment 2.2*

*Reviewer 2 raises a very important issue that is broadly important for the entire field of genomics – namely how to precisely estimate variance for any given gene, nucleotide, or (in this specific case) bin-bin interaction frequency when the creation of replicates is expensive and labor-intensive. This problem is more severe in studies of the 3D genome than in, for example, RNA-seq because a single high resolution Hi-C data set can require 1-3 billion reads, which is quite refractory to the creation of high numbers of high-quality replicates.*

*We are on the same page with Reviewer 2 - we agree that variance estimation would be more precise when done on 10s to 100s of replicates – thus allowing for variance estimation on a per-pixel basis and consideration of the local folding environment (local TAD/SubTAD, compartment, distance dependence for any specific genomic locus) for every pixel individually. As we imagine the Reviewer appreciates, it is not possible for most labs to create more than two Hi-C replicates per biological condition (average cost $20,000 per replicate for loop resolution Hi-C), so a worthy endeavor for progress is to find ways to pool the data to make first steps toward estimates, which can in turn become more accurate or precise with numerous iterations as the field and statistical ideas develop for this particular type of data. We have specifically added the Reviewer’s point to our Discussion to ensure it is clear to the readers that our work takes the first steps toward the goal of precise variance estimation.*

*We did have one very important point for clarification. Regarding the parameterization of our negative binomial models: while we do use a pooling strategy to estimate the dispersion parameter, we do not use any pooling when estimating the mean parameter. Specifically, we use the sample mean of the normalized interaction values across the two replicates for each pixel to obtain an independent mean parameter estimate for each pixel. This results in the construction of null models which are different at every pixel and which adapt to the vast diversity of genomic structures (loops, domains, etc.) in a context-specific way. Thus, our parameter estimates are not solely due to grouped data. However, our choice to pool information from multiple pixels when estimating the dispersion parameter still requires additional justification.*

*Reviewer 2’s first important point is that when we look at MVR across different distance scales, at each distance there are many points coming from all manner of local TAD/subTAD structures, which ultimately leads to an average MVR that might not be the best estimate of the MVR at each specific genomic context.*

*At the outset of our study design, we reviewed the RNA-seq literature (in particular the statistics papers that formed the intellectual basis for DESeq1/2 and EdgeR). Based on these studies, we made the critical assumption that simple sample variance across n=2 replicates for every 5C/Hi-C pixel would result in noisy variance estimates, and that noise could be overcome by pooling the data and creating a model for the Mean-Variance-Relationship as done in EdgeR/DESeq2. Specifically, we can see from the leading RNA-seq analysis methods that they rely on the assumption that points at the same mean can be pooled together for variance estimation in the absence of many replicates. We also assumed that our data would be overdispersed and that a Poisson model where mean=variance would result in flawed loop calls. We assumed we needed to use the negative binomial distribution to model our counts, as is done in a very large proportion of genomics data papers across most genomics technologies.*

*As we will show below, our careful assessment over the last year to address Reviewer 2’s feedback is that, surprisingly, for 5C data, the simple sample variance performs similar (but still not as well) as several pooling models. Therefore, there is justification that pooling allows for improvement in either sensitivity or specificity of loop calls, but that does not bring a dramatic improvement over simple n=2 sample variance estimates. Moreover, importantly, multiple options for pooling generally result in the same result, and do not significantly alter the performance of the differential loop calling approaches. Therefore, generally speaking, while pursuit of pooling alternatives is intellectually interesting and requires more work in the future, our work thus far shows that various pooling approaches do not bring about major improvements to accuracy or sensitivity of loop calls.*

*With that general point being made, we would like to emphasize, however, that there is one length scale, where our pooling variance estimation approach does provide an advantage. Specifically, 3DeFDR-5C outperforms local pooling and sample variance estimates for loops at short distance scales (high mean regimes). Moreover, we also observe that for Hi-C data, precise variance estimation via pooling does indeed lead to an advantage in performance for the pooling approach used in 3DeFDR-HiC compared to sample variance and several other methods – with the advantage in performance shown most distinctly at short distance scales (high mean regimes). Below we detail our findings for both 5C and Hi-C data.*

*First, we performed additional analyses to explore the Reviewer’s ideas more deeply (Figure 1 below). We compared three approaches for estimating per-pixel variance in 5C data. First, we estimate per-pixel variance estimated across n=2 replicates per condition (in this case NPCs) using the normal sample variance equation (points in blue, Figure 1).*

*Second, using the same data, and based on the Reviewer’s suggestion, we implemented a local genome context-specific pooling approach. In this approach, we first compute per-pixel variance estimates across n=2 replicates per condition and then apply a Gaussian filter, with a four-pixel radius, to every pixel, resulting in a “local neighborhood” variance estimate for each pixel (points in purple, Rebuttal Figure 1 above). This Gaussian filter is implemented using astropy.convolution.convolve to take advantage of its special handling of NaN values, which involves replacing NaN values with interpolated values during the convolution calculation to avoid large NaN holes while also propagating NaN values present prior to convolution through to the final output. Thus, this “local” pooled variance estimate is an implementation that might represent a local estimate of variance as described by the Reviewer thus represent weighted averages of the per-pixel sample variances from nearby pixels.*

*Third, in the approach used in 3DeFDR-5C, we group points by distance scale and estimate dispersion by fitting a mean-variance relationship (points in red, Rebuttal Figure 1). As the Reviewer already alluded to, we see that both variance and dispersion estimates are extremely noisy across all distance scales in the case where no pooling (Approach 1) was used (blue, Rebuttal Figure 1A-C). As expected, we see that local pooling (Approach 2) attenuates the variance and dispersion dynamic range compared to no pooling (purple, Rebuttal Figure 1A-C). By the nature of our method, the 3DeFDR-5C distance-based pooling approach performed on 5C data (red) gives a tight estimate of dispersion that increases as a function of genomic distance as we described in our manuscript (Rebuttal Figure 1B-C). Importantly, we also see the same pattern in Hi-C data (Rebuttal Figure 2), suggesting that dispersion increase as a function of increasing distance is a general feature of ‘C’ data (Rebuttal Figure 2). These data indicated to us that 5C and Hi-C data are indeed overdispersed, which suggested to us that we should not use Poisson statistical assumption of mean=variance and instead rely on Negative Binomial. Our data also suggested that dispersion might increase as a function of distance in both 5C and Hi-C data, which is intuitive given that interaction frequency mean decreases as a function of distance. Therefore, we used this information (Rebuttal Figures 1+2) to justify pursuing a distance-variance-relationship for our variance estimation, which we will now below compare below to the sample variance and local pooling approaches for an assessment of looping performance.*

*Now that we have justified the basis for some of our assumptions (i.e. overdispersion, distance-variance-relationship, negative binomial) – the most important next step is to directly compare how each of these three dispersion estimation approaches might influence loop detection (Rebuttal Figure 3 ). What we ultimately observe in 5C data is that the loop identification was not changed in any major way with the various methods. A priori, we expected that the approach with no pooling would result in major alterations to false positive and false negative loop calls. In reality, across all genomic regions in the 5C data set, including the three regions shown below, we see globally similar looping landscapes (Rebuttal Figure 3). In the figure we show eFDR threshold of 2%, but the results are similar across all thresholds.*

*It is quite important to point out that we did observe one critically important difference between methods – our 3DeFDR variance pooling approach was more sensitive in detecting NPC-specific interactions at short distance scales where the mean and the variance are the highest (see numerous loops demarcated by green arrows exclusively identified by 3DeFDR in Sox2, Olig, and Klf4 regions in Rebuttal Figure 3 below) compared to the dispersion estimates of the non-pooling and local-pooling (Gaussian filter) approaches. On the basis of this performance advantage, we used this data to guide our decision to stick with our current pooling strategy for 3DeFDR-5C.*

*From this observation, we began to hypothesize that the variance estimation might matter the most for loops that fall in short distance scales (0-400 kb) because these are the locations with the highest mean expectation and the highest variance in both 5C and Hi-C data (Rebuttal Figure 4).*

*We next turned to Hi-C data to determine if we could find any evidence of a difference in performance advantage dependent on distance. Specifically, we spent the last year formulating 3DeFDR-HiC by utilizing some of the Reviewer’s suggestions in subsequent comments. We also created simulations of Hi-C data, which then allowed us to know the exact placement of true positive loops, thus opening up the opportunity to compute false positives and false negatives and plot ROC curves. This analysis is detailed fully in the revision, new Figures, and extensively revised supplement. In brief, what we observe is consistent with 5C data. Specifically, we see that the 3DeFDR pooling approach outperforms the use of a standard sample variance – and the performance advantage is more notable at short distances for the pooling approach vs. the n=2 sample variance approach. Although we did not test a local pooling strategy here, it is clear that variance estimation through some form of pooling assists in sensitive and specific loop calling, with a stronger advantage at short versus long distance scales, in both Hi-C and 5C data (Rebuttal Figure 5).*

*For the reasons described above, we decided we were justified in keeping our original pooling approach, in particular given that 3DeFDR-5C was the most sensitive approach for short distances with general parity among all three options. We add this point as a critical key learning to the discussion. Our working hypothesis is that in short distance, high mean expectation contact regimes, the accuracy of estimating the variance within a large dynamic range of points will be extremely important, and accurate variance estimation in the distance regime where the variance is highest might aid in the sensitivity and specificity of loop calling within short-distance scale regimes. We hope that our team and others in the field will continue to explore the problem of variance/dispersion estimation in great detail and test this and other hypotheses in the future. We also emphasized in the discussion that variance estimation methods for Hi-C remains an open area for future improvement to ensure our claims are carefully worded.*

*Overall, what we have learned is that for ‘C’ data the variance estimation is not as globally important for differential loop calling as it is for differential gene expression analyses in RNA-seq. Our hypothesis for this discrepancy is that RNA-seq data has a much higher dynamic range of counts than ‘C’ data, and the over dispersion estimates matter the most for the very highly expressed genes. Importantly, consistent with this idea, we do indeed observe that our methods (3DeFDR-5C and 3DeFDR-HiC) are strong in the case of ultra-short range loops of high mean, high variance, and short distance scales. We think this is an intellectual contribution and major source of learning from our paper that will benefit the entire 3D genome folding field. We have also added this point to the Discussion in this revision.*

*Finally, we point out that in addition to the data we have shown, we have worked to ensure that 3DeFDR-5C and -HiC code presents a wide range of options for how model parameters are fitted to the input counts data. We ensure that the usage instructions carefully outline the possibilities so that the readers can use our tools to explore future ideas. For example, model parameters may be fit in global or region specific manner by setting a regional flag.*

Furthermore, the mean-variance relationship is assumed to be quadratic. This again has not been adequately justified.

Author Comment 2.3

*The Reviewer raises another important point that we have not adequately justified the decision to pursue a quadratic mean-variance relationship. Here, we provide additional explanation and justification for our use of the negative binomial distribution for describing the 5C and Hi-C data in our analyses.*

*First, some basic assumptions: We consistently observe that Hi-C (Rebuttal Figure 2 ) and 5C data are over-dispersed (Rebuttal Figure 6B+C) and that this overdispersion is present at all distance scales and across all regions. We also clearly see that overdispersion varies as a function of genomic distance in both 5C and Hi-C data (Rebuttal Figure 2, Rebuttal Figure 6B+C). We in turn assumed that our discrete counts could be accurately modeled with a discrete model for overdispersed counts, therefore we selected the negative binomial distribution for our counts generating functions and our 3DeFDR-HiC statistical test (new to this revision).*

*Specifically, for our analysis of both 5C and Hi-C data, we assumed on the basis of our observations of over-dispersion in the data that the data could be described by negative binomial distributions with a specific variance at each distance scale and for each condition. Under these assumptions, the variance of each negative binomial distribution NB# %&'() , ,&-./01(2 parameterized in terms of its per-pixel mean %&'() and per-distance*

*dispersion	,&-./01( is Var7NB# %&'(), ,&-./01(28 = %&'() + ,&-./01( × %&'()<, revealing the*

*source of our quadratic mean-variance relationship model.*

*Now, to the core of the Reviewer’s question: For 5C data, our quadratic model (which follows from the property of the negative binomial distribution described above) appears to fit the shape of the data reasonably well, as assessed by R2 values (Rebuttal Figure 6D). Empirically our data support our use of the quadratic fit – and our ultimate quality of loop calls indicate this is a fair assumption. We have also added a statement to the discussion clarifying that a better fit than quadratic (equivalently, a distribution better than negative binomial) may be uncovered in future work, which is a very fair point.*

In addition, the authors removed anything above 2.5 standard deviation, as well as low counts (suggesting even pooled stratified data is insufficient). These heavily massaged data are guaranteed to generate mild and nice simulated data points, but at great cost of distorting the real physical phenomena.

*Author response 2.4*

*We understand the Reviewer’s perspective. We explored our data to understand how the outlier filter was influencing our results. We show below (Rebuttal Figure 7) that while different types of filters might influence the R2 value of our model fits, the dispersion estimates are largely unchanged by outlier removal. Notably, the dispersion estimates remain nearly exactly the same, regardless of the filter, at the short distance scales where we have shown in early Rebuttal Figures that our extra steps to model variance matter for loop calling.*

*The filter steps we pursued are as follows: We applied two filters to raw 5C counts prior to fitting the negative binomial mean-variance relationship per distance scale bin such that these points are ignored in the process of fitting dispersion parameters: (1) a filter that drops points with zero variance across replicates of a given condition, and (2) a filter that drops points with extremely high counts, having a mean value across replicates that is 2.5 STDs higher than the mean value for points in a given distance scale bin. Filter (1) is implemented because we find it useless to fit a counts generating function to a point that has exactly the same counts in all replicates. Filter (2) is implemented because we found that the negative binomial MVR was susceptible to a very small number of extreme outliers.*

*We observe (Rebuttal Figure 7) that the regional negative bionomial MVR fittings of Sox2 and Olig1-Olig2 and the global MVR fittings (all regions) have high R2 values without any filters, but then removal of ultra-high outliers significantly lowers the R2 values. This indicates that the fits appear stronger simply because the outliers are driving the R2. Importantly, despite the reduction of R2 with the outlier removal, we see the dispersion estimates are largely unchanged, showing only a mild increase at exclusively long distances. This finding is important because we think it suggests that the dispersions will not change in a meaningful way by adding back outlier, but the R2 would be artificially increased by keeping outliers in. Therefore the loop calls will not change in a meaningful way regardless of whether or not the R2 values are altered by the presence of outliers. We now note these points in our Supplementary Methods and include description in our software usage instructions to ensure the users are aware they can remove or change these filters as they see fit in their own analyses.*

In fact, I am puzzled. The pseudoreplicates are generated with an assumed model, therefore an empirical derived parametric model is already available. If the authors are very confident that these null models are valid, why not doing the logically obvious and directly obtain p-value from the negative binomial distributions and calculate a simple FDR using p-value of these null distribution whose analytical form is known from the estimated parameters?

*Author Comment 2.5*

*Thank you for pointing us to this extremely important point. In fact, after discussing this feedback in great detail, we realized we could formulate a likelihood ratio test that would be much faster for Hi-C loop calling genome-wide without the need for simulations. Over the past ten+ months, we have created 3DeFDR-HiC – a new statistical framework that relies on the Reviewer’s idea and is very useful for calling loops in Hi-C data without the need for simulations that are too compute- and time-intensive. Now 3DeFDR has variants effective for calling loops in both 5C and Hi-C data. We show the output in new main Figures 6 and Figure 7 and we provide the statistical framework and variance estimation in the Supplementary Methods and Supplementary Figures 12-14.*

3.	The approach of pooling bin-bin interactions of similar genomic distance together is also problematic in another regard, as this equalizes potentially strong heterogeneity in interactions at different genomic locations.

*Author Comment 2.6*

*We thought deeply about this critique and conducted an extensive analysis in Author Comment 2.2, including the addition of a local variance pooling window to address the concern about heterogeneity at each distance scale. As detailed in that comment (Rebuttal Figures 1-7), the approach used in 3DeFDR-5C and 3DeFDR-HiC gives us sensitivity for loop detection at short distance scales compared to the local pooling approach.*

4.	The fundamental importance of nuclear confinement on the overall folding landscape of chromosomes - it is so important that even scaling is different. Hence, it would be difficult to compare 5C/Hi-C across different cell types without explicit considerations of differences in nuclear volume. The authors should compare their approach with another method for loop interactions of 5C data that does take volume confinement into consideration https://doi.org/10.1093/nar/gkx784

*Author Comment 2.7*

*The paper referenced calls loops in a single condition, but is not a differential loop caller. We invested a huge amount of effort (> 10 months) in building 3DeFDR-HiC and addressing the Reviewers other critical points. We think that comparing our work to a method that is not capable of calling differential loops is out of the scope of our work, but we also add points to Discussion and usage instructions so that readers are aware of how to adjust parameters as they wish for their biological question.*

Overall, this is an interesting study attempting to address an important problem. The authors made some decision in modeling random replicates in an ad hoc fashion. However, the method is largely built upon application of a series of processing tools, and inheriting many of the problems there as well. Admittedly, there are many issues in existing methods as well. However, the proposed new method does not provide solution to these key issues. The authors is attempting to find some ad hoc solutions to important problems, whose answers is currently unknowable – the authors rightly pointed out that no data of large biological replica exist. With this caveat, the overall value of the describe work is uncertain. Sans adequate number of biological replicates, the effectiveness and improvement of this study are unknowable, as there are no true ground truth due to lack of biological replicas.

*Author Comment 2.8*

*We humbly state that we have a different perspective than the Reviewer on this point. The goal of 3DeFDR-5C is to provide the first freely available and effective statistical tool for identifying cell type-specific looping interactions in 5C data. We also spent the last 9 months developing a Hi-C version based on the Reviewers suggestions above, thus we now provide an extensively newly developed 3DeFDR-HiC method to call cell-type-specific loops in Hi-C data genome-wide.*

*The goal of 3DeFDR is to identify cell type specific loops – and we demonstrate extensively with seven main figures and extensive supplementary figures that the method is highly effective in calling cell type-specific loops. The cell type-specific loops we identify exhibit strong enrichment for the expected cell type-specific chromatin modifications. We think it is unfair to suggest that our statistical assumptions are incorrect given that they effectively capture most cell-type-specific loops visible in the heatmaps. As the Reviewer likely experiences in his/her own research, new methods require new ideas, frameworks, and code, and, even in the absence of perfection, new knowledge can be gained and new ground can be broken. We do not claim that our assumptions are perfect, but we do think they are a great first start toward enabling the 3D genome folding community to statistically evaluate cell type-specific loops in *both* Hi-C and 5C data. Moreover, most if not all, genomics fields also rely on mean-variance-relationships and pooling of data for estimations of variance in the absence of a sufficient number of replicates, e.g. the highly utilized and highly effective EdgeR and DEseq software packages. We view these as setting the precedent for this manner of pooling and in turn providing great value to the genomics community.*

*Finally, we add a new Figure 7 and new Supplementary Figures to the main resubmission which provides the Reviewer requested quantitative estimates of sensitivity and specificity for loop calls from 3DeFDR-HiC. Specifically, we provide these values in the form of an ROC curve for 3DeFDR-HiC on Hi-C simulations containing known differential and constitutive loops. In this way, we estimate the performance of 3DeFDR in the absence of a true gold standard.*

Other issues:

5)	There are many ad hoc tuning parameters that are left to user choice with little guidance or specifics towards a rational means of selecting specific values.

*Author Comment 2.9*

*We provide an extensive user guide for both 3DeFDR-5C and -HiC. We have expanded this guide to ensure that we communicate all loop calling parameters available for tuning by the user and we ensure that the parameters are customizable with our freely available code.*

5i) A major concern is the choice of biological condition to serve as the null set. The supplement (page 8) notes that there are 6 biological for 3 (A, B, C) conditions; however it is up to the user to choose which of the conditions (A, B, or C) serves as the null set. How sensitive is the choice of condition to the observed differential loop results? Would it perhaps make more sense to separately use each condition as the null set and then take the consensus among all null conditions? Or perhaps a Bayesian approach using a uniform prior over the conditions?

*Author Comment 2.10*

*To thoroughly address this Reviewer comment, we worked to assess the possible sensitivity of 3DeFDR-5C empirical FDR estimates to parameters of the null replicate set, namely (1) choice of biological condition from which to model and generate the null replicate set, and (2) choice of the total number of null replicate sets over which to average the final eFDR estimate. In Rebuttal Figure 8 below, we provide FDR estimate distributions, determined by 3DeFDR-5C, for (1) for each possible choice of biological condition (ES-2i, ES-serum, or NPC) and (2) a sample of possible differential loop classifications (ES-2i-only, ES-Serum-only, NPC-only, and ES-2i-and-ES-Serum-specific). These distributions are determined by repeatedly generating null replicate sets of differing total size (2, 5, 10, 20, 40, 60, 80, and 100 sets), computing an FDR estimate for each, and plotting them as histograms.*

*We found that eFDR estimates by 3DeFDR-5C exhibited only slight and very minor shifts when using different null conditions (Rebuttal Figure 8 ) We also note that the central tendency of the eFDR from our simulation sets varies only minorly as the number of independent simulations grows (Rebuttal Figure 8 ). Given that Rebuttal Figures 1-7 demonstrate that slight alterations in dispersions have a negligible effect on loop sensitivity, together this data indicates to use that our choice of using N=250 simulated null replicate of containing six replicates each, generated from a model of the input biological condition, specifically the condition that will result in the most conservative eFDR estimates, will be sufficient for creating a valid null model upon which to accurately compute the eFDR.*

*For future experiments in the scientific community, it is important to note that some biological conditions may on average have significantly higher dispersion estimates than others. We formatted our code so that the user can explore the dispersion values across conditions and distance scales. We note in the discussion that choosing a condition with higher dispersion estimates could result in a set with a higher dynamic range of counts, and in turn a higher estimated false positive rate and higher eFDR estimate. Thus, we recommend that the user plot DVR fittings and determine which input condition or average of conditions would result in the most conservative eFDR estimates, and, in turn, the most conservative differential loop calls. We note that we ensure that the code itself allows the user to generate loop calls for each possible choice of null condition and then compute a consensus across those result sets (detailed in the usage instructions). Although we added this functionality, we do instead recommend choosing the null condition as that with the highest replicate-to-replicate variability to result in the most conservative empirical FDR estimates.*

5ii) Another choice is the background cutoff for determining if a loop should be tested for differential interactions. From SI p9, a p-value of 0.8 across all replicates is required before considering a loop for testing. How sensitive are the results (in terms of calling cell type specific loops) to this background threshold?

*Author Comment 2.11*

*To clarify, the purpose of the background threshold is solely to identify pixels that we deem as “background non-looping bin-bin pairs”. This threshold has absolutely no influence over constitutive and differential loop calls. We use these background loop calls only to compute enrichment of cell-type-specific loop classes above background at cell-type-specific genes, enhancers, and CTCF sites, and similarly to compute enrichment of constitutive loop classes above background at constitutive genes, enhancers, and CTCF sites. As shown in Rebuttal Figure 9 below, computed enrichments on the Beagan et al. 2017 5C data set are relatively insensitive to the choice of background threshold. However, to best address this Reviewer comment, we highlight in our usage instructions that this background threshold is highly dependent on read depth and complexity of the input data and so we recommend that each user optimize this threshold for each experiment.*

This background threshold ultimately reduces the number of tests performed and therefore can make your approach more powerful. Is this ad hoc background filtering applied to all other methods (ANOVA, LRT, diffHic)? If not, how similar or different are the differential loops when background filtering is applied to all methods?

*Author Comment 2.12*

*As stated in Author Comment 2.11, we chose background threshold to enable the enrichment testing of chromatin marks in cell type-specific loops. We do use the same background loops for testing enrichments of chromatin marks across all looping classes to ensure fair comparison. However, we would like to emphasize again that background thresholding has absolutely no meaningful effect on the number of loops called nor their cell type-specific designation.*

5iii) What are the justifications for the various distance regimes and associated smoothing windows? Are these also tune-able by the user? Again, how sensitive are the final differential loop results to the specific choose of distance regimes and windows?

*Author Comment 2.13*

*We extensively discuss this topic with data and text in Author Response 2.2. We show that dispersion varies as a function of distance, and that without this stratification, we over- or under-estimate the dispersion, in particular at short length scales, which ultimately leads to false negative loops specifically at short distance scales (Rebuttal Figures 1-7). Below we also show a plot of the final dispersion estimates vs. distance for 5C data when using small 12 kb distance bins, the dynamic distance bins used in our manuscript (detailed in Supplementary Methods), medium 24 kb distance bins, and long 60 kb distance bins (Rebuttal Figure 10). We ultimately find that final differential loop calls are similar and robust to distance window sizes. Finally, we highlight that we have added to the coding usage instructions a section describing how to modify distance bins if needed.*

6.	SI p.8. It seems the number of null replica shows up as above threshold is important for eFDR, but why the authors a priori choose the number of 6 replica? The authors should also specify how sensitive are the results to then number of replicates. As they are simulated, their generation should of very low cost. Thousands to even hundreds of thousands would be appropriate.

*Author Comment 2.14*

*We worked to explore this critical Reviewer point.*

*We first address the choice of six replicates per simulated replicate set:*

*This number was chosen to match that of the input replicate set so that transformation of simulated data matches that of the input replicate set as closely as possible. In this manuscript, we demonstrated 3DeFDR on a 5C data set that happened to capture three biological conditions with two replicates per condition to obtain a total of six replicates, and we generated null sets of six replicates as well.*

*We next address the question of how sensitive eFDR estimates are to the number of replicates:*

*We first address this in Author Comment 2.10 and Rebuttal Figure 8. In that figure, we show that as the number of replicate sets is increased, the returned eFDR estimate distribution becomes narrower with a central tendency still aligned at its expected value. Knowing this, a user might choose to initially call loops using a smaller number of replicate sets to save time on computation, largely because computation time, or required parallelized resources, does scale with number of simulations generated. When final publication quality results need to be generated, the user might then increase the number of replicate sets used for estimation substantially to obtain final eFDR estimates. We have noted this point in the usage instructions.*

*To address the point of exactly how many replicate sets are truly needed to lower sensitivity of eFDR to the specific randomly generated simulated counts in a set of null replicates, we provide Rebuttal Figure 11 below. We demonstrate that the variance of the eFDR estimates dramatically drop at approximately 20-25 null replicate sets. Beyond this number of number of null replicates, this variance drops far less dramatically with diminishing returns to further increasing the number of null replicates. We also note that these plots look very similar regardless of which background cellular model we use to create null sets.*

*To reflect this important challenge by Reviewer 2, in our re-submission, we have updated all 5C figures after re-running 3DeFDR loop calls for eFDR values computed over a null data set of 250 sets of six simulated replicates, or a total of 1500 simulated replicates.*

7.	It is unclear if the donut filter was applied to pre-process the diffHiC data. If not, then that could explain why diffHic was not specific for loops relative to 3DeFDR - in which case, the comparison made was then not a fair comparison.

*Author Comment 2.15*

*In this submission, we include a comparison of 3DeFDR-HiC to diffHiC on Hi-C data set. With this larger set of interactions, we are able to run diffHiC’s version of the donut filtering step, which they refer to as the peak filtering step, and include results of this pipeline in this submission. This comparison is now shown as Supplementary Figure 12, and it is also pasted below as Rebuttal Figure 12. The important findings from this analysis were that diffHiC remains vulnerable to calling loop stripes on the borders of domains instead of punctate corner loops even when peak filtering is enabled. This highlights the superiority of 3DeFDR as a method for specifically calling looping interactions over a more generalized differential interaction caller like diffHiC.*

8.	In the submission letter, corresponding authors mentions a "gold standard" set of loop interactions. However, there does not appear to be any mention of such a set in the main text, nor are there any standard comparisons against such a set (e.g. sensitivity, specificity, precision, recall, accuracy, AUCROC, AUCPR, etc). Is there actually a putative gold standard set of loops? If so, where are these comparisons against the "gold standard" set?

*Author Comment 2.16*

*The loops we refer to as “gold-standard” are individual loops that are well-established in the 3-D genome folding field in that they have been identified across every study with any technology. One such example is the Sox2-Super Enhancer loop in ES cells as well as the long-range Sox2-enhancer loops in NPCs. In our original submission, we visually demonstrated 3DeFDR’s ability to successfully call those loops using plots of loop call clusters. To address this reviewer’s concern, we now conduct 3DeFDR-HiC on a simulated positive control dataset in which we simulate differential loops genome-wide in Hi-C data at different effect sizes. We use this positive control to compute 3DeFDR-HiC’s ability to correctly recover these known loops and visualize these results in the form of an ROC curve. Details about our simulated positive control loop set and our subsequent calculation of 3DeFDR-HiC’s sensitivity and specificity are included in Figure 7, displayed in Author Comment 2.8, and described in our Supplementary Methods.*

9.The approach of emperical FDR estimation method is not novel. A well-known example is that of SAM (Significance Analysis of Microarrays): http://statweb.stanford.edu/~tibs/SAM/, which used bootstrap permutations to estimate FDR. The authors should acknowledge such earlier work.

*Author Comment 2.17*

*We have added this paper to the referenced literature in the manuscript.*

Minor Issues:

10. p.8 l.21-24. A citation here is needed.

*Author Comment 2.18*

*We could not find the location for this requested citation.*

11.	The statement on p. 8 that line 55 that "all confounders" have been corrected is an overstatement and needs to be modified.

*Author Comment 2.19*

*We have amended this statement.*

12. The assumption of log-logistic distribution, and hence p-value are also not justified.

*Author Comment 2.20*

*In the main text, we demonstrate 3DeFDR on the 5C data set published in Beagan et al. 2017, and in the supplement, we highlight that we transformed this data to obtain observed/expected values and p-values in a manner consistent with the transformation pipeline used in Beagan et al. 2017. Supplementary Figure 7 of Beagan et al. 2017, reproduced below, shows that the log-logistic distribution is well fitted to observed/expected values in every genomic region included in the data set. See below Rebuttal Figure 13.*

**Second round of review**

**Reviewer 2**

The authors have carried out extensive additional investigations and provided detailed response to address questions raised earlier. This resulted in interesting new findings, including the observations and justification of the authors' approach for short distance regime.

The thoroughness and seriousness the author invested in revision is much appreciated. Overall the manuscript has improved a lot, although there are some important intrinsic problems remaining unresolved. However, it is unlikely these problems will be fully addressed by others at this stage either, which the authors have now clearly explained and I agree.

Nevertheless, there are still a number of issues:

1. For detection of Hi-C loops, the authors chose the resolution at 10 Kb. However, loops are usually called at higher resolutions such as 5kb or 1kb. Loop calling at lower resolution likely are inaccurate, especially when one compares different cell types/conditions. The authors also mentioned in SI that loops with <40 kb are excluded. Why 10 kB and why this is not a problem? This seems to be a very serious limitation for Hi-C loop calling.

2. The comparison of simulated 5C count and real 5C data and reported good agreement (Fig 3) is not informative. As the goal of this study is to detect and call for changed loop interactions, reproducing overall patterns of 5C is not of interests. Rather, the authors should present the delta difference plots between simulated and cell-type conditions, and discuss how well condition specific loops can be called.

In addition, the authors may wish to discuss

3. Comparison with HOMER, which claims can also identify differentially regulated loops:

http://homer.ucsd.edu/homer/interactions2/HiCTADsAndLoops.html

**Authors Response**

**Point-by-point responses to the reviewers’ comments:**

*Below we provide a point-by-point response to the reviewer feedback on our manuscript “3DeFDR: Identifying cell-type specific looping interactions with empirical false discovery rate guided thresholding.” We thank the Reviewer for his/her thoughtful questions throughout the review process which have enabled us to improve the work.*

*Thank you for your time and input.*

*Best,*

*Jennifer E. Phillips-Cremins and Thomas Gilgenast on behalf of all authors*

Reviewer #2: The authors have carried out extensive additional investigations and provided detailed response to address questions raised earlier. This resulted in interesting new findings, including the observations and justification of the authors' approach for short distance regime.

The thoroughness and seriousness the author invested in revision is much appreciated. Overall the manuscript has improved a lot, although there are some important intrinsic problems remaining

unresolved. However, it is unlikely these problems will be fully addressed by others at this stage either, which the authors have now clearly explained and I agree.

Nevertheless, there are still a number of issues:

1. For detection of Hi-C loops, the authors chose the resolution at 10 Kb. However, loops are usually called at higher resolutions such as 5kb or 1kb. Loop calling at lower resolution likely are inaccurate, especially when one compares different cell types/conditions. The authors also mentioned in SI that loops with <40 kb are excluded. Why 10 kB and why this is not a problem? This seems to be a very serious limitation for Hi-C loop calling.

*Our 3DeFDR-HiC model does not have any inherent limitations with respect to the bin size of the differential loop analysis. For the results presented in the paper, we have chosen a bin size of 10 kb because we believe this bin size strikes a good balance between ability to identify small looping interactions on the one hand and the scarcity of Hi-C datasets that contain enough reads to be analyzed with bin sizes smaller than 10 kb without encountering issues related to insufficient coverage. The only published datasets we are aware of that are of sufficient depth to be analyzed with bin sizes smaller than 10 kb are Rao et al. 2014 (GM12878 only), Rao et al. 2017, and Bonev et al. 2017. We expect that the overwhelming majority of existing Hi-C datasets are of much lower read depth and do not contain enough reads to be binned with bin sizes smaller than 10 kb. We have added some text to the discussion to clarify this point. Regarding the exclusion of loops of total size < 40 kb – this is less related to the bin size and instead related to the limitations of calling loops that fall within the range of the distance-dependent background interactions (i.e. the so-called diagonal). The diagonal in Hi-C maps extends out from 0 kb to 200-400 kb, therefore callings loops within the first few bins of the diagonal is a computationally difficult problem – and we do not attempt to call anything as a loop smaller than 40 kb. In conclusion, 3DeFDR is flexible and can be adapted to work at any bin size and loop size the user chooses – and we emphasize that parameters should be adapted to the read depth of the data set in the user’s hands.*

2. The comparison of simulated 5C count and real 5C data and reported good agreement (Fig 3) is not informative. As the goal of this study is to detect and call for changed loop interactions reproducing overall patterns of 5C is not of interests. Rather, the authors should present the delta difference plots between simulated and cell-type conditions, and discuss how well condition specific loops can be called.

*According to the suggestions above, we explored the simulations and the real data. First, below, we present a heatmap showing the log2 fold change between simulated and cell type conditions (here, Klf4 condition):*

*This data demonstrates that there is little difference other than spatial noise between our real 5C maps and simulated replicates. Since our simulation model re-uses the real 5C mean value at each pixel of the heatmap, specific (in addition to overall) patterns like loops, domains, distance dependence, etc. are all fully present in our simulations.*

*We also highlight that we show a direct comparison in Figures 3A-D illustrating the preservation of specific and condition-specific 5C patterns in our simulated datasets.*

*Moreover, still focusing on the Klf4 region as an example, we also present the PDF and CDF plots of real and simulated data, as well as delta difference plots (difference between the CDFs) as requested by the reviewer. The data (below) demonstrate close similarity between real and simulated data sets.*

*Finally, we also assess how well our 3DeFDR-5C model calls condition specific loops, in relative terms, with the benchmark methods in Figures 5 and S4-S11.*

*For Hi-C data and 3DeFDR-Hi-C – we hold this method to an even higher standard than the above because we have the advantage of creating new simulations with known loops. We performed a more quantitative assessment of how well condition specific loops can be called in Figures 7 and S12-S13. We have added some text to the discussion to clarify this.*

In addition, the authors may wish to discuss

3. Comparison with HOMER, which claims can also identify differentially regulated loops:

http://homer.ucsd.edu/homer/interactions2/HiCTADsAndLoops.html

*We have added some text to the discussion to clarify that our comparison to other differential loop calling tools is not exhaustive and comparisons among all existing tools may be an area for future work.*
